# Seasonal Variation in Plant Polyphenols and Related Bioactivities across Three Years in Ten Tree Species as Visualized by Mass Spectrometric Fingerprint Mapping

**DOI:** 10.3390/molecules28166093

**Published:** 2023-08-16

**Authors:** Suvi Vanhakylä, Juha-Pekka Salminen

**Affiliations:** Natural Chemistry Research Group, Department of Chemistry, University of Turku, FI-20014 Turku, Finland; smhvan@utu.fi

**Keywords:** coniferous, deciduous, defenses, growing state, induction, mass spectrometry, phenolics, tannins

## Abstract

The currently changing climates and environments place plants under many types of stresses that affect both their survival and levels of chemical defenses. The gradual induction of defenses in stressed plant populations could be monitored on a yearly basis unless a seasonal and yearly variation in natural defense levels obscures such monitoring schemes. Here, we studied the stability of the species-specific polyphenol composition and content of 10 tree species over three growing seasons using five replicate trees per species. We specifically measured hydrolyzable tannins (galloyl and hexahydroxydiphenoyl derivatives), proanthocyanidins (procyanidins and prodelphinidins), flavonols (kaempferol, quercetin and kaempferol derivatives) and quinic acid derivatives with the group-specific UHPLC-DAD-MS/MS tool, together with two bioactivities, the protein precipitation capacity and oxidative activity. With the help of a fingerprint mapping tool, we found out that species differed a lot in their seasonal and between-year variation in polyphenols and that the variation was also partially specific to compound groups. Especially ellagitannins tended to have declining seasonal patterns while the opposite was true for proanthocyanidins. Some of the species showed minimal variation in all measured variables, while others showed even induced levels of certain polyphenol groups during the 3-year study. For every species, we found either species-specific baseline levels in qualitative and quantitative polyphenol chemistry or the compound groups with the most plasticity in their production. The used tools could thus form a good combination for future studies attempting to monitor the overall changes in polyphenol chemistry due to various biotic or abiotic stress factors in plant populations or in more controlled environments.

## 1. Introduction

Plants produce a wide variety of polyphenols and other specialized metabolites for their own protection against various types of biotic and abiotic stressors such as herbivores, pathogens and UV-B radiation. Global and local changes in all stress factors together with otherwise changing landscapes including competing plant and insect species, typically affect the levels of specialized metabolites in plants and the success or survival of plant individuals [1]. In the long run, during tens and hundreds of millions of years, these changes together with more dramatic effects such as the geological isolation of land parts and the coevolution of insect herbivores with flowering plants have resulted in the diversity of species in the plant kingdom we see today.

Currently, plant species diversity is increasingly challenged by unsustainable plant use practices, global warming, and biodiversity loss that is especially linked to insect diversity as well. All these in concert affect plants but also plant chemistry in ways that are not fully known to us as of yet. For instance, it has been shown that changes in insect herbivore abundance in plant populations may have dramatic effects on both plant chemistry, but also species microevolution [1,2,3] and that such changes in plant chemistry within a species reflect changes that have taken place in plant species evolution within that particular genus [4]. Given the current magnitude and speed of change in just insect species distribution, abundance and diversity [5,6], it would be enlightening to follow how these affect the chemistry of plants and plant populations over time.

Plant chemistry shows and has always shown seasonal changes in natural environments. Plants have a plethora of natural enemies some of which accumulate in the spring when the green plant tissue is at its softest and perhaps also most nutritious stage, while others prefer ecological windows closer to summer or even autumn [7]. For these reasons, some defense compound groups show variations in their seasonal appearance and especially content [8,9,10]. This natural variation should be known or we should know the stage of the growing season when to sample plants at the minimal natural variation of defense compounds. Otherwise, we cannot find a difference between natural variation and variation caused by abnormal events linked to the more dramatic changes in our landscapes and environment.

In this study, we sampled leaves or needles of five individuals per ten tree species three times during a season and replicated the sampling over three subsequent years. We intentionally avoided collecting samples during early spring and summer, i.e., the point of most significant change in plant chemistry [11,12,13]. Instead, we wanted to monitor how plant chemistry changes once leaves and needles have reached their mature size, but still continue growing their toughness and mass. In Southwestern Finland this time corresponds to late May–mid-June. The other sampling points were separated by one month each year. We focused on measuring plant polyphenols and related bioactivities since our research group has good background knowledge on how changes in plant polyphenol structure affect their bioactivities ranging from oxidative activity [14,15,16,17,18], protein precipitation capacity [19,20,21,22], nature of tannin-protein complexes [23], covalent linkage to proteins [24], ruminal fermentation [25] and anthelmintic activity [26,27].

In addition, with this diverse sample set we were able to test the functioning of our novel approach utilizing the visual mass spectrometric fingerprint maps of polyphenols [28] obtained for the main polyphenol groups of plants after their group-specific UHPLC-MS/MS analyses [29,30,31]. Finally, this approach is able to link all the detected polyphenol and bioactivity levels to their overall levels found in thousands of plant species from five continents (see Materials and Methods for details). It is thus much easier to make conclusions about the proportional significance of the baseline levels found for each polyphenol group in different species. This is because low baseline levels may offer more plasticity for the induced production of given polyphenol groups (e.g., ellagitannins, ET), especially when biosynthetically competing polyphenol groups are not overexpressed (e.g., proanthocyanidins [32,33]). In most cases, our mass spectrometric fingerprint mapping approach allowed finding the structural and biosynthetic reasons for differences in plant bioactivities further highlighting the importance of knowing the metabolites groups detected. In general, we found a combination of two types of species-specific fingerprint maps for each species, and the seasonal and within-species variation gave us good knowledge of the species-specific accuracy or repeatability of the stable baseline levels for each variable measured. It remains to be seen, if these and other types of tools are used in the future for the monitoring of changes taking place in the chemistry of the plant kingdom, but at least here we offer one set of tools to be used in this important task.

## 2. Results and Discussion

Recently, we developed a tool for the visual mapping of the qualitative and quantitative data of the main polyphenol groups and related bioactivities detected in 31 plant species [28]. We detected functional units of the compounds belonging to the most common polyphenol groups with MS/MS tools to record the whole fingerprint of galloyl (G) and hexahydroxydiphenoyl (HHDP) derivatives (hydrolysable tannins (HT)), procyanidin (PC) and prodelphinidin (PD) derivatives (proanthocyanidins (PA)), kaempferol (KA), quercetin (QU) and myricetin (MY) derivatives (flavonols (FL)) and quinic acid (QA) derivatives [29,30]. The UHPLC-UV and group-specific UHPLC-MS/MS fingerprints of each studied species are presented in Figures S1–S10. To study the linkage of certain types of polyphenols and bioactivity levels we measured two important polyphenol-related bioactivities: the protein precipitation capacity (PPC) and oxidative activity (OX).

We showed that the chosen variables acted as chemotaxonomic markers discriminating the species from each other and that the observed polyphenol patterns explained the bioactivity levels of the species as well. Here, we used this tool to study the variation of these same variables in 10 woody plant species over three growing seasons to see if the patterns observed at a single stage of a growing season are stable as the season progresses and as years change. Moreover, we followed the patterns also for individual trees to see how much variation individuals bring to the big picture or if some of the patterns with individuals differ a lot from those seen with species averages. Below, we go through these results species by species and then make general conclusions on the seasonal patterns emerging from all of the species as a whole. Some of the patterns matched those observed earlier for the species while many were witnessed for the first time.

### 2.1. Seasonal Patterns in the Foliage of Betula pubescens

**Figure 1:**
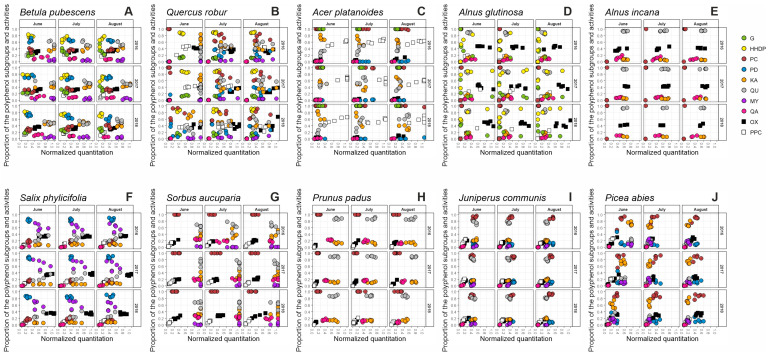
Seasonal and yearly changes for the ten tree species in the fingerprint maps of their polyphenol subgroups and bioactivities (G, gallic acid derivatives; HHDP, hexahydroxydiphenoyl derivatives; PC, procyanidin units; PD, prodelphinidin units; KA, kaempferol derivatives; QU, quercetin derivatives; MY, myricetin derivatives; QA, quinic acid derivatives; OX, oxidative activity; PPC, protein precipitation capacity), as measured from the leaves (**A**–**H**) or needles (**I**,**J**) of five individual trees three times during the growing seasons of 2016–2018. The *x*-axis shows the normalized concentrations of hydrolyzable tannins (G + HHDP), proanthocyanidins (PC + PD), flavonol derivatives (KA + QU + MY), quinic acid derivatives, oxidative activity and protein precipitation capacity. The *y*-axis shows the proportions of the subgroups belonging to these main polyphenol groups (G/HHDP, PC/PD, KA/QU/MY) or the proportions of QA, OX and PPC of the total phenolic levels recorded in the sample. The detailed flavonol fingerpint maps of white birch flavonol derivatives were intriguing (Figure 2A), since kaempferol and myricetin glycosides were dominated by other types of patterns than the quercetin glycosides. Earlier Engström et al. [30] showed how e.g., quercetin 3-*O*-glycosides produce different patterns from the quercetin 7-*O*-glycosides, depending on the ratios of the aglycone ions and radical ions formed during the glycoside fragmentation. However, also the B-ring methylation affects which ion types are formed (Salminen, unpublished data) and thus these patterns (Figure 2A) cannot be exclusively used to predict the glycosylation types in the FL groups, as they are also affected by other types of decorations of the core aglycones. Nevertheless, in 2018, these two groups of quercetin glycosides were clearly separated in June and July, but in August they aligned the patterns seen for quercetin glycosides during all of the three months of 2016 and 2017. This suggests that differently decorated QU derivatives were evenly distributed in all other time points, but not in June and July of 2018.

Figure 1A shows the x-y maps for the galloyl and HHDP derivatives (G + HHDP, i.e., HT), procyanidins and prodelphinidins (PC + PD, i.e., PA), kaempferol, quercetin and myricetin derivatives (KA + QU + MY, i.e., FL), quinic acid derivatives (QA, most often coumaroyl, caffeoyl and galloyl quinic acids) together with oxidative activity (OX) and protein precipitation capacity (PPC) in the foliage of five replicate *B. pubescens* trees at three stages of each of the seasons. Each *x*-axis of the plots shows the quantitative data for G + HHDP, PC + PD, KA + QU + MY, QA, OX and PPC after their normalization according to the quantitative variation observed in our larger plant species screening experiment (Vanhakylä, Salminen et al. unpublished data; see also Section 3). This normalization helped us to visualize if the values of these six main groups of compounds and activities are e.g., low (<0.2), intermediate (0.4–0.6) or high (>0.8) when compared with similar data obtained across thousands of species collected from five continents. This means that when purple, orange and grey dots of the three flavonol groups were at their maximum normalized level of 1.0 in some *B. pubescens* trees during especially June 2016 and 2017 (Figure 1A), these total flavonol levels were among the best 5% of the total flavonol levels detected in the big species set. However, it does not mean that flavonols were the main group of polyphenols in *B. pubescens*, since proanthocyanidins typically accumulate at much higher levels in the plant kingdom than flavonols do, meaning that the normalized 0.4 levels of PAs are in fact higher in mg/g than the 0.8 normalized levels of flavonols. The mg/g values of each compound group and activity are separately shown in Table S1.

Our results showed a clear decline in the concentration of hydrolyzable tannins, especially during the summer of 2016 and 2018 (Table S1, Figure S11A). The overall decline from June to July and August was also statistically significant (*p* < 0.001) for both G and HHDP derivatives. This type of decrease was earlier detected especially for galloyl glucoses and for some monomeric ellagitannins in white birch, *B. pubescens* [8], but also its subspecies *B. pubescens* subsp. *czerepanovii* [34] and in *B. nana* and *B. pendula* [35]. However, previous studies showed considerable differences in the seasonal variation of individual ETs as especially the smallest ETs accumulated towards the autumn. These were breakdown products of the larger ETs via cleavages of either their galloyl and/or HHDP groups. This process naturally affects the amount of galloyl and/or HHDP derivatives to be detected by our group-specific MS/MS tools. This can be seen in Figure 1A as the proportion of yellow HHDP dots tended to decrease and the green galloyl dots increase especially during 2016 and 2017, but not so much during 2018. Such proportionally larger changes in HHDP derivatives are natural even without HHDP cleavage since HHDP-rich ETs are the main ETs of white birch and their naturally occurring seasonal decline affects the HHDP share proportionally more than the G share of birch HTs [8]. Still, looking at the seasonal patterns observed for individual trees (Figure S11A), it is clear that for some individuals the decrease in both G and HHDP was marginal or non-existing during 2017 (white birches 2, 4 and 5), while in 2016 and 2018 all five trees did drop both their G and HHDP content. Only birch number five differed significantly (*p* < 0.001) from all of the other individuals when galloyl content was considered and from three other birches by its HHDP content. The year 2018 differed from 2016 and 2017 by the galloyl content and from 2016 by the HHDP content (*p* < 0.001). This highlights the complexity earlier witnessed in the seasonal patterns of white birch HTs, now for the first time evidenced between the years as well.

The other group of white birch tannins, i.e., proanthocyanidins showed an opposite seasonal trend to HTs. Their content did not decrease but rather increased, although there was significant variation in this trend between years and individual trees (Figure 1A, Table S1, Figure S11B, Table S14). Previous studies with mountain birch have shown seasonal increases in total PAs [34,36,37], and now we showed that at least in 2016 and 2018 this increase was mainly due to the PC units rather than the PD units of PAs; as the total PAs increased, the PC/PD ratio changed from 30/70 to 40/60. Nevertheless, both changes in PC and PD content from June to August were statistically significant (*p* < 0.001 and *p* = 0.016). Just like with HTs and the G/HHDP ratio, also the changing PC/PD ratio must have clear consequences to the birch leaf bioactivities since HHDP and PD groups are much more important to oxidative activity than are G and PC groups [14,15]. However, oxidative activity did not show significant seasonal patterns, suggesting that in white birch the seasonally more stable PD units of PAs might be more important to oxidative activity than the decreasing HHDP-containing ETs, all of which are monomeric HHDP esters that as such are one of the weakest ET types concerning their oxidative activity at high pH [15].

There were some additionally interesting patterns observed for individual trees. For instance, the white birch 5 which produced the lowest G and HHDP levels throughout the seasons and years, stepped up in its PA production from June to July. It was the worst PC and PD producer in June 2017 and among the worst in June 2016 and 2018, but then even tripled its PA content to become the best PC and PD containing white birch in July 2016, 2017, and 2018. This ability to massively increase PA production could have been due to its very low HT content since high HT production directs biosynthetic energy away from the phenylpropanoid pathway and further PA and flavonoid biosynthesis [33]. This is consistent with the findings of Riipi et al. [34], whose results show that mountain birch individuals invested either in HTs or PAs because of the limited resources from the shared biosynthetic pathway. In accordance with this hypothesis, white birch 5 was the only one increasing its flavonol content in June–July 2017, the same period when both the PC and PD content of that tree more than tripled. This could be due to the upregulation of enzymes controlling the closely linked flavonol and PA pathways (e.g., [38]). Otherwise, all the flavonol levels in general dropped during the seasons as earlier shown by Raal et al. [39] and Riipi et al. [36]. The decline was significant from June to July (*p* < 0.05) and highly significant from June to August (*p* < 0.001) for every flavonol group (Table S14). For quinic acid derivatives the white birch 5 again showed opposite patterns to the other four trees in 2017 and 2018 (Figure S11A,C). These facts highlight how general species-specific patterns can be sometimes challenged by looking at patterns at the scale of individual trees.

### 2.2. Seasonal Patterns in the Foliage of Quercus robur

The general seasonal patterns of decreasing HTs, increasing PAs and decreasing FLs were the same for *Q. robur* as they were for *B. pubescens*. However, for English oak these patterns were clear without any exceptions as all oak trees had clearly decreasing G and HHDP content and increasing PC and PD content (Figure 1B and Figure S12A,B, Table S2), even though some individuals differed statistically from each other (Table S15). All these changes were highly significant between June and July and June and August (*p* < 0.001) implying a more rapid change after June than in July (Table S15). With some oak trees, the total PA concentration increased from nearly zero to even 0.8 at the normalized concentration scale. While PCs were the dominant type of units present in oak PAs, the proportion of PD units seemed to increase slightly from June to August together with the average size of PAs. This was the opposite pattern to *B. pubescens* PAs which were PD-rich and tended to increase their PC share during the season. Of the HTs, English oak was even more HHDP-rich and galloyl-poor than white birch, since it is known to produce C-glycosidic ETs as its main polyphenols. However, these ETs such as the monomeric castalagin and vescalagin and the dimeric cocciferin D_2_ all contain nonahydroxytriphenoyl (NHTP) units in addition to the HHDP units. These NHTP units are not detected by our group-specific tool. For that reason, our total HT levels in the leaves of English oak can be a little underestimated. Nevertheless, the relatively high content of C-glycosidic ETs during the seasons produced much higher levels of oxidative activity in *Q. robur* leaves, since these ETs have been repeatedly shown to be much more easily oxidized at high pH than the monomeric glucopyranose-based HHDP esters produced by *B. pubescens* (e.g., [14,15]). The protein precipitation capacity was at intermediate levels and a little lower than the normalized oxidative activity levels. This is also in agreement with the C-glycosidic ETs being relatively poor in their PPC while much better in their OX [20]. This PPC vs. OX pattern tended to shift from totally resolved black and white dot clusters in June 2016, 2017, and 2018 to partially overlapping clusters in especially August of all years. This was a logical shift due to decreasing ET (higher OX) levels and increasing PA (lower OX) levels during all these years.

**Figure 2:**
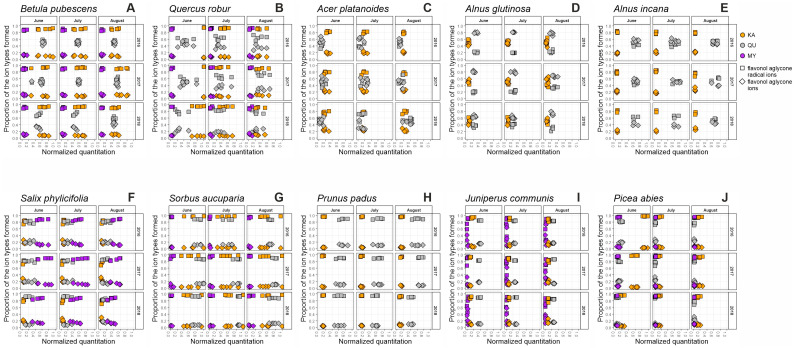
Seasonal and yearly changes for the ten tree species (**A**–**J**) in the detailed fingerprint maps of the three flavonol subgroups (KA, kaempferol derivatives; QU, quercetin derivatives; MY, myricetin derivatives) including the proportions of the aglycone radical ions (squares) and aglycone ions (diamonds) formed during the fragmentation of the original flavonol derivatives in the MS ion source before the specific MRM detection of the flavonol subgroups. The *x*-axis shows separately the normalized concentrations of KA, QU and MY derivatives in the five replicate plants. The *y*-axis shows the proportions of the aglycone radical ions to the aglycone ions detected from the KA, QU and MY derivatives found in the corresponding plant individuals.

The total FL concentration was high especially in June 2016 and 2017 reaching the maximal normalized level of 1.0 (Figure 1B). It dropped significantly during the seasons even to ~0.3 level in August 2016 and 2018 (Table S15). During this concentration shift, we witnessed an interesting pattern: the dominating FLs in early summer were KA derivatives (~50–80%) while QU derivatives were dominant in August (~40–70%). Figure 2B shows how this shift was mainly due to the decrease in KA derivatives as the QU derivative content decreased only marginally. Similar to *B. pubescens*, only the QU derivatives showed changes in their detailed fingerprints, i.e., in their decoration patterns.

Many of these main patterns of seasonal change were seen in earlier studies as well, such as the negative association of HTs and PAs and the decrease of FLs [9]. The decline in HT concentration was also detected by Visakorpi et al. [40] in most of the experimental sites and treatments, but the flavonol content did not follow a certain seasonal trend. The decreasing total flavonoid content was detected by Tálos-Nebehaj et al. [41], who also reported increased flavan-3-ol levels in early summer and a decrease towards August.

### 2.3. Seasonal Patterns in the Foliage of Acer platanoides

The Norway maple (*A. platanoides*) was the third and last species in our study producing both HTs and PAs in leaves of all tree individuals (Figure 1C and Figure S13A,B, Table S3). However, its HT and PA composition differed significantly from the two earlier-mentioned species, *B. pubescens* and *Q. robur*. For HTs, it did not produce any ETs but only gallotannins such hexa-, hepta- and octagalloylglucose and simple gallic acid derivatives such as tetra- and pentagalloylglucose [17]. These gallic acid derivatives did not show drastic seasonal changes (*p* = 0.126) as their content only slightly decreased during 2016 and 2017 or slightly increased in 2018 (Table S16). For PAs, the Norway maple did not produce a PC/PD-mixture like white birch and English oak, since its PAs were practically PC pure. However, in line with birch and oak, the maple PAs showed a clear seasonal pattern with constantly increasing concentration in all of the other studies points aside from August 2017. The statistical results showed again the most drastic change after the earlier parts of the summer (Table S16).

The QA derivatives of *A. platanoides* showed a clear seasonal decline (Figure 1C and Figure S13A), while any of the FL subclasses did not show any highly significant pattern within seasons or between years as the FL concentration from June to August was highly dependent on the year (Figure 1C and Figure S13C). However, the QU derivatives again increased their proportional significance against the KA derivatives, especially in 2016 and 2018. Earlier, Tálos-Nebehaj et al. [41] detected gradually increasing amounts of total flavonoids with the season, a pattern we showed for total flavonols only in 2018. The contents of FLs and other non-tannin polyphenols were quite low and thus contributed only marginally to the two Norway maple bioactivities that were dominated by PPC over OX. Both gallotannins and PC-rich PAs are known for their relatively low OX, but these tannin subgroups have decent PPC and for that reason, individuals in this species clearly outperform with their PPC rather than with OX.

### 2.4. Seasonal Patterns in the Foliage of Alnus glutinosa

To our knowledge the seasonal variation of *A. glutinosa* phenolic compounds has not been studied with modern methods. Only Veselá et al. [42] reported in their study that total phenolics remained at an almost stable level from June to October with only a slight decrease during the season. Figure 1D and Table S4 show the variation observed with our group-specific MS/MS and bioactivity tools. The most variation was brought about by HTs that showed non-uniform presence/absence data and seasonal patterns in the foliage of individual black alder trees (Figure S14A). One of the trees accumulated even 70 mg/g HHDP derivatives while 2/5 of the trees never exceeded the 0.1 mg/g level. The one ET-dominant tree had every year seasonally decreasing G and HHDP content while one of the trees tended to accumulate G and HHDP derivatives towards August. Another three had the highest HHDP levels in July, but only during 2017. In general, it seemed that there was higher variation between individuals than within the season as such. This was proved to be true with G and HHDP derivatives, as the differences between individuals were statistically highly significant (*p* < 0.001) and months did not differ from each other (Table S17). The same goes for PAs, FLs and QAs that did not show any clear seasonal patterns, but rather high variability between individuals and years. An interesting phenomenon was that the three trees that were able to produce clear levels of HTs also produced the largest levels of PAs (Figure S14B). However, these PA levels were still very low (2–3 mg/g), as can be seen by the <0.1 levels for the normalized quantitative data. Such low levels do not yet cause much biosynthetic pressure for the competing HT pathway, in comparison to *B. pubescens*, *Q. robur* and *A. platanoides*, which produced even 30 mg/g, 60 mg/g and 40 mg/g levels of PAs, respectively. Perhaps such low levels of PAs in *A. glutinosa* give more flexibility for its HT synthesis when needed, since the HHDP levels varied a lot between the years in the two best HT-producing individuals: tree 4 changed its HHDP levels from 25 mg/g via 70 mg/g to 60 mg/g and the tree 1 from 5 mg/g via 50 mg/g to 10 mg/g during years 2016, 2017 and 2018, respectively. Presumably, such inducible and high levels could not be easily achieved, if the PA levels would be simultaneously high as well. Changes in flavonol derivatives of individual trees are presented in Figure S14C.

### 2.5. Seasonal Patterns in the Foliage of Alnus incana

The grey alder was quite different from the black alder in its seasonal patterns (Figure 1E, Figure 2E and Figure S15A–C, Table S5), mainly because its individuals did not produce any HTs, although grey alder is known to be capable of their production (e.g., [43]). This fact further highlights the between-tree variability of HT biosynthesis in the two *Alnus* species included in this study. Because of the lack of HTs, the fingerprint maps of grey alder looked very stable across the seasons. It did have a bit higher FL and QA levels than black alder and the more detailed KA and QU derivative fingerprints (Figure 2E) clearly separated these alder species from each other. The total QA levels dropped during 2016 and 2018 but increased slightly in 2017. Unfortunately, the missing individuals after tree cutting in July 2017 prevented us to perform reliable statistical tests. Earlier, Kotilainen et al. [44] reported decreasing concentrations of chlorogenic acid isomers after the highest levels in the early summer. Since the QA derivatives of *A. incana* are mainly caffeoyl quinic acids, our QA data support this pattern during 2/3 of the years. Although the PA levels were low in grey alder as well, they still showed the same generally increasing seasonal content as all the other species above. Kotilainen [44] had shown much higher PA levels (20 mg/g) that increased gradually with the season and then dropped before abscission. The low PA levels in our *A. incana* individuals were not able to produce any PPC response and the lack of HTs did not help either. The oxidative activity patterns were quite stable between the nine sampling times (Figure 1E), although a little bit lower than for the individuals of *A. glutinosa*.

### 2.6. Seasonal Patterns in the Foliage of Salix phylicifolia

Our results show that the leaves of the tea-leaved willow are especially rich in flavonoid-based compounds that have a trihydroxy substitution in their B-ring, i.e., PD-type PAs and MY-type FLs. Both of these are known for their relatively high oxidative activity which can be seen by the high OX levels in Figure 1F as well [18]. The willow PAs showed a clear seasonal increase (*p* < 0.001) during all years studied (Table S18). This increase was more due to PD than PC units as the proportion of PD units seemed to increase in PAs from June to August. At the same time, all the FL groups showed either a stable or slightly decreasing trend, although for KA and QU derivatives we did not find any statistically notable seasonal pattern (Figure 1F and Figure S16B; Table S18). Also, all the flavonol derivatives showed high variation between individual trees (*p* < 0.001). This could be due to QU and MY derivatives in *S. phylicifolia* being sensitive to elevated UV radiation as shown by Tegelberg et al. [45] and since the natural environment of *S. phylicifolia* individuals varied from partially shady to sunny. In addition, *S. phylicifolia* is a dioecious species with male and female flowers on separate individuals. This may cause variability between genders, as male and female trees are expected to allocate resources differently to reproduction and growth [46,47]. In our study, the individuals were selected randomly, and the gender was not determined, which can partially cause the variation in the data (Figure 1F, Figure 2F and Figure S16A–C). However, the above type of variation was not observed in PC or PD-type PAs, which had higher content than the FLs, meaning that the resource allocation hypothesis is an unlikely explanation for FL variation and that PAs do not seem to be as sensitive to environmental factors than FLs at least in this *Salix* species.

The tea-leaved willows contained no HTs, meaning that the relatively low PPC values were due to PAs only. However, as the PA content together with the average PA size increased with the season reaching as high mean degree of polymerization values as 20, also the PPC values approximately doubled from 0.1 to 0.2 normalized values every year from June to August. The seasonal trend with OX did not follow that of PPC, since OX depended in addition to the PD and MY levels, on the levels of dihydromyricetin. This dihydroflavonol or flavanol is not detected by our myricetin-specific MS/MS tool, but it is still the main individual compound in *S. phylicifolia* and easily oxidized due to its trihydroxysubstituted flavonoid B-ring [18]. We looked at the seasonal variation of this main compound separately and it showed a 23% decrease in concentration between June and July and even a 49% decrease between June and August, thus at least partially explaining the drops in OX during the seasons.

### 2.7. Seasonal Patterns in the Foliage of Sorbus aucuparia

The fingerprint maps of *S. aucuparia* show an exceptionally high normalized concentration of flavonol derivatives from 0.6 to 1.0 (Figure 1G), the species being thus among the best 5% of flavonol-producing species of the larger species set (Vanhakylä, Salminen et al. unpublished data). Also, earlier studies have reported high concentrations of flavonoids in the leaves of *S. aucuparia* and other *Sorbus* species and stated them to be one of the main components responsible for the bioactivities of the species [48,49]. Any of the flavonol groups did not show specific seasonal patterns (Figure 2G and Figure S17C, Table S19), but the KA derivatives had relatively large variation between the rowan individuals (*p* < 0.001) and this variation remained similarly large within the seasons and years.

The other group that was particularly high in its normalized concentration was the QA derivatives. *S. aucuparia* is especially rich in caffeoyl quinic acids such as 3-*O*-caffeoyl quinic acid and 5-*O*-caffeoyl quinic acid [49,50]. In total, *S. aucuparia* QA derivatives stayed at the 20% level of total phenolics across the seasons and did not show any repeatable patterns in their content as their content decreased a little in July 2016 and August 2018 (Table S19). The other variables shown in Figure 1G did not show specific seasonal patterns either and even the PC-dominated PA levels were surprisingly stable across the seasons, given that in other species this compound group had shown a uniformly increasing seasonal trend. Recently, Enri et al. [51] reported a minor increase in PA levels after the beginning of June and then stable levels until the end of August, while Olszewska [50] showed increasing PA levels between May and July, then decreasing towards August. These non-uniform patterns agree with our PA patterns as we saw a decrease/increase in 2016 and a minor increase in 2018, but stable levels in 2017 (Figure 1G and Figure S17B). All in all, it can be said that the overall fingerprint maps of *S. aucuparia* were very reproducible across the seasons, just like with *A. incana* above.

### 2.8. Seasonal Patterns in the Foliage of Prunus padus

Our fingerprint maps (Figure 1H and Figure 2H) show a nearly stable level of FL across the study period, with a small decline in concentration especially during the summer of 2018. A decline in total flavonoid content has been detected also in previous studies of *P. padus*, especially rapidly after flowering from May to June and more gradually after that [52]. The steady FL pattern was shown to continue in later autumn as well by Mattila et al. [53]. QA followed a decreasing trend in 2016 and 2018; this could be due to declines in chlorogenic acid, the main QA derivative in *P. padus* [52]. The PAs were again practically PC pure and showed a slight increase towards autumn in 2017 and 2018, also seen in *P. padus* by Olszewska and Kwapisz [52], but also a stable or declining content in 2016. These variable year-specific PA patterns were caused by individual trees having their own patterns (e.g., three declining and two increasing their PA content in 2016) that made the species-specific patterns almost non-existent (Figure S18B). Altogether, the variation between individuals and years seemed to be more significant than the seasonal variation in *P. padus* (Table S20).

### 2.9. Seasonal Patterns in the Needles of Juniperus communis

The common juniper was one of the two conifers included in our study. Earlier studies with junipers and other conifers have shown differences in phenolic composition of first-year needles compared to older needles [54,55,56]. This highlights the importance of comparable sampling over the years. Our sampling was conducted always on new-growth needles to avoid possible differences caused by different maturity stages. Overall, the fingerprint maps (Figure 1I and Figure 2I) remained relatively similar across the growing seasons and years. The main polyphenol group was PA with an average PC/PD ratio of 90/10. Interestingly the PC and PD units of the PAs showed variable seasonal patterns, e.g., in 2018 the PC content increased by ~20% while the PD content went down by ~25%. The average size of PAs decreased and their concentration increased slightly from June to August in 2017 and 2018, but not in 2016. Altogether, the seasonal variation of PC was moderately significant between June and August (*p* < 0.010) but the difference between individuals was clearer (*p* < 0.001).

The flavonol concentrations of new growth needles had an opposite general trend to PAs as they decreased slightly during the summer from ~0.4 normalized concentration to <0.3. The seasonal changes were significant for all the flavonol groups (Table S21). A similar result in flavonol content was also seen in another study of Finnish junipers [57]. These opposite trends with PAs and FLs were easily seen in the fingerprint map as they tended to group in June as PC + QU and PD + KA + MY, but then these groups diverged in July and August as PC and PD plots moved to the higher and QU + KA + MY plots to the lower normalized concentration areas. The reason why these slight concentration shifts were so well visible in Figure 1I is the highly reproducible quantitative results between the individual *J. communis* trees. The same was true also for the qualitative data, since just like the PC/PD ratio; also the KA/QU/MY ratio stayed nearly constant and QU-dominated throughout the season (Figure 2I). The most dramatic seasonal shift was seen with the QA derivatives, as their relatively low content dropped by ~80% each year from June to August and the decline was significant between every month (Table S21). The concentration patterns of individual trees are presented in Figure S19A–C.

### 2.10. Seasonal Patterns in the Needles of Picea abies

The Norway spruce is an evergreen coniferous tree, which grows new shoots every year. The variation in phenolic compounds outside the growing season has been reported to be minimal [58] as the new growth needles reach the chemical content of mature needles in September [59]. However, our seasonal data with new growth needles show a detectable variation for all of the measured variables as can be seen in both Figure 1J and Figure 2J and in Table S10.

Similar to the common juniper, the needles of the Norway spruce showed significantly declining seasonal content of both QA and FL derivatives (*p* < 0.001, Table S21). However, the FLs were dominated by the KA and not the QU derivatives. All FL subgroups declined much more rapidly than in the common juniper especially from June to July, since the shift from July to August did not alter the FL content. These rapid changes in KA glycosides after June were also detected by Slimestad [58] and Ganthaler [59]. This rapid shift was also highlighted in the detailed FL data (Figure 2J) that showed high KA levels in June 2016, average levels in June 2017 and the lowest levels in June 2018. This gradual change between the years in June resulted in no more change in the KA levels between June, July and August of 2018. It is possible that in 2018 the highest KA levels occurred earlier than in other years and we missed those with our sampling scheme.

PAs were the main polyphenols of *P. abies* needles with a slightly higher average PD content than in *J. communis*. This was due to the larger within-species variation as the PC/PD ratio varied, e.g., in June 2016 from 55/45 to 95/5 (Figure 1J). The differences in PC and PD content between individual trees were highly significant (*p* < 0.001). The one individual *P. abies* tree with the high PD content had each year the lowest PC content and was each year among the two best PD producers (Figure S20B). Just to highlight the variation between individuals in the PC + PD production, tree 4 was among the two best PD producers in 2017 and 2018 and was also among the best PC producers during these same years. This way, the PC + PD combinations were not as well grouped in Figure 1J as they were with *J. communis*. In addition, the seasonal patterns with PAs were not as straightforward as shown by Virjamo and Julkunen-Tiitto [13]. In their study, *P. abies* needles had the highest amount of PAs in developing shoots. In our study, this was true for 5/5 individual trees in 2016, for 3/5 in 2017 and for none in 2018. In fact, in 2018 the PA content increased at least by 50% for 4/5 trees, suggesting that the between-year variation in the Norway spruce needle PAs will be much more difficult to predict than for *J. communis* or for the foliage of deciduous trees included in this study. Ganthaler [59] reported similar non-consistent seasonal results with catechin and gallocatechin, the monomeric building blocks of PCs and PDs. From the bioactivity point of view, our PPC data seemed to follow the total PA trend showing a clear decrease followed by an increase in 2016 and 2017. In 2018 the PPC trend was ascending as also seen with the PAs. The oxidative activity increased by 64%, 94% and 152% during the growing seasons of 2016, 2017 and 2018, respectively, and these increases did not seem to be linked to any measured polyphenol groups as such. However, when we looked at the UV chromatograms of the UHPLC-DAD-MS/MS runs, we noticed a major peak appearing in all of the *P. abies* extracts corresponding to the July and August samples. This peak was almost absent in the June samples and it was identified as astringin, a stilbenoid glucoside with catechol-type phenolic moiety earlier shown to possess oxidative activity [18]. The content of astringin increased a lot towards July and was even four times higher in August than in June. Since we did not see other major changes in the UHPLC-UV chromatograms of *P. abies* needles, we interpreted that the seasonal increase in oxidative activity could indeed be linked to this dramatic increase in the levels of astringin.

## 3. Materials and Methods

### 3.1. Studied Plant Species

The plant sampling is a continuum to our earlier study of 31 Finnish plant species and their polyphenol content within a population [28]. Ten woody species were selected on the basis of their diverse types of combinations of phenolic content and biological activity. Our data includes two evergreen gymnosperm species (*J. communis* (Cupressaceae) and *P. abies* (Pinaceae)) and eight deciduous angiosperm tree species *(A. platanoides* (Sapindaceae), *A. glutinosa* (Betulaceae), *A. incana* (Betulaceae), *B. pubescens* (Betulaceae), *P. padus* (Rosaceae), *Q. robur* (Fagaceae), *S. phylicifolia* (Salicaceae), *S. aucuparia* (Rosaceae)). The classification of the species is according to Christenhusz et al. [60] and the Angiosperm Phylogeny Group IV [61]. The plants were collected from several locations around Turku and Kaarina areas in South-West Finland (Table S11).

White birch, (*B. pubescens*) is a deciduous tree, which has the northernmost distribution range compared to any other broadleaf tree. It has economic value for its timber, sap and substances, e.g., for cosmetics, but it also has traditional uses as herbal remedies and birch bark crafts.

The English oak (*Q. robur*) is a relatively long-living deciduous tree. It is an ecologically important species supporting a high diversity of insects and pathogens. Pedunculate oak is highly valued for its timber and traditional medicinal uses. Many of the species’ important properties are based on its high tannin content.

The Norway maple (*A. platanoides*) is a deciduous tree with relatively large leaves and a dense crown. It is commonly used as an ornamental plant in urban environments because of its drought tolerance, shading leaves and beautiful autumn colors. The genus Acer has been used in traditional medicine because of its several biological activities. Most of these activities are based on phenolic compounds [62].

*A. glutinosa*, engl. common alder or black alder, is a typical deciduous tree on wet habitats like sea and lake shores and streamsides. It is in association with nitrogen-fixing bacteria in its root nodules, which enables it to grow in very poor soil types. Interestingly A. glutinosa drops its nitrogen-rich leaves still green in autumn enriching the soil. Nitrogen-fixing and soil quality regulation seem to be the main chemical attributes of the species. Also, bark and acorn extracts and their biological activities have been studied in more detail (e.g., [63,64,65]).

*A. incana*, engl. grey alder, is usually a shrubby tree with several trunks. Compared to its relative species *A. glutinosa*, it favors less moist habitats but tolerates also wet soils. The nitrogen-fixing is an important feature also for *A. incana* and it can be used to afforest non-fertile soils.

*S. phylicifolia* (engl. tea-leaved willow) is a deciduous shrub or a medium-sized tree growing mainly on riverbanks and other moist habitats. The genus Salix has a long history of medical use as an anti-inflammatory agent and a pain relief around the world [66]. Although the genus is most commonly recognized for its high salicylate levels and related medical uses, the other specialized metabolites, especially phenolic compounds, appear to have a wide range of significant biological activities [67]. Interestingly, the leaves of *S. phylicifolia* are practically salicylate-free compared to other *Salix* species [45,68]. Instead, the leaves of *S. phylicifolia* are very rich in flavonoids [69]. This implies that different *Salix* species have alternative chemical defense strategies against herbivores.

*S. aucuparia* (engl. rowan) is a variable deciduous species, which can grow in various environments and is also grown as an ornamental tree. Its growing habit can vary from single to several tree trunks. It is best known for its sour berries, which can be used, for example, to make jelly and alcoholic beverages, but they have also been widely used as folk medicines.

*P. padus*, engl. bird cherry is a deciduous shrub or a small tree, which produces edible stone fruits. Plant extracts from different parts of the plant have been used as ethnomedicines and are known to have, for example, anti-inflammatory effects and antioxidant activity [70,71].

Common juniper (*J. communis*) is an evergreen coniferous shrub or small tree. It has economic use for its timber, and especially its berry-like cones are used for medical purposes and seasoning. Therefore, most of the seasonal studies are focused only on the berries, their potential medical use and optimal harvest time.

Norway spruce (*P. abies*) is an economically important tree species because of its timber, and it has also some traditional medical applications and nutritional value, especially for the annual shoots. Norway spruce is the only naturally growing species of the genus *Picea* in Finland.

### 3.2. Plant Sampling and Extraction

These populations were studied for their seasonal changes three times during a growing season over three years. Samplings were conducted in May/June, July and August from 2016 to 2018. The leaves were not sampled immediately after the bud burst but from nearly full-grown leaves to exclude the most drastic changes that take place in early spring. The first sampling for most of the species took place after their flowering phase, because part of the species flower at the early stage of the leaf growth (*A. platanoides*, *B. pubescens* and *P. padus*) or even before foliation (*S. phylicifolia*, *A. glutinosa* and *A. incana*). Also, the needles of *P. abies* were collected after the flowering. Only *J. communis*, *Q. robur* and *S. aucuparia* were sampled during the active flowering phase.

We studied five individual trees from the same population of each species to obtain a comprehensive picture of the variation in the population over the years. Only mature tree individuals were studied to exclude possible changes in polyphenol composition between juvenile and mature phases during the three-year study period. Unfortunately, only three individuals of *A. incana* were available in the last year. The sampling was conducted similarly as in Vanhakylä and Salminen [28]. Three branches from different sides of each tree were selected and cut, and 6 to 10 healthy and undamaged leaves per branch were collected to form a representative sample of the tree.

Samples were stored for a maximum of three hours in cool boxes with ice to slow down enzymatic activity before samples were frozen (−21 °C) in a laboratory freezer. After freezing, the samples were lyophilized for at least 24 h with a freeze-dryer. The dry leaves were ground thoroughly with a ball mill.

Water-soluble phenolics were extracted as previously [28] from 19.5–20.5 mg (by 0.1 mg accuracy) of plant powder using 2 × 1400 μL acetone/water (8/2, *v*/*v*) solvent. The three-hour extraction was conducted twice on a planary shaker, samples were centrifuged for 10 min (14,000 rpm) and the extracts were combined into new 2 mL tubes. Acetone was evaporated with an Eppendorf concentrator and samples were frozen and lyophilized. The freeze-dried extract was dissolved in 1 mL of ultra-pure water and filtered with 0.2 μm PTFE syringe filters before the analyses.

### 3.3. Chemical Analyses

Modified Folin–Ciocalteu assay [33] was used to measure total phenolic content before and after oxidation to detect the oxidatively active portion of phenolics in each sample as previously [28]. Each sample was analyzed in triplicate with a Multiscan Ascent microplate reader (Labsystems and Thermo Electron Corporation). An aliquot of 20 µL of the plant extract was pipetted on a 96-well plate in triplicate. Oxidation was initiated by adding 180 µL of sodium carbonate buffer (pH 10) and samples were incubated at 25 °C. After exactly 60 min, the oxidation was stopped by adding 100 µL of 0.6% formic acid. The total phenolic reactions were started by mixing 50 µL of each sample (oxidized and non-oxidized) with 50 µL of Folin-Ciocalteu reagent. After one minute of shaking, 100 µL of 20% sodium carbonate (*m*/*v*) was added and the plate was shaken. The absorbance was measured at 742 nm in 1 min intervals for 30 min and the maximal absorbance was used to calculate the total phenolic levels against a gallic acid standard curve (0 µg/mL, 10 µg/mL, 25 µg/mL and 100 µg/mL prepared in water).

The protein precipitation capacity (PPC) of the plant samples was measured with the radial diffusion assay (RDA) [72]. The RDA dishes were prepared by adding 10 mg of agarose and 1 g of BSA into 1000 mL of RDA buffer containing 10.6 mg of ascorbic acid, 2.85 mL of glacier acetic acid and 1000 mL of ultra-pure water as described in detail by Vanhakylä and Salminen [28]. Then 10 mL of the solution was pipetted into Petri dishes and left to set. Nine holes were punched into each dish. To prepare plant samples, 200 μL of plant extract (see the Section 3.2) was freeze-dried and dissolved into 100 μL of water. Pentagalloyl glucose/oenothein B (1/1, *v*/*v*) was used as a standard in concentrations of 1, 2, 3, 4, and 5 mg/mL, dissolved in 30% EtOH; 3 × 24 μL of each sample and standard was pipetted into the punched holes and let to diffuse into the gel. The dishes were incubated at 30 °C for three days. The precipitation ring area was measured with ImageJ [73] software and compared to PGG/OeB standard to solve the concentration of protein precipitative phenolics.

The polyphenol composition of the extracts was analyzed with ultrahigh-performance liquid chromatography coupled with a diode array detector and triple quadrupole mass spectrometer (UHPLC-DAD-QqQ-MS, Waters Corporation, Milford, MA, USA). Details of the chromatographic and mass spectrometric parameters were shown in Vanhakylä and Salminen, [28]. Polyphenol subgroups were quantified using the group-specific MRM methods for galloyl and HHDP derivatives, quinic acid derivatives, kaempferol, quercetin and myricetin derivatives, procyanidins and prodelphinidins [29,30]. The group-specific methods first fragment the UHPLC-separated polyphenols in the ion source using cone voltages separately optimized for each polyphenol group. Then the specific precursor ions representing the selected functional units of polyphenols are selected in the first quadrupole for fragmentation in the collision cell. The precursor-specific product ions are first accumulated by optimized collision energies and then selected by the last quadrupole for detection and creation of the group-specific fingerprints (Figure 2). External standards were used to transform the integrated group-specific fingerprints result into mg/g dry weight in each sample and a catechin solution (5 µg/mL) was injected into the system before and after every ten samples to correct for any changes in the MS performance. The method and its use are described in detail in Engström et al. [29,30] and in Malisch et al. [74].

### 3.4. Mass Spectrometric Fingerprint Mapping

To map the qualitative and quantitative results of the eight polyphenol groups and the two bioactivities with different variability scales on the same comprehensible chart, the data were first normalized between zero and 1.0 using a selected maximal value for each polyphenol and activity group. Maximal values were determined accordingly to Natural Chemistry Research Group’s plant library (<10,000 samples, <3500 species, Vanhakylä, Salminen et al. unpublished). This allows us to compare any data set to the occurrence of the selected polyphenol groups overall in the plant kingdom. To highlight and balance the contrast at lower concentrations, the maximal level was set to cover 95% of the samples, equalizing 5% of the highest values to the maximal level of 1.0. The highest 5% of the data was scattered on a large concentration scale and if the absolutely maximal concentrations were given the value 1.0, most of the samples would have been grouped at the low end of the concentration axis.

The maximal values (mg/g dry weight) for normalization were set as follows; OX: 40.0; PPC: 70.0; HT: 85.0; PA: 85.0; FL: 10.0; QA: 13.0; GA: 25.0; HHDP: 75.0; PC: 75.0; PD 45.0; KA: 6.0; QU: 8.5 and MY: 6.0. The initial concentrations at the normalized points and detected maximal values of each polyphenol and activity group are listed in Supplementary Table S12. The initial concentrations of each subgroup of flavonol derivatives at the normalized scale are listed in Table S13.

The quantification limit was set to 0.1 mg/g with flavonol glycosides, hydrolyzable tannins and quinic acid derivatives and to 1.0 mg/g with proanthocyanidins. These polyphenol groups were detected also below these concentrations, but their MRM trace intensities were at the 10^3^ or low 10^4^ levels and thus less reliable. The higher quantification limit of PAs was due to their characteristic way to form oligomer and polymer humps in chromatograms, making the integrated areas broader and thus the detection limits higher than with the other compound types producing defined sharp peaks. All in all, our aim was to report only true polyphenol values for each species and these quantification limits worked well for this aim as well.

### 3.5. Statistical Analyses

Statistical analyses were performed with SigmaPlot for Windows (Version 15, 15.0.0.13). The differences in the mean values of quantitative results of each polyphenol group between individual trees, years and sampling times were analyzed using a three-way analysis of variance (Three-way ANOVA). Pairwise multiple comparison procedures were performed with the Holm–Sidak method when significant differences were detected. The significance level was set to *p* = 0.05. The results of the statistical analyses are listed in Tables S14–S22.

## 4. Conclusions

Seasonal variation in the levels of plant defenses can have consequences on the plants’ ability to tolerate biotic and abiotic stress factors that have their peaks at different stages of the growing season. Herbivory levels may also change on a yearly basis and the gradually building insect outbreaks can cause delayed induction of plant defenses in the plant tissue of the coming year or years. In this study, we followed seasonal variation in the contents of major polyphenol groups in tree species and on purpose omitted sampling plant tissue during the most drastic times of seasonal change, i.e., bud break and early spring or summer [56,75]. Rapid changes during spring in tissue mass only are known to affect plant defense levels a lot by the tissue dilution effect [76]. Here, we wanted to focus on defense-level changes in plant tissue that have practically reached a mature size or leaf/needle area, even if the leaf or needle mass would still continue to rise a bit. This way most of the changes would correspond to truly decreasing, increasing, or stable levels of polyphenol synthesis in the studied plant tissue [8].

By monitoring the leaves of eight deciduous trees and needles of two conifers during three growing seasons, we were able to record both species-specific and compound group-specific changes during these seasons and years in the major polyphenol groups and related bioactivities. However, since these changes were quantitative rather than qualitative they did not dramatically change the way individual species looked in their 2D fingerprint maps. In fact, by picking any of the 18 fingerprint maps of a species showing either the major polyphenol groups and activities (Figure 1) or detailed flavonol patterns (Figure 2), it was possible to conclude which species these patterns belonged to. In addition, these 2D fingerprint maps nicely illustrated the main findings related to within-species variation, seasonal variation and variation between the years.

Looking at Figure 1 alone it is clear that the largest seasonal variation was linked to *B. pubescens*, *Q. robur* and *A. glutinosa*. All these species produced ellagitannins that showed repeatable declining trends during all growing seasons, excluding two of the *A. glutinosa* individuals that showed unusual plasticity in their hydrolyzable tannin production. The fourth species producing HTs was *A. platanoides* with simple gallic acid derivatives and gallotannins that did not show a clear declining pattern compared to ETs with the other three HT-producing species. The other major group of plant tannins, i.e., proanthocyanidins was found in all species with variable PC to PD compositions and seasonal variations. The main seasonal pattern for PAs was a clear increase (*S. phylicifolia*, *Q. robur* and *A. platanoides*) or an increase accompanied by stable levels during another year (*J. communis*, *P. padus*, *B. pubescens*, *A. glutinosa*, *A. incana*). Only for two species (*P. abies*, *S.aucuparia*) these two PA patterns were accompanied by slightly declining levels during one of the years. In general, these patterns confirmed the overall declining levels of ETs and increasing levels of PAs and their average size, with some variation across the species and years.

The flavonol derivatives showed variable KA/QU/MY ratios between the species that caused distinct differences in their fingerprint maps as well (Figure 2). These polyphenols had clearly declining levels in both of the conifers, and in general they decreased also in *Q. robur*, *S. phylicifolia*, *P. padus* and *A. glutinosa*, while in *A. incana* and *S. aucuparia* they remained stable and increased only in *A. platanoides* mainly because of its QU derivatives. Seasonal patterns of QA derivatives were similarly variable to FL derivatives, showing clearly decreasing patterns in both of the conifers, decreasing patterns in *S. aucuparia*, *P. padus*, *B. pubescens* and *A. platanoides*, and both increasing and decreasing patterns depending on the year in *A. incana*, *A. glutinosa* and *Q. robur*, and mainly increasing levels in *S. phylicifolia*.

For most of the species, the seasonal variation in major polyphenol groups was enough to explain corresponding changes in species oxidative activity and protein precipitation capacity. However, for some of the species we needed to look at the major individual polyphenols that were not detected by the automated group-specific MS/MS tools, to find explanations for the seasonal bioactivity patterns. Luckily many of these major polyphenols could be detected and characterized by their UV and full scan mass spectra and retention times that were recorded with UHPLC-DAD-MS together with the group-specific MS/MS fingerprints (e.g., [18]). This showed us how the inability of the automated MS/MS fingerprinting tool to detect all major polyphenols with oxidative activity and protein precipitation capacity could actually be a benefit; the lack of explanation for the seasonal variation of bioactivities by the detected compound groups could help us find even new types of active polyphenols in the plant kingdom, given that they can be detected by the more conventional DAD and full scan MS detection. Such findings are important in bigger plant screening programs as well so that all possible new types of polyphenols with these two (or other) bioactivities could be first detected and then characterized.

All in all, this study showed that the 2D fingerprint maps produced by the UHPLC-MS/MS tool of Engström et al. [29,30] can be used to effectively differentiate species from each other. These maps were also efficient in revealing the main patterns of seasonal and other variation within each species and these variations were not so large that species differentiation would be endangered. In addition, these maps and their normalized quantitation automatically linked the detected polyphenol and bioactivity levels to the bigger picture found in thousands of species collected from five continents. We are thus confident that this set of tools is suitable also for fingerprinting and monitoring major changes in plant polyphenols in changing climates, environments and herbivore pressures as typical to current challenges related to, e.g., climate change and biodiversity loss. For instance, with these tools, our study found unusual and non-uniform yearly patterns with *A. glutinosa* ellagitannins, indicating presumable induction of ET production in some black alder individuals especially in 2017 (Figure 1D). We did not measure herbivory levels in the study sites but alders, in general, have been recently under heavy pressure by the alder leaf beetle (*Agelastica alni*) in Southwestern Finland. It would be thus interesting to see for all the species how the fingerprint maps or some of their specific parts such as myricetin derivatives in *S. phylicifolia* (Figure 1F and Figure 2F) would change as a function of increasing or decreasing biotic or abiotic stress. For most, if not all the species, the current study was able to record the baseline levels against which all the future events could be compared at least with the same plant populations included here. For other populations or forest plots, it would be intriguing to see how their baseline levels compare to the levels measured here during the nine sampling times.

## Data Availability

The data presented in this study is available on request from the corresponding author.

## Supplementary Materials

The following supporting information can be downloaded at: https://www.mdpi.com/article/10.3390/molecules28166093/s1, Figures S1–S10: Examples of UHPLC-UV and group-specific UHPLC-MS/MS fingerprints of each species. (A) UV traces at 280 nm, (B) galloyl derivative fingerprint, (C) hexahydroxydiphenoyl derivative fingerprint, (D) procyanidin polymer fingerprint, (E) prodelphinidin polymer fingerprint, (F) quinic acid derivative fingerprint (Except the peak at 0.8 min, which is a free quinic acid found in plants, i.e., it is not a polyphenol), (G) kaempferol derivative fingerprint, (H) quercetin derivative fingerprint and (I) myricetin derivative fingerprint. The maximal values of the y-axes of chromatograms B–I are set according to the maximal values detected within the ten studied species.; Tables S1–S10: Initial concentration values of each tree populations. Values are averages of the five individual trees ± standard error of the mean; Figures S11A–S20A: The seasonal and yearly changes of hydrolyzable tannins (HT), galloyl and hexahydroxydiphenoyl derivatives (G and HHDP) and quinic acid derivatives (QA) of the individual trees of each species; Figures S11B–S20B: The seasonal and yearly changes of proanthocyanidins (PA), procyanidins (PC) and prodelphinidins (PD) of the individual trees of each species; Figures S11C–S20C: The seasonal and yearly changes of flavonoid glycosides (FL), kaempferol glycosides (KA), quercetin glycosides (QU) and myricetin glycosides (MY) of the individual trees of each species; Table S11: Coordinates of the plant populations and collection dates of the 1st, 2nd, and 3rd collection; Table S12: The initial concentration values (mg/g) of each polyphenol group and bioactivity at normalized scale. Table S13: The initial concentration values (mg/g) of each flavonol group and glycosylation pattern at normalized scale.; Tables S14–S22: The results of statistical analyses of each species. The differences in the mean values of quantitative results of each polyphenol group between individual trees, years and sampling times were analyzed using a three-way analysis of variance (Three-way ANOVA). Pairwise multiple comparison procedures were performed with Holm–Sidak method when significant differences were detected. The significance level was set to *p* = 0.05.

## References

[1] Agrawal A.A., Hastings A.P., Johnson M.T.J., Maron J.L., Salminen J.-P. (2012). Insect Herbivores Drive Real-Time Ecological and Evolutionary Change in Plant Populations. Science.

[2] McArt S.H., Halitschke R., Salminen J.-P., Thaler J.S. (2013). Leaf Herbivory Increases Plant Fitness via Induced Resistance to Seed Predators. Ecology.

[3] Lemoine N.P., Doublet D., Salminen J.-P., Burkepile D.E., Parker J.D. (2017). Responses of Plant Phenology, Growth, Defense, and Reproduction to Interactive Effects of Warming and Insect Herbivory. Ecology.

[4] Johnson M.T.J., Ives A.R., Ahern J., Salminen J.-P. (2014). Macroevolution of Plant Defenses against Herbivores in the Evening Primroses. New Phytol..

[5] Halsch C.A., Shapiro A.M., Fordyce J.A., Nice C.C., Thorne J.H., Waetjen D.P., Forister M.L. (2021). Insects and Recent Climate Change. Proc. Natl. Acad. Sci. USA.

[6] Outhwaite C.L., McCann P., Newbold T. (2022). Agriculture and Climate Change Are Reshaping Insect Biodiversity Worldwide. Nature.

[7] Seifert C.L., Jorge L.R., Volf M., Wagner D.L., Lamarre G.P.A., Miller S.E., Gonzalez-Akre E., Anderson-Teixeira K.J., Novotný V. (2021). Seasonality Affects Specialisation of a Temperate Forest Herbivore Community. Oikos.

[8] Salminen J.-P., Ossipov V., Haukioja E., Pihlaja K. (2001). Seasonal Variation in the Content of Hydrolysable Tannins in Leaves of *Betula pubescens*. Phytochemistry.

[9] Salminen J.-P., Roslin T., Karonen M., Sinkkonen J., Pihlaja K., Pulkkinen P. (2004). Seasonal Variation in the Content of Hydrolyzable Tannins, Flavonoid Glycosides, and Proanthocyanidins in Oak Leaves. J. Chem. Ecol..

[10] Valkama E., Salminen J.-P., Koricheva J., Kalevi P. (2004). Changes in Leaf Trichomes and Epicuticular Flavonoids during Leaf Development in Three Birch Taxa. Ann. Bot..

[11] Zidorn C. (2018). Seasonal Variation of Natural Products in European Trees. Phytochem. Rev..

[12] Eberhardt T.L., Young R.A. (1994). Conifer Seed Cone Proanthocyanidin Polymers: Characterization by 13C NMR Spectroscopy and Determination of Antifungal Activities. J. Agric. Food Chem..

[13] Virjamo V., Julkunen-Tiitto R. (2014). Shoot Development of Norway Spruce (*Picea abies*) Involves Changes in Piperidine Alkaloids and Condensed Tannins. Trees.

[14] Barbehenn R.V., Jones C.P., Hagerman A.E., Karonen M., Salminen J.-P. (2006). Ellagitannins Have Greater Oxidative Activities than Condensed Tannins and Galloyl Glucoses at High pH: Potential Impact on Caterpillars. J. Chem. Ecol..

[15] Moilanen J., Salminen J.-P. (2008). Ecologically Neglected Tannins and Their Biologically Relevant Activity: Chemical Structures of Plant Ellagitannins Reveal Their in vitro Oxidative Activity at High pH. Chemoecology.

[16] Moilanen J., Karonen M., Tähtinen P., Jacquet R., Quideau S., Salminen J.-P. (2016). Biological Activity of Ellagitannins: Effects as Anti-Oxidants, pro-Oxidants and Metal Chelators. Phytochemistry.

[17] Kim J., Pälijärvi M., Karonen M., Salminen J.-P. (2018). Oxidatively Active Plant Phenolics Detected by UHPLC-DAD-MS after Enzymatic and Alkaline Oxidation. J. Chem. Ecol..

[18] Kim J., Pälijärvi M., Karonen M., Salminen J.-P. (2020). Distribution of Enzymatic and Alkaline Oxidative Activities of Phenolic Compounds in Plants. Phytochemistry.

[19] Dobreva M.A., Green R.J., Mueller-Harvey I., Salminen J.-P., Howlin B.J., Frazier R.A. (2014). Size and Molecular Flexibility Affect the Binding of Ellagitannins to Bovine Serum Albumin. J. Agric. Food Chem..

[20] Engström M.T., Arvola J., Nenonen S., Virtanen V.T.J., Leppä M.M., Tähtinen P., Salminen J.-P. (2019). Structural Features of Hydrolyzable Tannins Determine Their Ability to Form Insoluble Complexes with Bovine Serum Albumin. J. Agric. Food Chem..

[21] Karonen M., Oraviita M., Mueller-Harvey I., Salminen J.-P., Green R.J. (2019). Ellagitannins with Glucopyranose Cores Have Higher Affinities to Proteins than Acyclic Ellagitannins by Isothermal Titration Calorimetry. J. Agric. Food Chem..

[22] Leppä M.M., Laitila J.E., Salminen J.-P. (2020). Distribution of Protein Precipitation Capacity within Variable Proanthocyanidin Fingerprints. Molecules.

[23] Engström M.T., Virtanen V., Salminen J.-P. (2022). Influence of the Hydrolyzable Tannin Structure on the Characteristics of Insoluble Hydrolyzable Tannin-Protein Complexes. J. Agric. Food Chem..

[24] Engström M.T., Sun X., Suber M.P., Li M., Salminen J.-P., Hagerman A.E. (2016). The Oxidative Activity of Ellagitannins Dictates Their Tendency to Form Highly Stabilized Complexes with Bovine Serum Albumin at Increased pH. J. Agric. Food Chem..

[25] Baert N., Pellikaan W.F., Karonen M., Salminen J.-P. (2016). A Study of the Structure-Activity Relationship of Oligomeric Ellagitannins on Ruminal Fermentation in vitro. J. Dairy Sci..

[26] Engström M.T., Karonen M., Ahern J.R., Baert N., Payré B., Hoste H., Salminen J.-P. (2016). Chemical Structures of Plant Hydrolyzable Tannins Reveal Their in Vitro Activity against Egg Hatching and Motility of *Haemonchus contortus* Nematodes. J. Agric. Food Chem..

[27] Karonen M., Ahern J.R., Legroux L., Suvanto J., Engström M.T., Sinkkonen J., Salminen J.-P., Hoste H. (2020). Ellagitannins Inhibit the Exsheathment of *Haemonchus contortus* and *Trichostrongylus colubriformis* Larvae: The Efficiency Increases Together with the Molecular Size. J. Agric. Food Chem..

[28] Vanhakylä S., Salminen J.-P. (2023). Mass Spectrometric Fingerprint Mapping Reveals Species-Specific Differences in Plant Polyphenols and Related Bioactivities. Molecules.

[29] Engström M.T., Pälijärvi M., Fryganas C., Grabber J.H., Mueller-Harvey I., Salminen J.-P. (2014). Rapid Qualitative and Quantitative Analyses of Proanthocyanidin Oligomers and Polymers by UPLC-MS/MS. J. Agric. Food Chem..

[30] Engström M.T., Pälijärvi M., Salminen J.-P. (2015). Rapid Fingerprint Analysis of Plant Extracts for Ellagitannins, Gallic Acid, and Quinic Acid Derivatives and Quercetin-, Kaempferol- and Myricetin-Based Flavonol Glycosides by UPLC-QqQ-MS/MS. J. Agric. Food Chem..

[31] Salminen J.-P. (2018). Two-Dimensional Tannin Fingerprints by Liquid Chromatography Tandem Mass Spectrometry Offer a New Dimension to Plant Tannin Analyses and Help to Visualize the Tannin Diversity in Plants. J. Agric. Food Chem..

[32] Ossipov V., Salminen J.-P., Ossipova S., Haukioja E., Pihlaja K. (2003). Gallic Acid and Hydrolysable Tannins Are Formed in Birch Leaves from an Intermediate Compound of the Shikimate Pathway. Biochem. Syst. Ecol..

[33] Salminen J.-P., Karonen M. (2011). Chemical Ecology of Tannins and Other Phenolics: We Need a Change in Approach. Funct. Ecol..

[34] Riipi M., Haukioja E., Lempa K., Ossipov V., Ossipova S., Pihlaja K. (2004). Ranking of Individual Mountain Birch Trees in Terms of Leaf Chemistry: Seasonal and Annual Variation. Chemoecology.

[35] Salminen J.-P., Ossipov V., Pihlaja K. (2002). Distribution of Hydrolysable Tannins in the Foliage of Finnish Birch Species. Z. Naturforsch. C J. Biosci..

[36] Riipi M., Ossipov V., Lempa K., Haukioja E., Koricheva J., Ossipova S., Pihlaja K. (2002). Seasonal Changes in Birch Leaf Chemistry: Are There Trade-Offs between Leaf Growth and Accumulation of Phenolics?. Oecologia.

[37] Ossipova S.V., Ossipov V., Haukioja E., Loponen J., Pihlaja K. (2001). Proanthocyanidins of Mountain Birch Leaves: Quantification and Properties. Phytochem. Anal..

[38] James A.M., Ma D., Mellway R., Gesell A., Yoshida K., Walker V., Tran L., Stewart D., Reichelt M., Suvanto J. (2017). Poplar MYB115 and MYB134 Transcription Factors Regulate Proanthocyanidin Synthesis and Structure. Plant Physiol..

[39] Raal A., Boikova T., Püssa T. (2015). Content and Dynamics of Polyphenols in *Betula* spp. Leaves Naturally Growing in Estonia. Rec. Nat. Prod..

[40] Visakorpi K., Riutta T., Malhi Y., Salminen J.-P., Salinas N., Gripenberg S. (2020). Changes in Oak (*Quercus robur*) Photosynthesis after Winter Moth (*Operophtera brumata*) Herbivory Are Not Explained by Changes in Chemical or Structural Leaf Traits. PLoS ONE.

[41] Tálos-Nebehaj E., Hofmann T., Albert L. (2017). Seasonal Changes of Natural Antioxidant Content in the Leaves of Hungarian Forest Trees. Ind. Crops Prod..

[42] Veselá H., Lhotáková Z., Albrechtová J., Frouz J. (2021). Seasonal Changes in Tree Foliage and Litterfall Composition at Reclaimed and Unreclaimed Post-Mining Sites. Ecol. Eng..

[43] Moilanen J., Koskinen P., Salminen J.-P. (2015). Distribution and Content of Ellagitannins in Finnish Plant Species. Phytochemistry.

[44] Kotilainen T., Tegelberg R., Julkunen-Tiitto R., Lindfors A., O’Hara R.B., Aphalo P.J. (2010). Seasonal Fluctuations in Leaf Phenolic Composition under UV Manipulations Reflect Contrasting Strategies of Alder and Birch Trees. Physiol. Plant.

[45] Tegelberg R., Veteli T., Aphalo P.J., Julkunen-Tiitto R. (2003). Clonal Differences in Growth and Phenolics of Willows Exposed to Elevated Ultraviolet-B Radiation. Basic Appl. Ecol..

[46] Nissinen K., Virjamo V., Mehtätalo L., Lavola A., Valtonen A., Nybakken L., Julkunen-Tiitto R. (2018). A Seven-Year Study of Phenolic Concentrations of the Dioecious *Salix myrsinifolia*. J. Chem. Ecol..

[47] Obeso J.R. (2002). The Costs of Reproduction in Plants. New Phytol..

[48] Olszewska M. (2008). Separation of Quercetin, Sexangularetin, Kaempferol and Isorhamnetin for Simultaneous HPLC Determination of Flavonoid Aglycones in Inflorescences, Leaves and Fruits of Three *Sorbus* Species. J. Pharm. Biomed. Anal..

[49] Turumtay H., Midilli A., Turumtay E.A., Demir A., Selvi E.K., Budak E.E., Er H., Kocaimamoglu F., Baykal H., Belduz A.O. (2017). Gram (−) Microorganisms DNA Polymerase Inhibition, Antibacterial and Chemical Properties of Fruit and Leaf Extracts of *Sorbus acuparia* and *Sorbus caucasica* var. *yaltirikii*. Biomed. Chromatogr..

[50] Olszewska M.A. (2011). Variation in the Phenolic Content and in Vitro Antioxidant Activity of *Sorbus aucuparia* Leaf Extracts during Vegetation. Acta Pol. Pharm..

[51] Ravetto Enri S., Probo M., Renna M., Caro E., Lussiana C., Battaglini L.M., Lombardi G., Lonati M. (2020). Temporal Variations in Leaf Traits, Chemical Composition and in vitro True Digestibility of Four Temperate Fodder Tree Species. Anim. Prod. Sci..

[52] Olszewska M.A., Kwapisz A. (2011). Metabolite Profiling and Antioxidant Activity of *Prunus padus* L. Flowers and Leaves. Nat. Prod. Res..

[53] Mattila H., Valev D., Havurinne V., Khorobrykh S., Virtanen O., Antinluoma M., Mishra K.B., Tyystjärvi E. (2018). Degradation of Chlorophyll and Synthesis of Flavonols during Autumn Senescence-the Story Told by Individual Leaves. AoB Plants.

[54] Artemkina N.A., Orlova M.A., Lukina N.V. (2016). Chemical Composition of *Juniperus sibirica* Needles (Cupressaceae) in the Forest-Tundra Ecotone, the Khibiny Mountains. Russ. J. Ecol..

[55] Naumann H.D., Stewart W.C., Whitney T.R. (2018). The Effect of Maturity on Concentration and Biological Activity of Protein Precipitating Polyphenolics in Ground Juniper Is Dependent upon Species. Anim. Feed Sci. Technol..

[56] Slimestad R., Hostettmann K. (1996). Characterisation of Phenolic Constituents from Juvenile and Mature Needles of Norway Spruce by Means of High Performance Liquid Chromatography-Mass Spectrometry. Phytochem. Anal..

[57] Martz F.-O., Peltola R., Fontanay S.P., Raphae R., Duval R.E., Julkunen-Tiitto R., Stark S. (2009). Effect of Latitude and Altitude on the Terpenoid and Soluble Phenolic Composition of Juniper (*Juniperus communis*) Needles and Evaluation of Their Antibacterial Activity in the Boreal Zone. J. Agric. Food Chem..

[58] Slimestad R. (1998). Amount of Flavonols and Stilbenes during Needle Development of *Picea abies*; Variations between Provenances. Biochem. Syst. Ecol..

[59] Ganthaler A., Stöggl W., Kranner I., Mayr S. (2017). Foliar Phenolic Compounds in Norway Spruce with Varying Susceptibility to *Chrysomyxa rhododendri*: Analyses of Seasonal and Infection-Induced Accumulation Patterns. Front. Plant Sci..

[60] Christenhusz M., Reveal J.L., Farjon A., Gardner M., Mill R., Chase M. (2011). A new classification and linear sequence of extant gymnosperms. Phytotaxa.

[61] The Angiosperm Phylogeny Group (2016). An update of the Angiosperm Phylogeny Group classification for the orders and families of flowering plants: APG IV. Bot. J. Linn. Soc..

[62] Bi W., Gao Y., Shen J., He C., Liu H., Peng Y., Zhang C., Xiao P. (2016). Traditional Uses, Phytochemistry, and Pharmacology of the Genus *Acer* (Maple): A Review. J. Ethnopharmacol..

[63] Arshanitsa A., Ponomarenko J., Lauberte L., Jurkjane V., Pals M., Akishin Y., Lauberts M., Jashina L., Bikovens O., Telysheva G. (2022). Advantages of MW-Assisted Water Extraction, Combined with Steam Explosion, of Black Alder Bark in Terms of Isolating Valuable Compounds and Energy Efficiency. Ind. Crops Prod..

[64] Lauberts M., Pals M. (2021). Antioxidant Activity of Different Extracts from Black Alder (*Alnus glutinosa*) Bark with Greener Extraction Alternative. Plants.

[65] Nawirska-Olszańska A., Zaczyńska E., Czarny A., Kolniak-Ostek J. (2022). Chemical Characteristics of Ethanol and Water Extracts of Black Alder (*Alnus glutinosa* L.) Acorns and Their Antibacterial, Anti-Fungal and Antitumor Properties. Molecules.

[66] Mahdi J.G., Mahdi A.J., Bowen I.D. (2006). The Historical Analysis of Aspirin Discovery, Its Relation to the Willow Tree and Antiproliferative and Anticancer Potential. Cell Prolif..

[67] Piątczak E., Dybowska M., Płuciennik E., Kośla K., Kolniak-ostek J., Kalinowska-lis U. (2020). Identification and Accumulation of Phenolic Compounds in the Leaves and Bark of *Salix alba* (L.) and Their Biological Potential. Biomolecules.

[68] Rank N.E., Köpf A., Julkunen-Tiitto R., Tahvanainen J. (1998). Host Preference and Larval Performance of the Salicylate-Using Leaf Beetle *Phratora vitellinae*. Ecology.

[69] Ruuhola T., Nybakken L., Julkunen-Tiitto R. (2013). Sex-Related Differences of Two Ecologically Divergent *Salix* Species in the Responses of Enzyme Activities to Atmospheric CO_2_ Enrichment. Biol. Plant.

[70] Sile I., Videja M., Makrecka-Kuka M., Tirzite D., Pajuste K., Shubin K., Krizhanovska V., Grinberga S., Pugovics O., Dambrova M. (2021). Chemical Composition of *Prunus padus* L. Flower Extract and Its Anti-Inflammatory Activities in Primary Bone Marrow-Derived Macrophages. J. Ethnopharmacol..

[71] Donno D., Mellano M.G., De Biaggi M., Riondato I., Rakotoniaina E.N., Beccaro G.L. (2018). New Findings in *Prunus padus* L. Fruits as a Source of Natural Compounds: Characterization of Metabolite Profiles and Preliminary Evaluation of Antioxidant Activity. Molecules.

[72] Hagerman A.E. (1987). Radial Diffusion Method for Determining Tannin in Plant Extracts. J. Chem. Ecol..

[73] Abràmoff M.D., Magalhães P.J., Ram S.J. (2004). Image Processing with ImageJ. Biophotonics Int..

[74] Malisch C.S., Lüscher A., Baert N., Engström M.T., Studer B., Fryganas C., Suter D., Mueller-Harvey I., Salminen J.-P. (2015). Large Variability of Proanthocyanidin Content and Composition in Sainfoin (*Onobrychis viciifolia*). J. Agric. Food Chem..

[75] Boege K., Marquis R.J. (2005). Facing Herbivory as You Grow up: The Ontogeny of Resistance in Plants. Trends Ecol. Evol..

[76] Koricheva J. (1999). Interpreting Phenotypic Variation in Plant Allelochemistry: Problems with the Use of Concentrations. Oecologia.

