# Supplementary material for: Seasonal Variation in Plant Polyphenols and Related Bioactivities across Three Years in Ten Tree Species as Visualized by Mass Spectrometric Fingerprint Mapping
Source: Molecules. 2023 Aug 16;28(16):6093. doi: 10.3390/molecules28166093 (PMC10458088; doi:10.3390/molecules28166093)
Supplement: Supplementary file 1 [file molecules-28-06093-s001.zip › molecules-2454855-supplementary.pdf]

Supplementary material

Vanhakylä S. & Salminen J.-P. (2023) Seasonal Variation in Plant Polyphenols and Related Bioactivities Across Three Years in Ten Tree Species as Visualized by Mass Spectrometric Fingerprint Mapping

List of Abbreviations used in Supplementary Material

TP	total phenolics
HT	hydrolysable tannins
G	gallic acid derivatives
HHDP	hexahydroxydiphenoyl derivatives
PA	proanthocyanidins
PC	procyanidins
PD	prodelphinidins
mDP	mean degree of polymerization
FL	flavonol glycosides
KA	kaempferol glycosides
QU	quercetin glycosides
MY	myricetin glycosides
QA	quinic acid derivatives
OX	oxidative activity
PPC	protein precipitation capacity

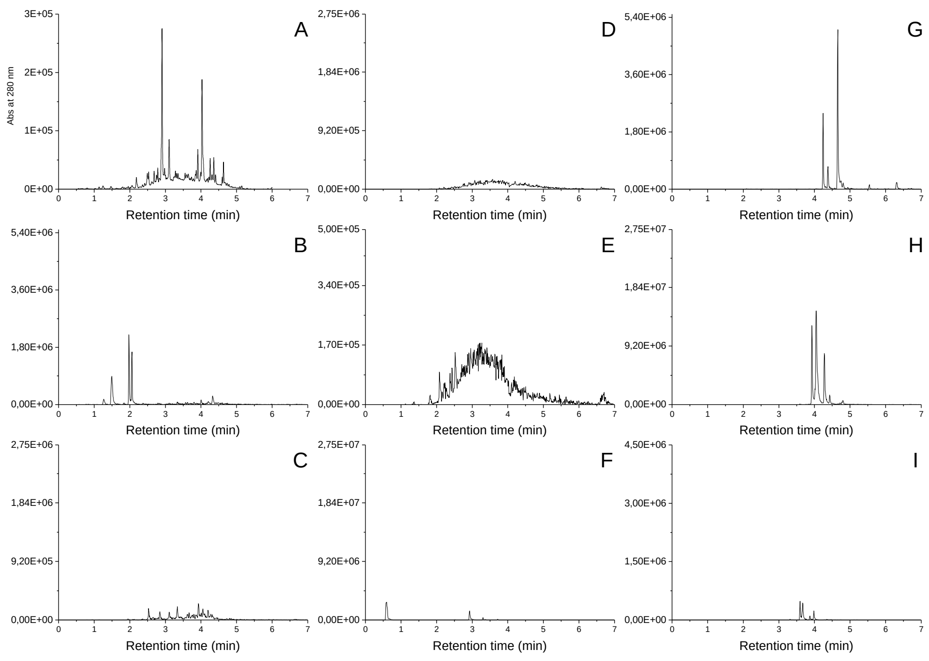

Figure S1. Examples of UHPLC-UV and group-specific UHPLC-MS/MS fingerprints of *Betula pubescens*. (A) UV traces at 280 nm, (B) galloyl derivative fingerprint, (C) hexahydroxydiphenoyl derivative fingerprint, (D) procyanidin polymer fingerprint, (E) prodelphinidin polymer fingerprint, (F) quinic acid derivative fingerprint (Except the peak at 0.8 min, which is a free quinic acid found in plants, i.e. it is not a polyphenol), (G) kaempferol derivative fingerprint, (H) quercetin derivative fingerprint and (I) myricetin derivative fingerprint. The maximal values of the y-axes of chromatograms B–I are set according to the maximal values detected within the ten studied species.

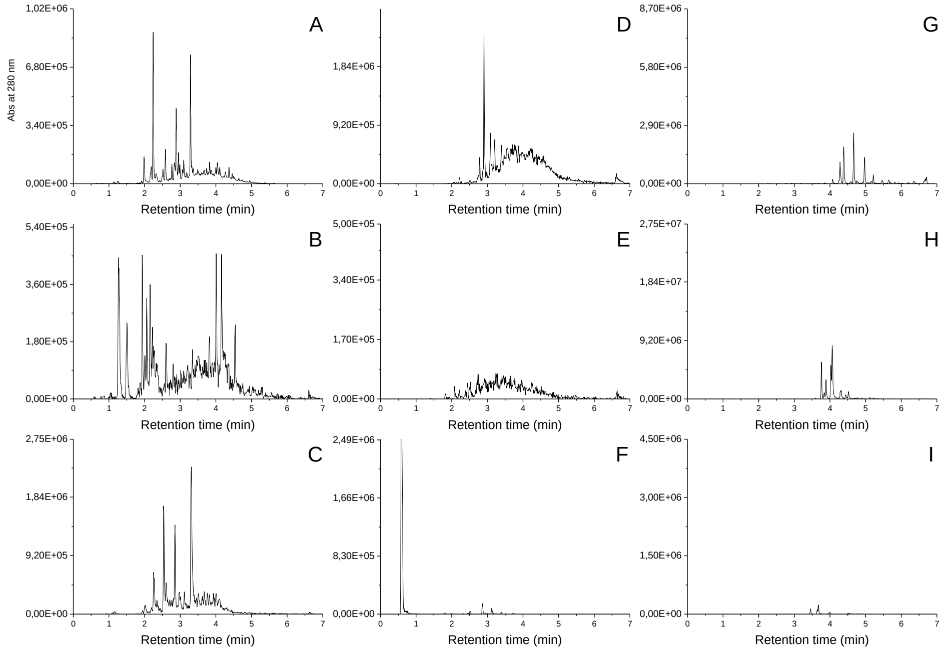

Figure S2. Examples of UHPLC-UV and group-specific UHPLC-MS/MS fingerprints of *Quercus robur*. (A) UV traces at 280 nm, (B) galloyl derivative fingerprint, (C) hexahydroxydiphenoyl derivative fingerprint, (D) procyanidin polymer fingerprint, (E) prodelphinidin polymer fingerprint, (F) quinic acid derivative fingerprint (Except the peak at 0.8 min, which is a free quinic acid found in plants, i.e. it is not a polyphenol), (G) kaempferol derivative fingerprint, (H) quercetin derivative fingerprint and (I) myricetin derivative fingerprint. The maximal values of the y-axes of chromatograms B–I are set according to the maximal values detected within the ten studied species.

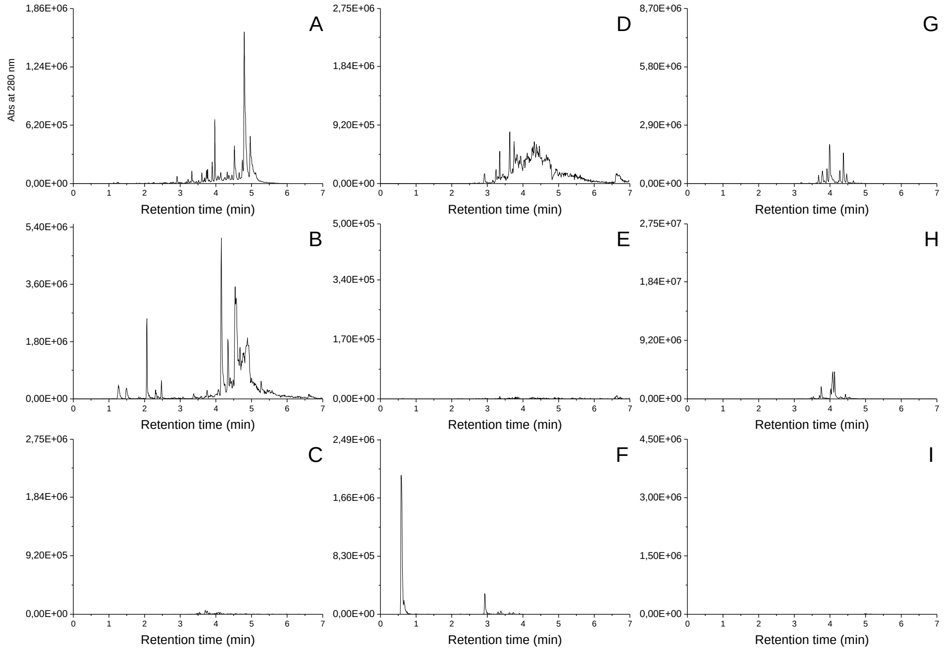

Figure S3. Examples of UHPLC-UV and group-specific UHPLC-MS/MS fingerprints of *Acer platanoides*. (A) UV traces at 280 nm, (B) galloyl derivative fingerprint, (C) hexahydroxydiphenoyl derivative fingerprint, (D) procyanidin polymer fingerprint, (E) prodelphinidin polymer fingerprint, (F) quinic acid derivative fingerprint (Except the peak at 0.8 min, which is a free quinic acid found in plants, i.e. it is not a polyphenol), (G) kaempferol derivative fingerprint, (H) quercetin derivative fingerprint and (I) myricetin derivative fingerprint. The maximal values of the y-axes of chromatograms B–I are set according to the maximal values detected within the ten studied species.

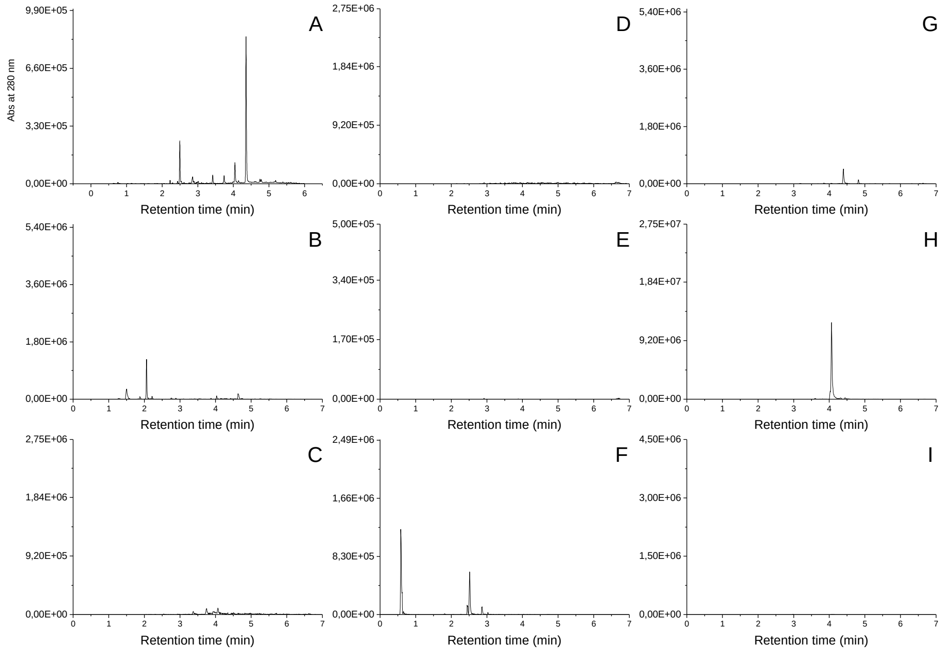

Figure S4. Examples of UHPLC-UV and group-specific UHPLC-MS/MS fingerprints of *Alnus glutinosa*. (A) UV traces at 280 nm, (B) galloyl derivative fingerprint, (C) hexahydroxydiphenoyl derivative fingerprint, (D) procyanidin polymer fingerprint, (E) prodelphinidin polymer fingerprint, (F) quinic acid derivative fingerprint (Except the peak at 0.8 min, which is a free quinic acid found in plants, i.e. it is not a polyphenol), (G) kaempferol derivative fingerprint, (H) quercetin derivative fingerprint and (I) myricetin derivative fingerprint. The maximal values of the y-axes of chromatograms B–I are set according to the maximal values detected within the ten studied species.

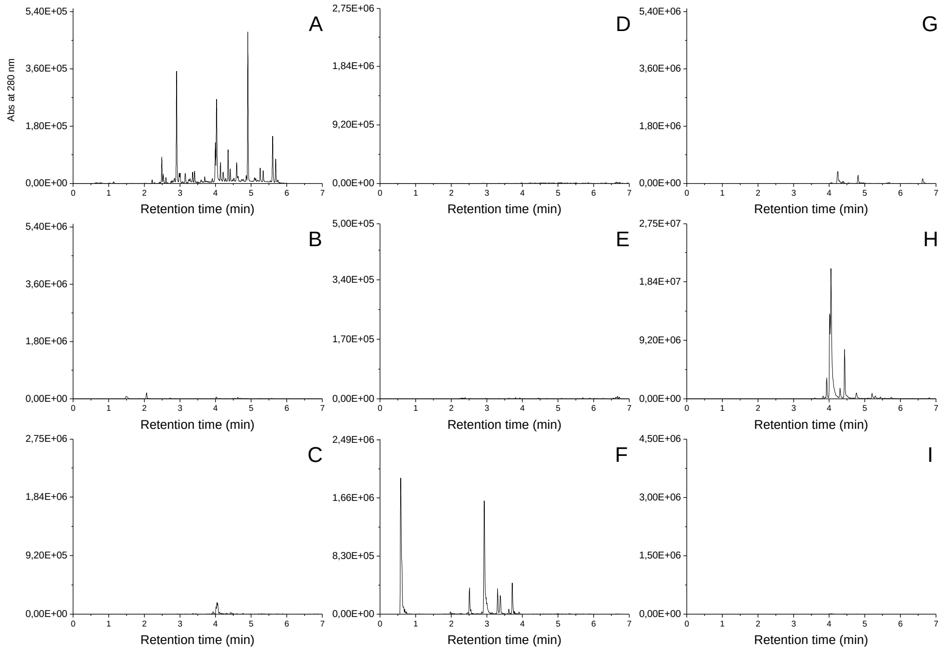

Figure S5. Examples of UHPLC-UV and group-specific UHPLC-MS/MS fingerprints of *Alnus incana*. (A) UV traces at 280 nm, (B) galloyl derivative fingerprint, (C) hexahydroxydiphenoyl derivative fingerprint, (D) procyanidin polymer fingerprint, (E) prodelphinidin polymer fingerprint, (F) quinic acid derivative fingerprint (Except the peak at 0.8 min, which is a free quinic acid found in plants, i.e. it is not a polyphenol), (G) kaempferol derivative fingerprint, (H) quercetin derivative fingerprint and (I) myricetin derivative fingerprint. The maximal values of the y-axes of chromatograms B–I are set according to the maximal values detected within the ten studied species.

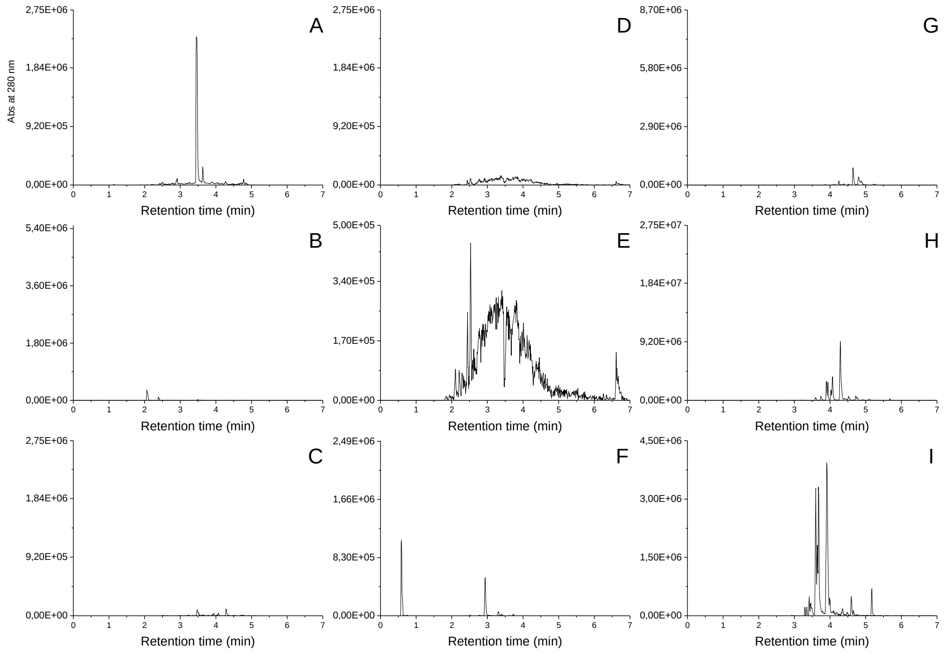

Figure S6. Examples of UHPLC-UV and group-specific UHPLC-MS/MS fingerprints of *Salix phylicifolia*. (A) UV traces at 280 nm, (B) galloyl derivative fingerprint, (C) hexahydroxydiphenoyl derivative fingerprint, (D) procyanidin polymer fingerprint, (E) prodelphinidin polymer fingerprint, (F) quinic acid derivative fingerprint (Except the peak at 0.8 min, which is a free quinic acid found in plants, i.e. it is not a polyphenol), (G) kaempferol derivative fingerprint, (H) quercetin derivative fingerprint and (I) myricetin derivative fingerprint. The maximal values of the y-axes of chromatograms B–I are set according to the maximal values detected within the ten studied species.

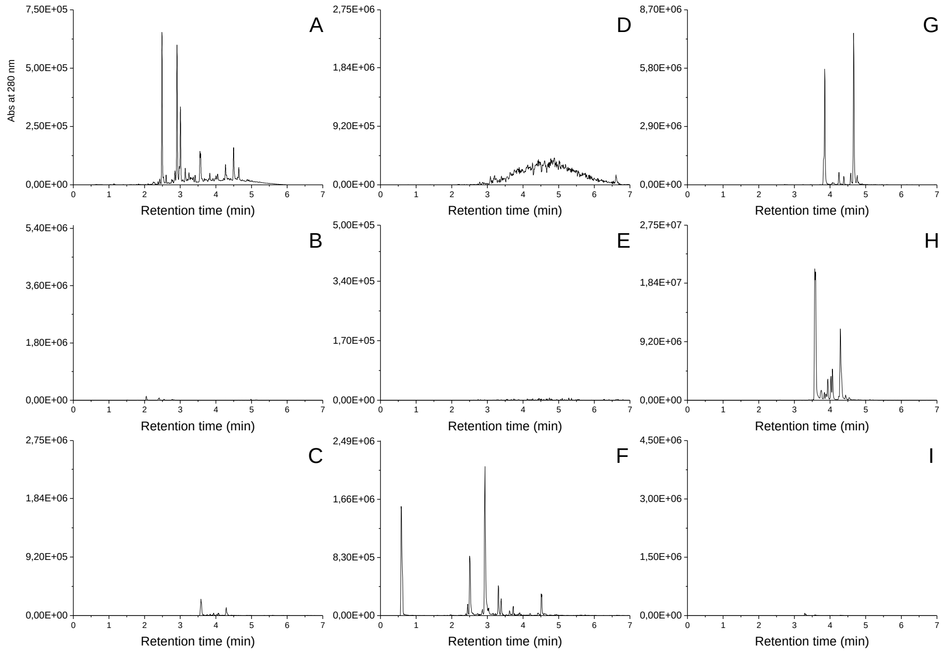

Figure S7. Examples of UHPLC-UV and group-specific UHPLC-MS/MS fingerprints of *Sorbus aucuparia*. (A) UV traces at 280 nm, (B) galloyl derivative fingerprint, (C) hexahydroxydiphenoyl derivative fingerprint, (D) procyanidin polymer fingerprint, (E) prodelphinidin polymer fingerprint, (F) quinic acid derivative fingerprint (Except the peak at 0.8 min, which is a free quinic acid found in plants, i.e. it is not a polyphenol), (G) kaempferol derivative fingerprint, (H) quercetin derivative fingerprint and (I) myricetin derivative fingerprint. The maximal values of the y-axes of chromatograms B–I are set according to the maximal values detected within the ten studied species.

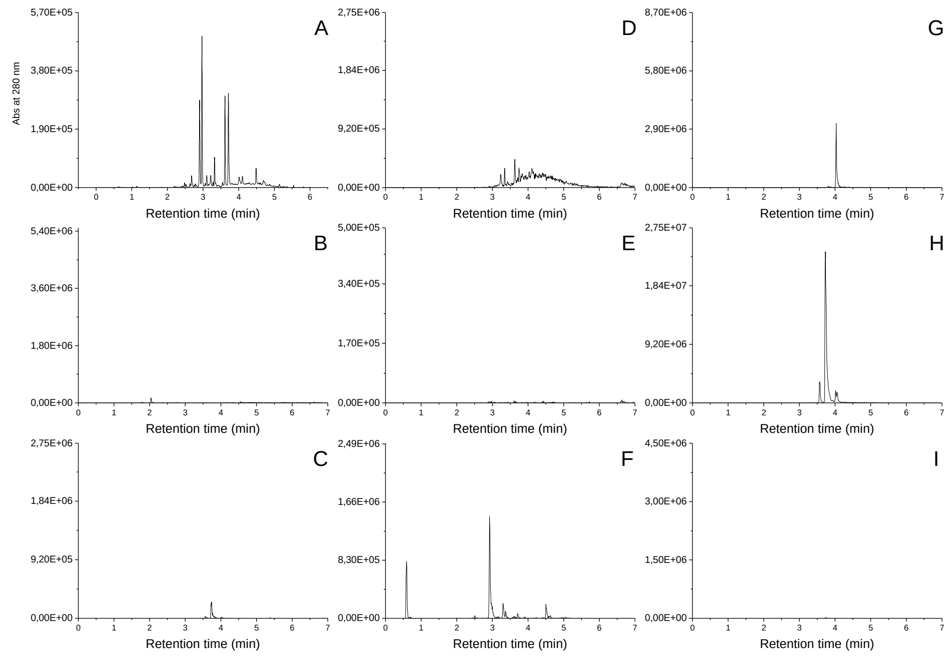

Figure S8. Examples of UHPLC-UV and group-specific UHPLC-MS/MS fingerprints of *Prunus padus*. (A) UV traces at 280 nm, (B) galloyl derivative fingerprint, (C) hexahydroxydiphenoyl derivative fingerprint, (D) procyanidin polymer fingerprint, (E) prodelphinidin polymer fingerprint, (F) quinic acid derivative fingerprint (Except the peak at 0.8 min, which is a free quinic acid found in plants, i.e. it is not a polyphenol), (G) kaempferol derivative fingerprint, (H) quercetin derivative fingerprint and (I) myricetin derivative fingerprint. The maximal values of the y-axes of chromatograms B–I are set according to the maximal values detected within the ten studied species.

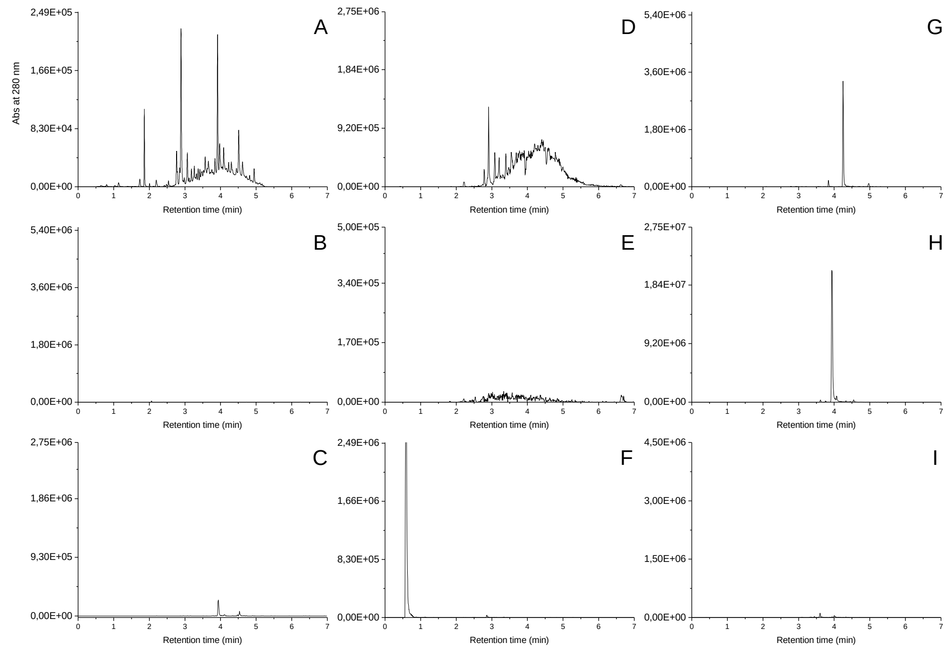

Figure S9. Examples of UHPLC-UV and group-specific UHPLC-MS/MS fingerprints of *Juniperus communis*. (A) UV traces at 280 nm, (B) galloyl derivative fingerprint, (C) hexahydroxydiphenoyl derivative fingerprint, (D) procyanidin polymer fingerprint, (E) prodelphinidin polymer fingerprint, (F) quinic acid derivative fingerprint (Except the peak at 0.8 min, which is a free quinic acid found in plants, i.e. it is not a polyphenol), (G) kaempferol derivative fingerprint, (H) quercetin derivative fingerprint and (I) myricetin derivative fingerprint. The maximal values of the y-axes of chromatograms B–I are set according to the maximal values detected within the ten studied species.

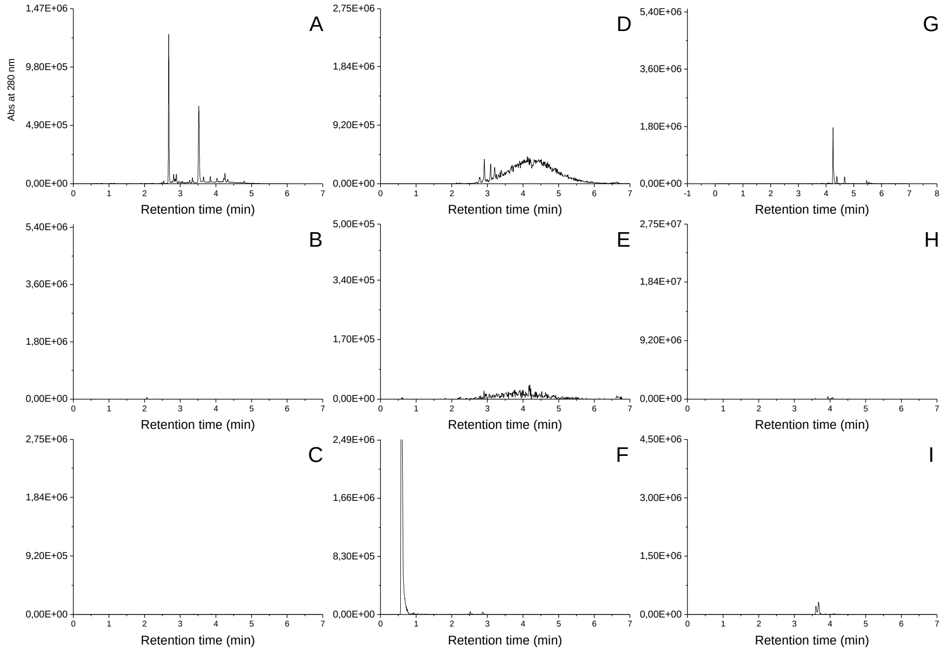

Figure S10. Examples of UHPLC-UV and group-specific UHPLC-MS/MS fingerprints of *Picea abies*. (A) UV traces at 280 nm, (B) galloyl derivative fingerprint, (C) hexahydroxydiphenoyl derivative fingerprint, (D) procyanidin polymer fingerprint, (E) prodelphinidin polymer fingerprint, (F) quinic acid derivative fingerprint (Except the peak at 0.8 min, which is a free quinic acid found in plants, i.e. it is not a polyphenol), (G) kaempferol derivative fingerprint, (H) quercetin derivative fingerprint and (I) myricetin derivative fingerprint. The maximal values of the y-axes of chromatograms B–I are set according to the maximal values detected within the ten studied species.

Table S1. Initial concentrations of the *Betula pubescens* population. Values are averages of the five individual trees ± standard error of the mean.

Betula pubescens		2016			2017			2018		
		June	July	August	June	July	August	June	July	August
Polyphenols	**TP**	60.2 ± 1.9	52.7 ± 2.8	51.8 ± 3.3	61.8 ± 2.4	59.3 ± 1.8	61.5 ± 2.3	53.4 ± 1.8	53.6 ± 2.5	51 ± 2.3
	**HT**	23.4 ± 2	10.1 ± 2.2	8.0 ± 2.0	15.5 ± 3.3	12.7 ± 2.3	9.6 ± 1.7	13.2 ± 1.1	8.6 ± 1.4	6.6 ± 1.0
	G	5.2 ± 0.6	2.9 ± 0.4	2.3 ± 0.3	3.8 ± 0.8	3.2 ± 0.5	3.0 ± 0.4	3.0 ± 0.4	2.2 ± 0.3	1.8 ± 0.3
	HHDP	18.2 ± 1.5	7.2 ± 1.8	5.7 ± 1.6	11.7 ± 2.5	9.6 ± 1.9	6.6 ± 1.3	10.3 ± 0.8	6.4 ± 1.1	4.8 ± 0.8
	**PA**	24.1 ± 1.0	27.7 ± 1.9	31.1 ± 1.9	21.7 ± 3.6	25.7 ± 2.3	26.1 ± 2.0	20.0 ± 1.4	24.0 ± 2.5	26.4 ± 1.8
	PC	8.4 ± 0.5	9.9 ± 0.9	11.3 ± 0.9	6.1 ± 0.8	8.4 ± 0.5	8.1 ± 0.6	5.5 ± 0.5	8.3 ± 1.2	9.8 ± 0.6
	PD	15.7 ± 1.0	17.8 ± 1.1	19.8 ± 1.1	15.6 ± 2.8	17.3 ± 1.8	17.9 ± 1.5	14.5 ± 1.0	15.7 ± 1.4	16.5 ± 1.6
	mDP	9.8 ± 0.3	9.8 ± 0.5	8.8 ± 0.4	10.1 ± 0.4	9.8 ± 0.3	9.9 ± 0.4	8.8 ± 0.3	7.5 ± 0.3	9 ± 0.4
	**FL**	10.0 ± 0.5	7.9 ± 0.4	6.6 ± 0.3	8.5 ± 1.0	8.4 ± 0.3	8.6 ± 0.4	8.5 ± 0.4	7.4 ± 0.4	6.4 ± 0.3
	KA	4.1 ± 0.5	3.2 ± 0.4	2.7 ± 0.3	3.6 ± 0.5	3.5 ± 0.2	4.0 ± 0.3	3.9 ± 0.2	3.5 ± 0.3	2.7 ± 0.2
	QU	5.4 ± 0.1	4.3 ± 0.1	3.6 ± 0.1	4.5 ± 0.5	4.5 ± 0.1	4.3 ± 0.1	4.1 ± 0.2	3.5 ± 0.2	3.5 ± 0.1
	MY	0.4 ± 0.1	0.3 ± 0.1	0.3 ± 0.1	0.4 ± 0.1	0.4 ± 0.1	0.4 ± 0.1	0.5 ± 0.1	0.4 ± 0.1	0.3 ± 0.1
	**QA**	7.3 ± 0.9	5.0 ± 0.4	3.7 ± 0.4	6.5 ± 0.7	5.1 ± 0.4	4.8 ± 0.4	6.5 ± 0.5	4.6 ± 0.3	2.7 ± 0.3
Activities	**OX**	18.0 ± 0.9	16.8 ± 1.4	17.5 ± 1.4	19.8 ± 1.1	19.1 ± 0.9	20.3 ± 1.1	18.3 ± 1.0	17.5 ± 0.7	15.9 ± 1.0
	**PPC**	16.9 ± 1.7	12.7 ± 0.9	11.3 ± 1.1	13.1 ± 0.6	11.3 ± 1.1	7.4 ± 1.1	8.6 ± 0.9	10.6 ± 1.1	8.7 ± 1.2

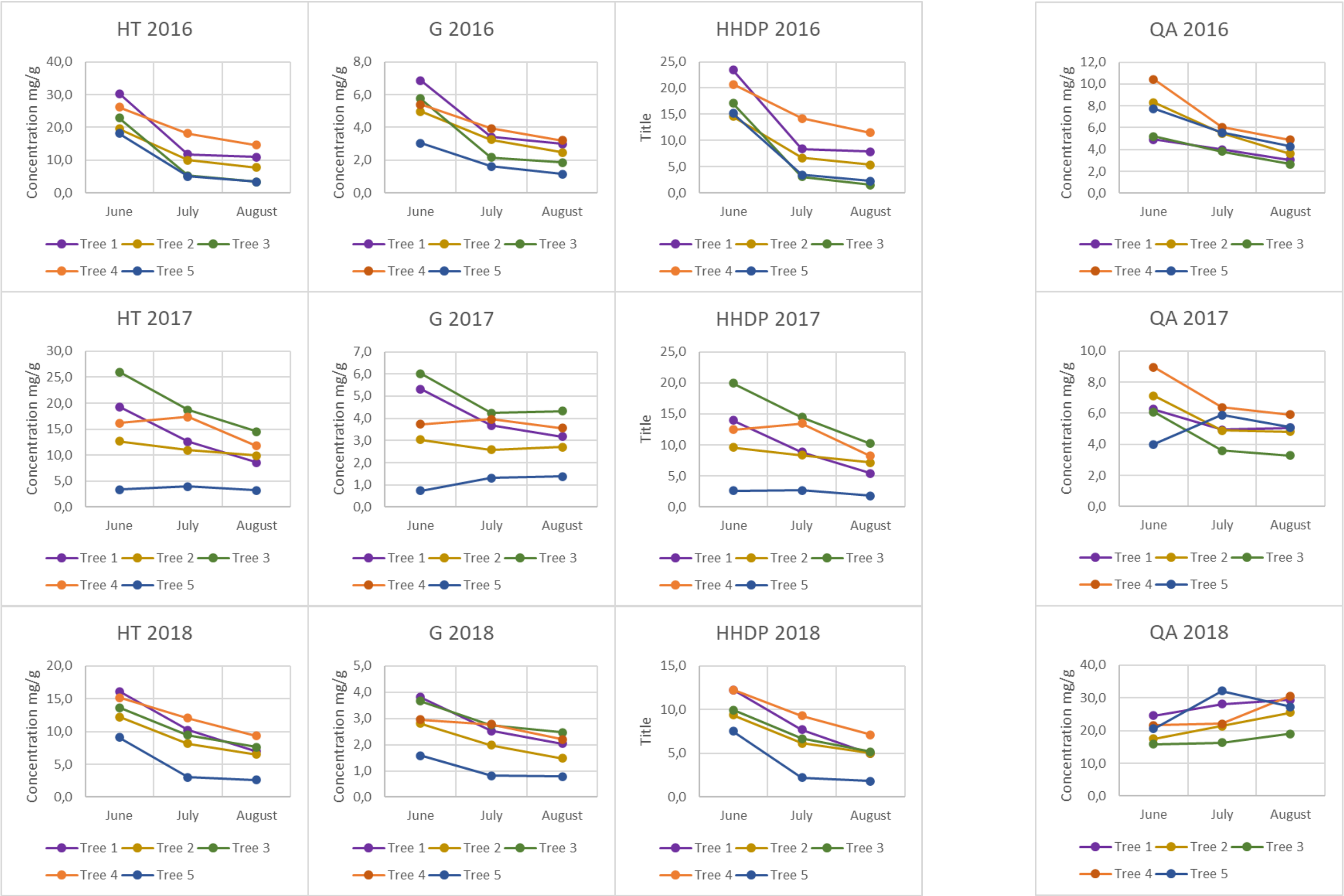

Figure S11A. The seasonal and yearly changes of hydrolysable tannins (HT), galloyl and hexahydroxydiphenoyl derivatives (G and HHDP) and quinic acid derivatives (QA) of the five *Betula pubescens* trees.

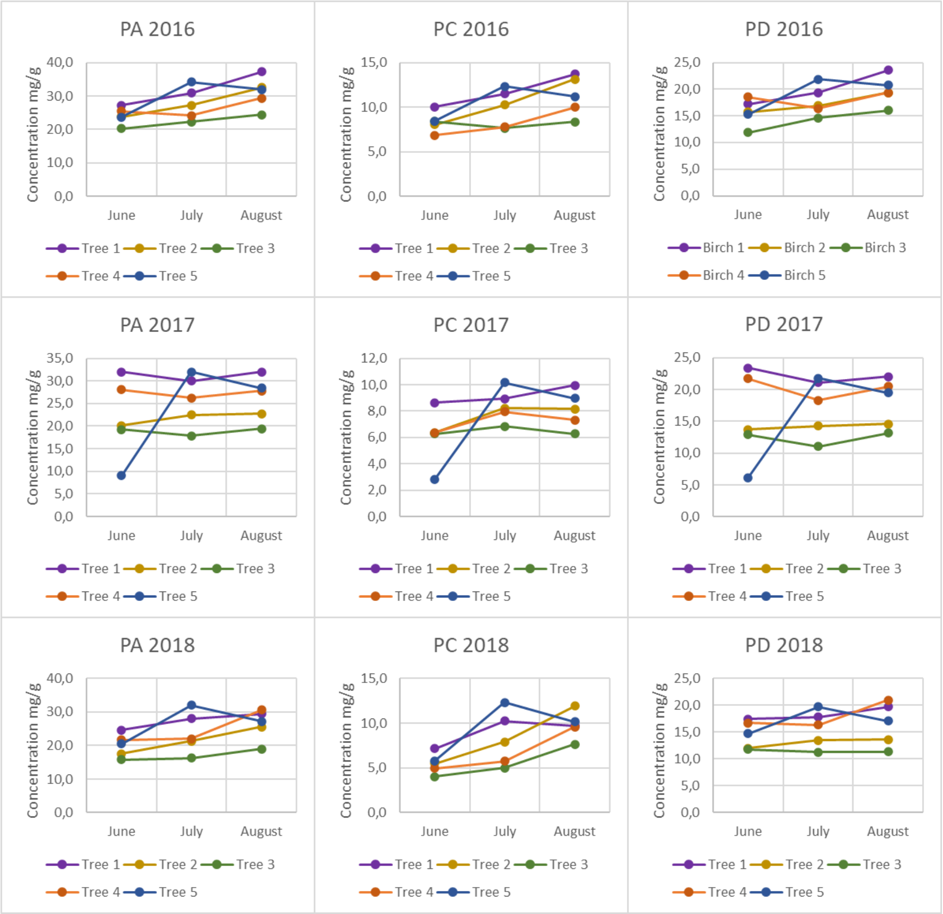

Figure S11B. The seasonal and yearly changes of proanthocyanidins (PA), procyanidins (PC) and prodelphinidins (PD) of the five *Betula pubescens* trees.

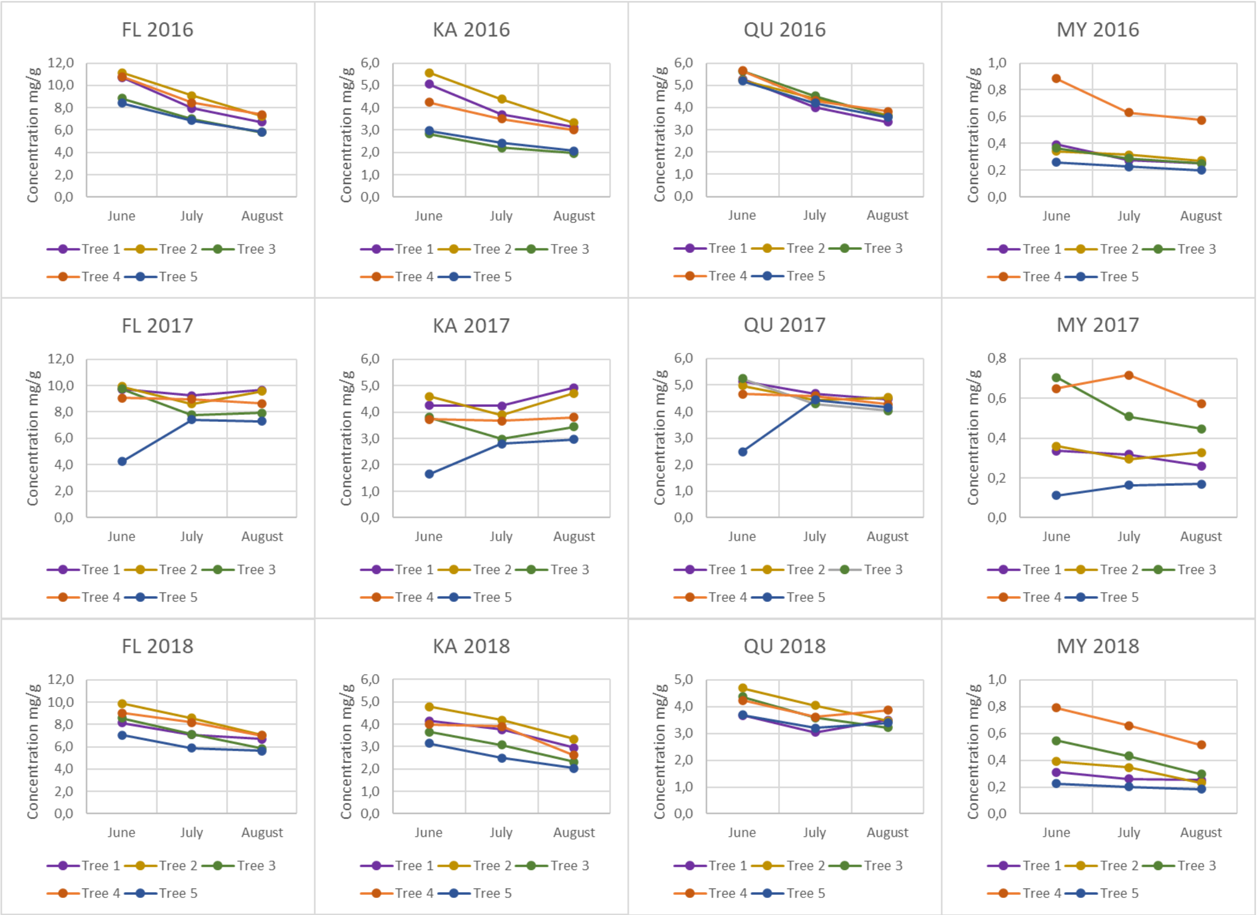

Figure S11C. The seasonal and yearly changes of flavonol glycosides (FL), kaempferol glycosides (KA), quercetin glycosides (QU) and myricetin glycosides (MY) of the five *Betula pubescens* trees.

Table S2. Initial concentrations of the *Quercus robur* population. Values are averages of the five individual trees ± standard error of the mean.

Quercus robur		2016			2017			2018		
		June	July	August	June	July	August	June	July	August
Polyphenols	**TP**	86.5 ± 3.7	91.3 ± 4.4	87.8 ± 5.3	109.7 ± 5.4	110.2 ± 4.0	111.1 ± 4.7	93.6 ± 6.5	97.3 ± 6.1	105.7 ± 19.0
	**HT**	35.4 ± 1.7	21.3 ± 1.3	20.7 ± 1.5	43.7 ± 2.9	27.5 ± 1.7	23.4 ± 1.5	26.9 ± 1.4	22.0 ± 1.5	20.1 ± 1.3
	G	4.8 ± 0.4	2.4 ± 0.2	2.2 ± 0.1	6.1 ± 0.7	3.2 ± 0.2	2.8 ± 0.2	3.7 ± 0.4	2.8 ± 0.3	2.4 ± 0.3
	HHDP	30.6 ± 1.3	19.0 ± 1.2	18.4 ± 1.4	37.6 ± 2.3	24.3 ± 1.7	20.5 ± 1.4	23.3 ± 1.3	19.2 ± 1.5	17.7 ± 1.3
	**PA**	1.7 ± 0.8	32 ± 4.1	37.5 ± 6.3	3.1 ± 1.0	38.8 ± 5.5	39.0 ± 6.3	19.2 ± 6.1	32.5 ± 7.3	30.1 ± 8.7
	PC	1.7 ± 0.8	23.8 ± 3.8	27.0 ± 4.4	2.9 ± 0.9	27.3 ± 4.3	28.3 ± 5	15.6 ± 5.1	24.8 ± 6.6	23.1 ± 6.7
	PD	NS	8.2 ± 1.1	10.5 ± 2.5	0.2 ± 0.2	11.5 ± 2.1	10.7 ± 2.1	3.7 ± 1.4	7.7 ± 1.0	7.0 ± 2.4
	mDP	2.7 ± 0.6	5.8 ± 0.3	5.4 ± 0.3	3.1 ± 0.5	5.7 ± 0.4	5.5 ± 0.3	5.3 ± 0.4	5.5 ± 0.3	5.7 ± 0.3
	**FL**	12.3 ± 0.5	5.2 ± 0.6	4.9 ± 0.9	11.3 ± 0.7	5.5 ± 0.9	5.9 ± 0.8	7.5 ± 0.7	5.4 ± 0.7	3.8 ± 0.8
	KA	8.5 ± 0.6	2.5 ± 0.2	2.0 ± 0.3	7.1 ± 0.5	2.0 ± 0.3	2.1 ± 0.4	5.0 ± 0.4	2.8 ± 0.3	1.6 ± 0.3
	QU	3.7 ± 0.6	2.5 ± 0.3	2.6 ± 0.5	4.0 ± 0.6	3.2 ± 0.6	3.5 ± 0.5	2.4 ± 0.5	2.3 ± 0.4	2.0 ± 0.4
	MY	0.2 ± 0.1	0.2 ± 0.0	0.3 ± 0.1	0.2 ± 0.1	0.3 ± 0.1	0.3 ± 0.1	0.1 ± 0.1	0.3 ± 0.1	0.2 ± 0.1
	**QA**	0.6 ± 0.0	0.7 ± 0.0	0.6 ± 0.1	0.6 ± 0.1	0.8 ± 0.1	0.6 ± 0.0	1.0 ± 0.1	0.9 ± 0.1	0.7 ± 0.0
Activities	**OX**	36.7 ± 1.8	30.4 ± 0.8	28.4 ± 1.6	44.0 ± 2.3	33.5 ± 2.6	33.0 ± 2.8	38.0 ± 3	37.3 ± 1.7	46.8 ± 17.1
	**PPC**	34.5 ± 3.8	37.5 ± 3	34.8 ± 3.4	45.1 ± 3	44.8 ± 2.0	34.9 ± 2.2	34.0 ± 2.5	35.1 ± 2.4	33.1 ± 3.6

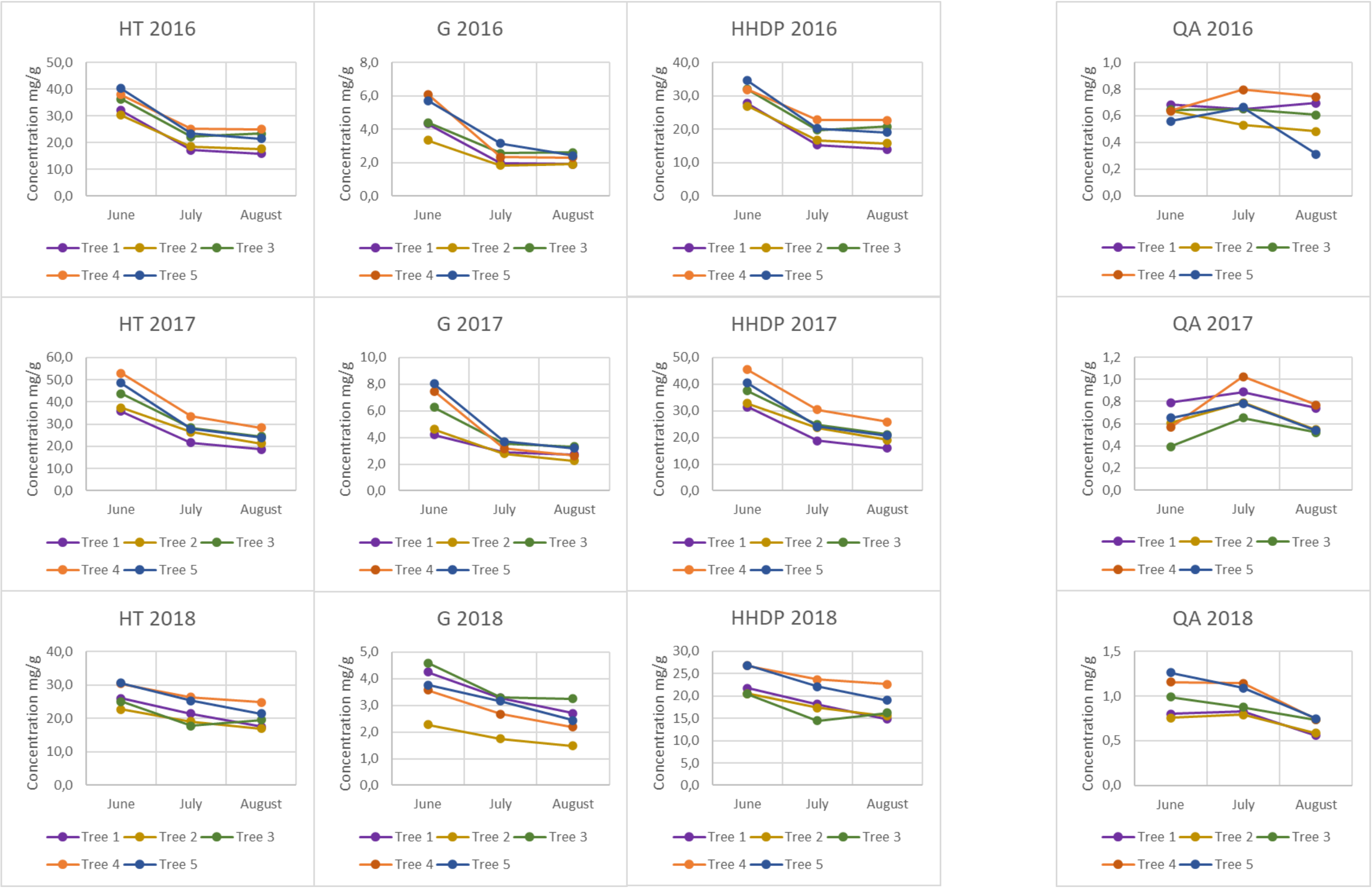

Figure S12A. The seasonal and yearly changes of hydrolysable tannins (HT), galloyl and hexahydroxydiphenoyl derivatives (G and HHDP) and quinic acid derivatives (QA) of the five *Quercus robur* trees.

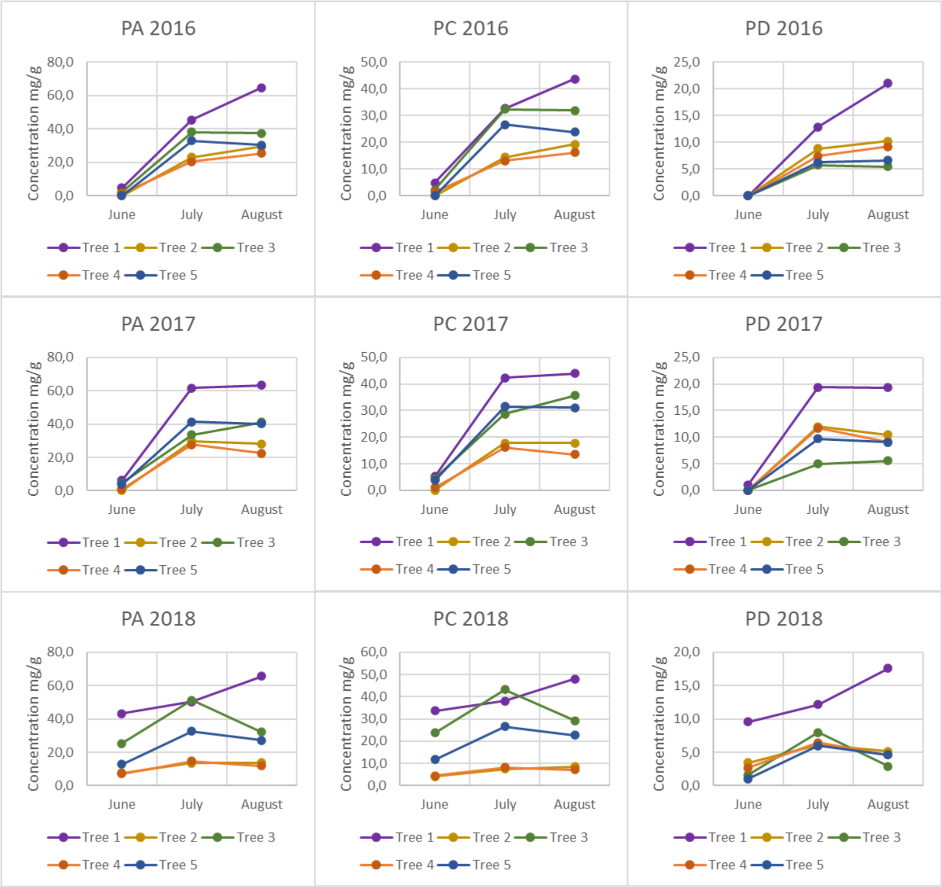

Figure S12B. The seasonal and yearly changes of proanthocyanidins (PA), procyanidins (PC) and prodelphinidins (PD) of the five *Quercus robur* trees.

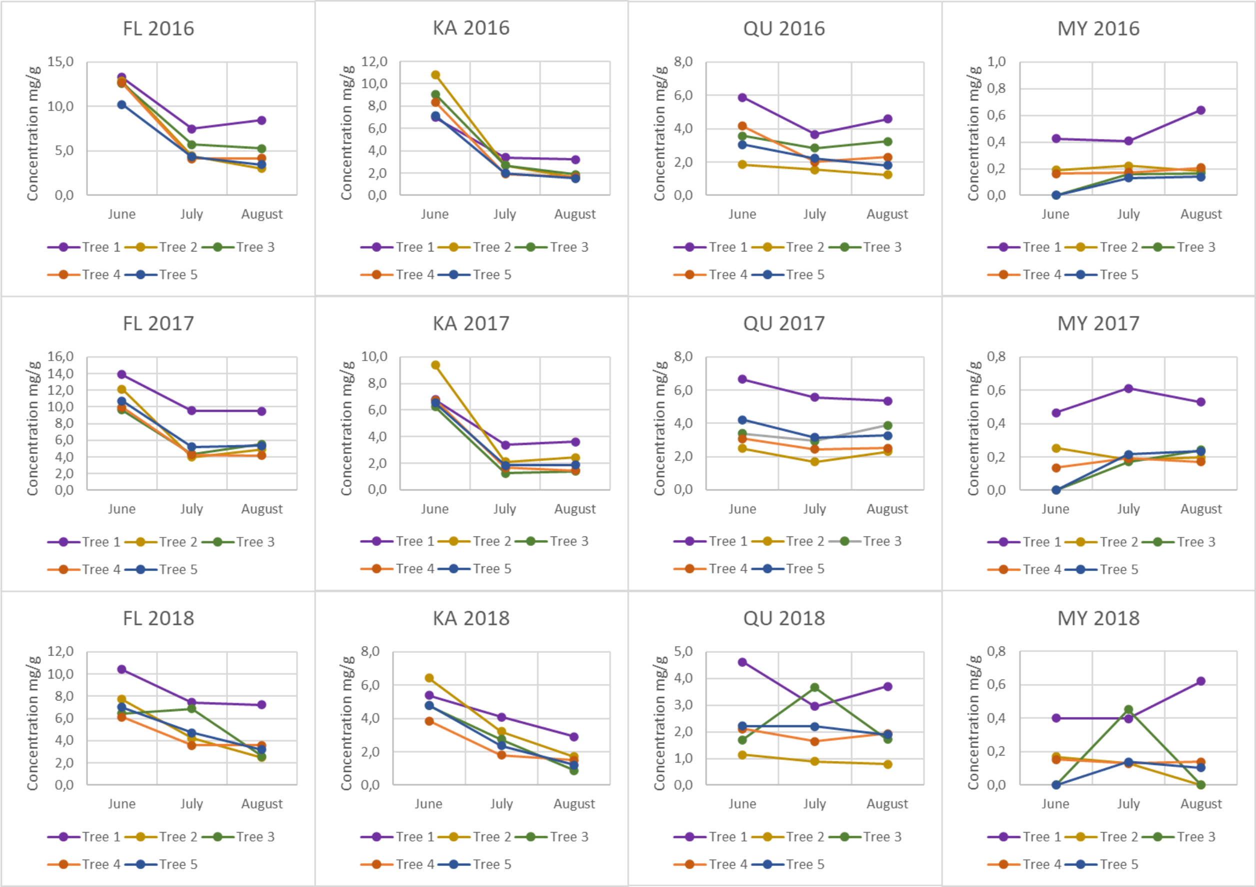

Figure S12C. The seasonal and yearly changes of flavonol glycosides (FL), kaempferol glycosides (KA), quercetin glycosides (QU) and myricetin glycosides (MY) of the five *Quercus robur* trees.

Table S3. Initial concentrations of the *Acer platanoides* population. Values are averages of the five individual trees ± standard error of the mean.

Acer platanoides		2016			2017			2018		
		June	July	August	June	July	August	June	July	August
Polyphenols	**TP**	91.0 ± 9.4	82.9 ± 7.2	91.1 ± 7.8	120.1 ± 10.9	145.9 ± 15.8	109.5 ± 9.0	83.2 ± 13.2	92.8 ± 10.7	121.9 ± 18.8
	**HT**	19.3 ± 1.9	15.6 ± 1.1	14.6 ± 1.3	21.3 ± 1	22.4 ± 1.2	17.8 ± 1	15 ± 1.8	15.1 ± 2	17.2 ± 1.5
	G	19.3 ± 1.9	15.6 ± 1.1	14.6 ± 1.3	21.3 ± 1	22.4 ± 1.2	17.8 ± 1	15 ± 1.8	15.1 ± 2	17.2 ± 1.5
	HHDP	ND	ND	ND	ND	ND	ND	ND	ND	ND
	**PA**	10.9 ± 2	27 ± 4.3	31.4 ± 3.8	15.5 ± 2.2	37.8 ± 5.8	25.4 ± 2.6	10.4 ± 4	23.5 ± 3.3	41 ± 10.4
	PC	10.9 ± 2	27 ± 4.3	31.1 ± 3.6	15.5 ± 2.2	37.3 ± 5.6	25.4 ± 2.6	10.4 ± 4	23.5 ± 3.3	40.4 ± 9.9
	PD	NS	NS	0.3 ± 0.2	NS	0.5 ± 0.3	NS	NS	NS	0.6 ± 0.5
	mDP	3.4 ± 0.1	3.4 ± 0.1	3.2 ± 0.1	3.2 ± 0.1	3.2 ± 0.1	3.0 ± 0.0	3.1 ± 0.1	3.1 ± 0.1	3.8 ± 0.2
	**FL**	2.3 ± 0.2	2.0 ± 0.3	1.9 ± 0.1	2.3 ± 0.3	2.8 ± 0.4	2.3 ± 0.2	1.7 ± 0.3	1.9 ± 0.3	4.3 ± 0.5
	KA	1.6 ± 0.1	1.2 ± 0.1	1.2 ± 0.0	1.2 ± 0.2	1.1 ± 0.1	1.2 ± 0.1	1.1 ± 0.2	1.0 ± 0.1	1.4 ± 0.1
	QU	0.7 ± 0.1	0.8 ± 0.2	0.7 ± 0.1	1.0 ± 0.2	1.8 ± 0.3	1.0 ± 0.2	0.5 ± 0.2	1.0 ± 0.3	2.9 ± 0.4
	MY	ND	ND	ND	ND	ND	ND	ND	ND	ND
	**QA**	1.6 ± 0.4	0.9 ± 0.3	0.6 ± 0.2	1.3 ± 0.4	1.0 ± 0.3	0.7 ± 0.2	1.1 ± 0.4	0.9 ± 0.3	0.6 ± 0.2
Activities	**OX**	2.3 ± 1.3	3.0 ± 0.7	6.3 ± 2.4	1.2 ± 0.7	4.1 ± 1.4	4.1 ± 1.3	2.7 ± 0.8	2.8 ± 0.8	8.2 ± 2.1
	**PPC**	54 ± 7.6	52.5 ± 5.7	55.2 ± 6.0	79.7 ± 7.6	83.4 ± 6.3	63.5 ± 7.4	49.1 ± 10.4	51.2 ± 7.5	69.7 ± 9.6

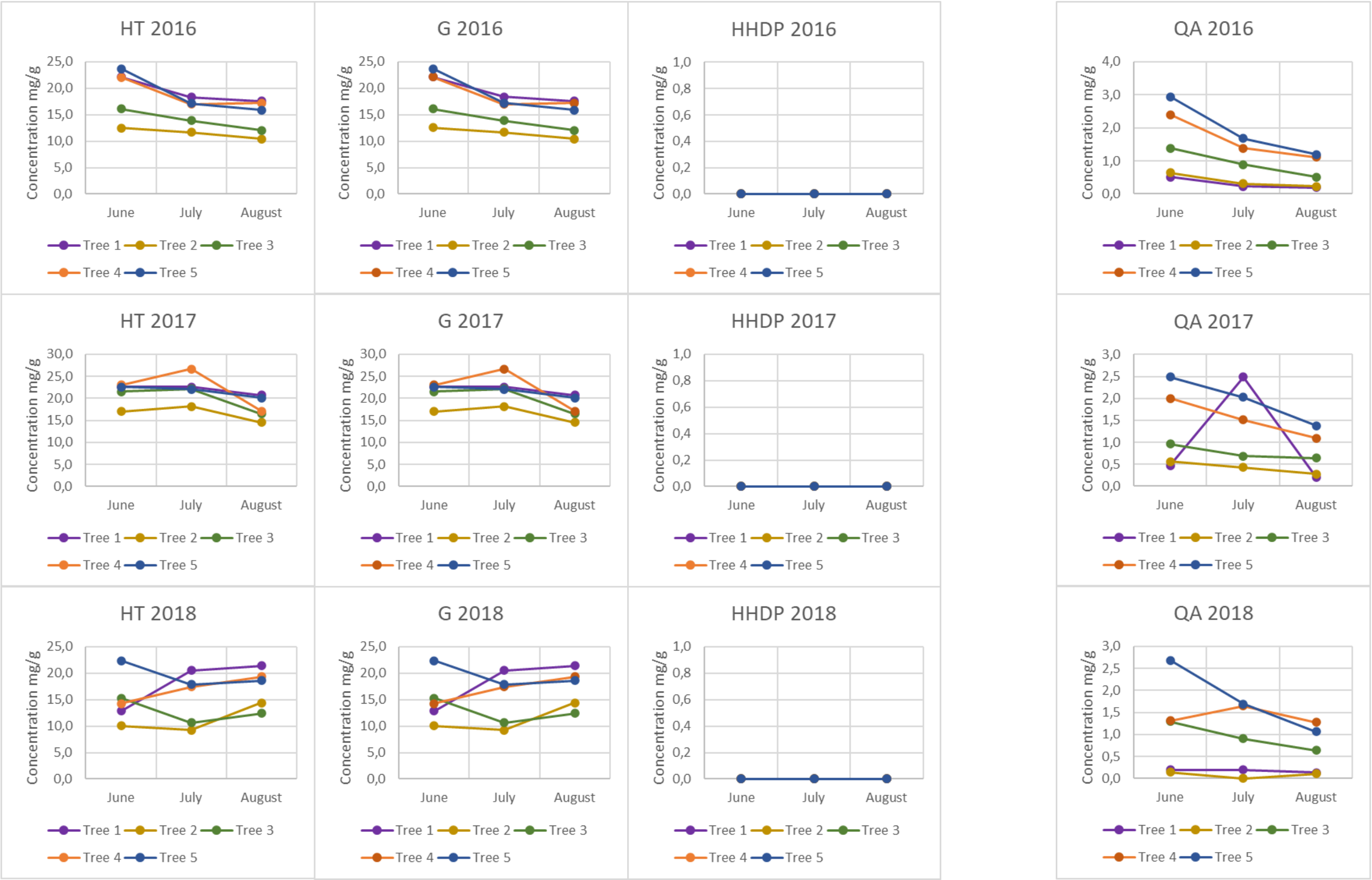

Figure S13A. The seasonal and yearly changes of hydrolysable tannins (HT), galloyl and hexahydroxydiphenoyl derivatives (G and HHDP) and quinic acid derivatives (QA) of the five *Acer platanoides* trees.

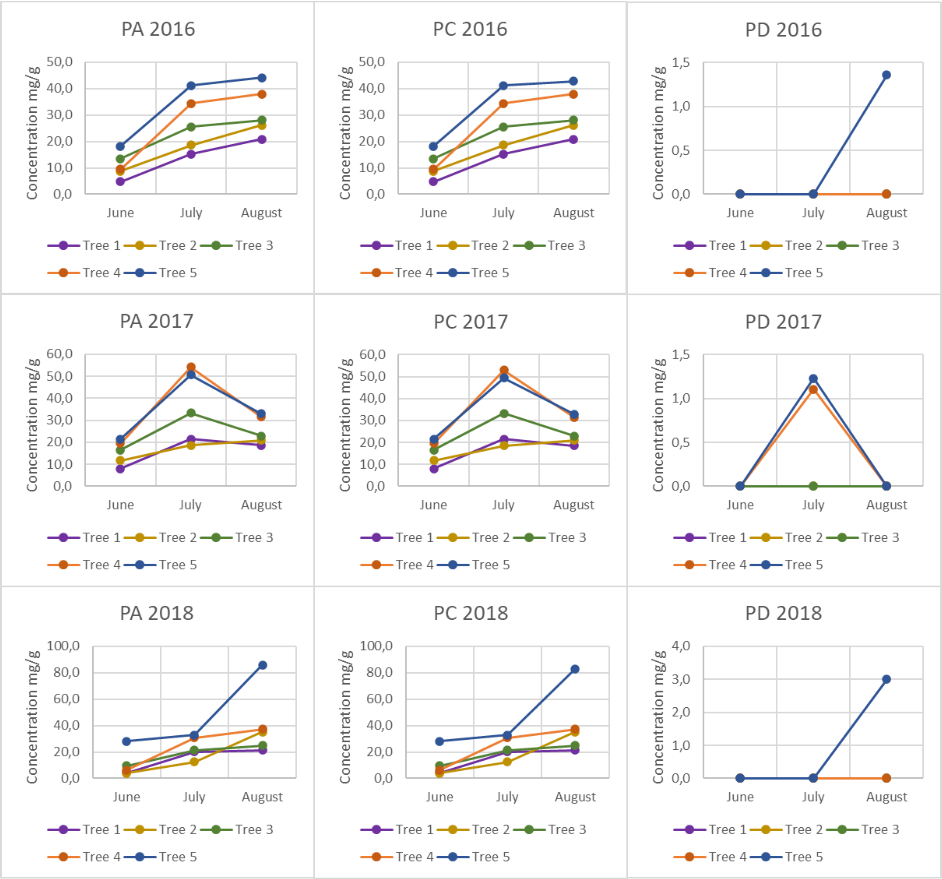

Figure S13B. The seasonal and yearly changes of proanthocyanidins (PA), procyanidins (PC) and prodelphinidins (PD) of the five *Acer platanoides* trees.

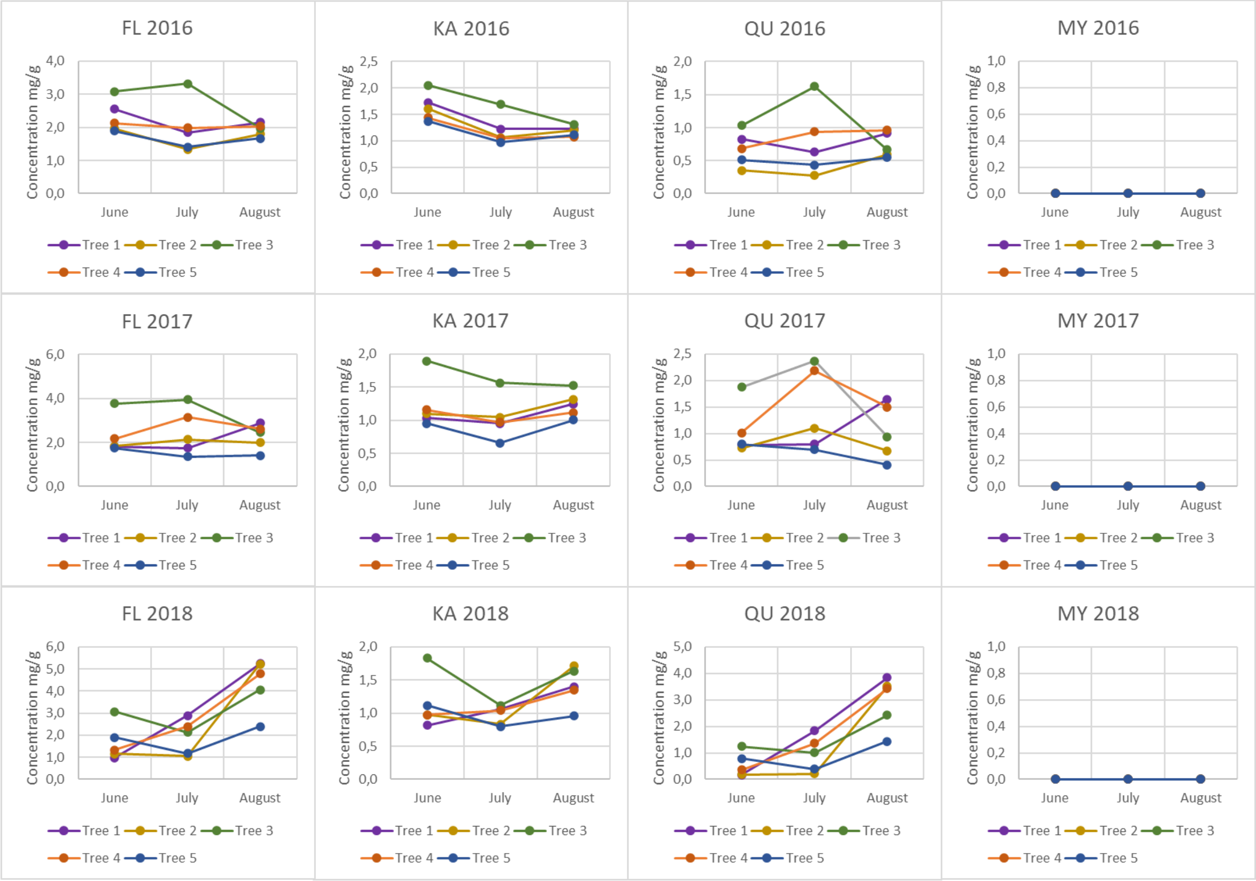

Figure S13C. The seasonal and yearly changes of flavonol glycosides (FL), kaempferol glycosides (KA), quercetin glycosides (QU) and myricetin glycosides (MY) of the five *Acer platanoides* trees.

Table S4. Initial concentrations of the *Alnus glutinosa* population. Values are averages of the five individual trees ± standard error of the mean.

	Alnus glutinosa	2016			2017			2018		
		June	July	August	June	July	August	June	July	August
Polyphenols	**TP**	55.9 ± 6.1	46.2 ± 4.8	42.4 ± 5.6	68.5 ± 13.6	64.1 ± 9.7	71.3 ± 8.7	74.6 ± 8.2	65.4 ± 6.5	70.2 ± 7.3
	**HT**	8.5 ± 5.4	7.1 ± 4.9	5.3 ± 3.2	26.1 ± 13.5	29.4 ± 14	23.3 ± 10.4	18.3 ± 11.9	14.6 ± 9.6	20.9 ± 9.7
	G	1.9 ± 0.9	1.2 ± 0.6	1.1 ± 0.4	3.0 ± 1.4	3.1 ± 1.3	2.8 ± 1.0	2.3 ± 1.1	1.8 ± 0.8	2.1 ± 0.8
	HHDP	6.5 ± 4.5	5.9 ± 4.4	4.2 ± 2.8	23.1 ± 12.1	26.4 ± 12.8	20.5 ± 9.3	16.0 ± 10.8	12.9 ± 8.9	18.8 ± 9.0
	**PA**	1.2 ± 0.4	1.0 ± 0.4	1.4 ± 0.4	1.4 ± 0.5	2.1 ± 0.5	2.0 ± 0.5	1.7 ± 0.6	2.4 ± 0.6	2.4 ± 0.4
	PC	1.2 ± 0.4	1.0 ± 0.4	1.4 ± 0.4	1.4 ± 0.5	2.1 ± 0.5	2.0 ± 0.5	1.7 ± 0.6	2.4 ± 0.6	2.4 ± 0.4
	PD	ND	ND	ND	ND	ND	ND	ND	ND	ND
	mDP	2.1 ± 0.2	2.1 ± 0.2	2.5 ± 0.1	1.6 ± 0.2	2.3 ± 0.1	2.6 ± 0.1	2.1 ± 0.2	2.3 ± 0.2	2.9 ± 0.2
	**FL**	2.6 ± 0.2	2.1 ± 0.1	1.9 ± 0.2	2.1 ± 0.1	2.4 ± 0.2	2.8 ± 0.3	2.2 ± 0.2	2.1 ± 0.1	2.1 ± 0.1
	KA	0.3 ± 0.0	0.3 ± 0.0	0.2 ± 0.0	0.3 ± 0.0	0.3 ± 0.0	0.4 ± 0.1	0.4 ± 0.0	0.4 ± 0.0	0.3 ± 0.0
	QU	2.3 ± 0.1	1.8 ± 0.1	1.6 ± 0.1	1.8 ± 0.1	2.1 ± 0.2	2.4 ± 0.2	1.9 ± 0.1	1.8 ± 0.1	1.8 ± 0.1
	MY	ND	ND	ND	ND	ND	ND	ND	ND	ND
	**QA**	3.8 ± 0.7	2.9 ± 0.5	3.1 ± 0.6	3.1 ± 0.5	3.6 ± 0.7	3.6 ± 0.8	3.2 ± 0.4	3.2 ± 0.5	2.6 ± 0.4
Activities	**OX**	25.9 ± 2.4	20.6 ± 2.0	19.3 ± 2.8	26.9 ± 3.6	25.2 ± 2.9	29.6 ± 1.8	34.6 ± 1.9	29.8 ± 1.7	30.3 ± 3.2
	**PPC**	5.0 ± 2.8	2.7 ± 2.1	1.1 ± 1.0	14.3 ± 7.0	12.8 ± 5.1	8.2 ± 4.2	9.7 ± 6.2	7.8 ± 4.7	9.2 ± 4.1

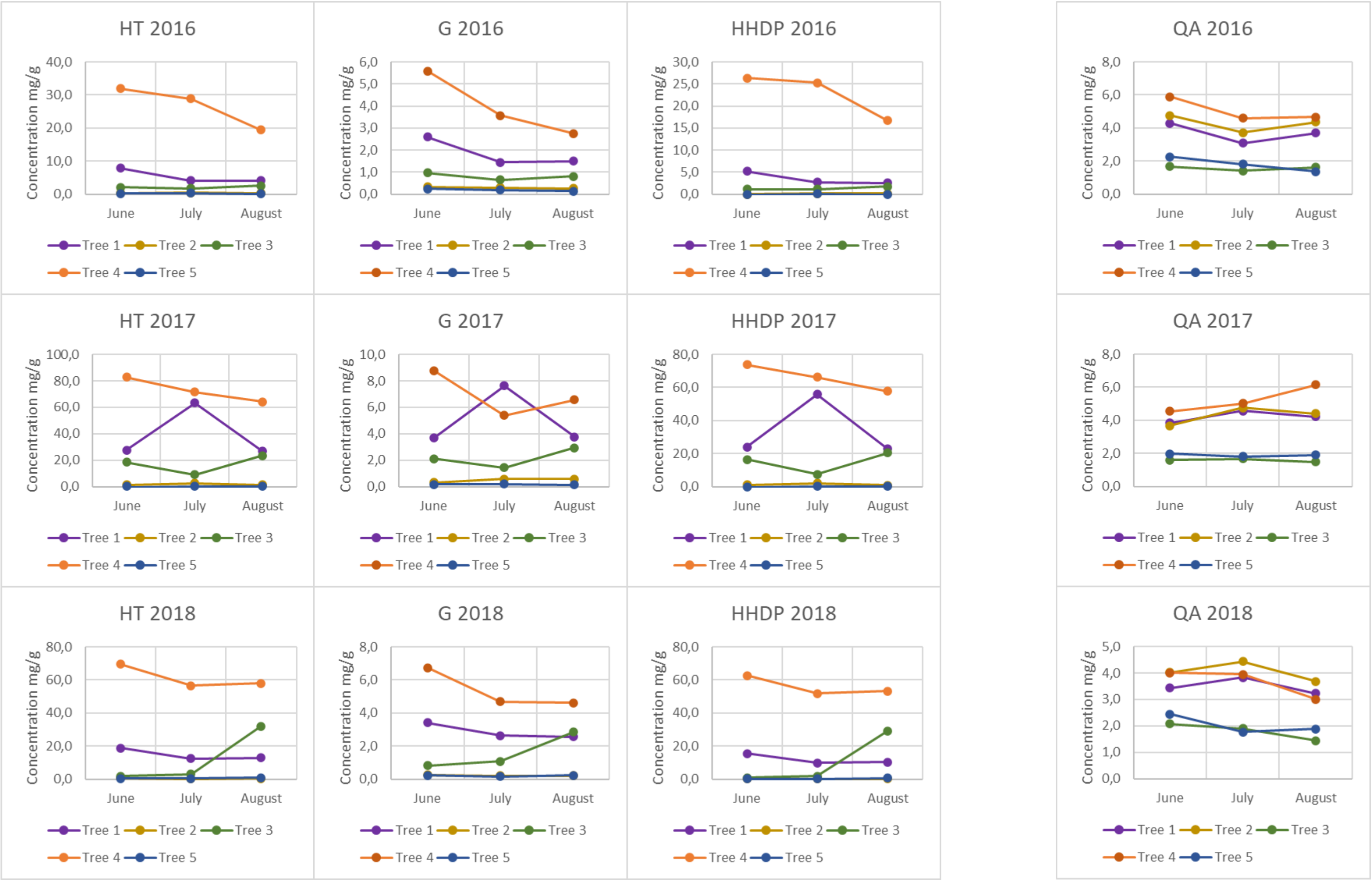

Figure S14A. The seasonal and yearly changes of hydrolysable tannins (HT), galloyl and hexahydroxydiphenoyl derivatives (G and HHDP) and quinic acid derivatives (QA) of the five *Alnus glutinosa* trees.

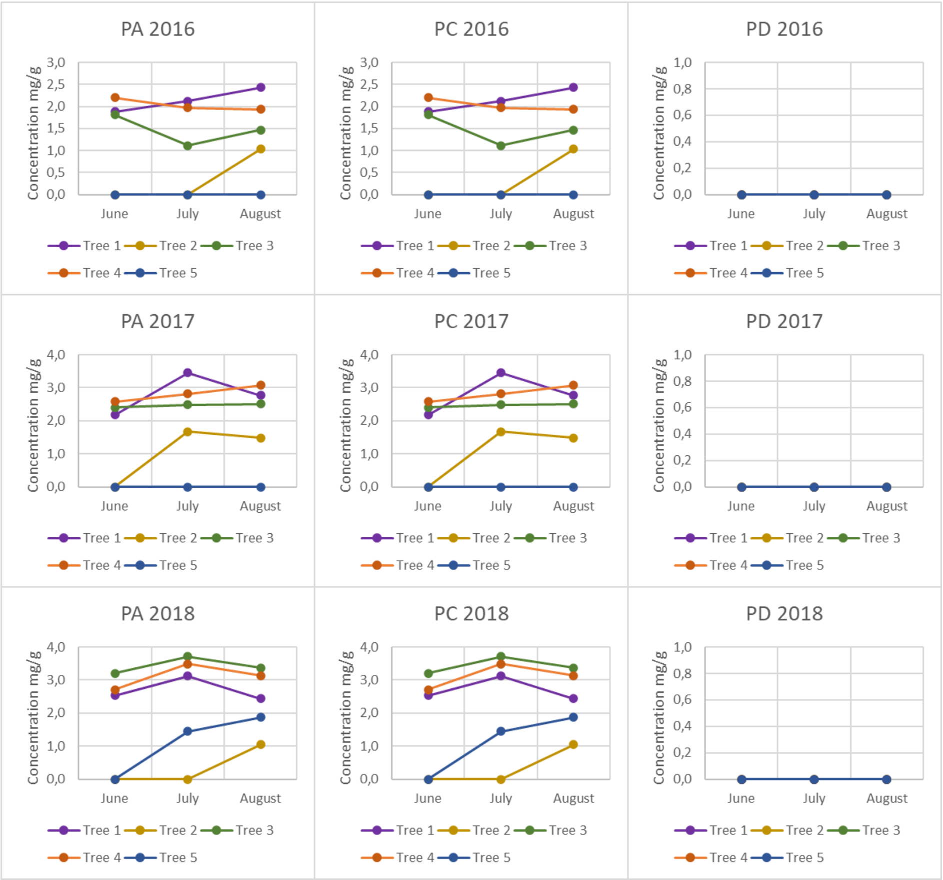

Figure S14B. The seasonal and yearly changes of proanthocyanidins (PA), procyanidins (PC) and prodelphinidins (PD) of the five *Alnus glutinosa* trees.

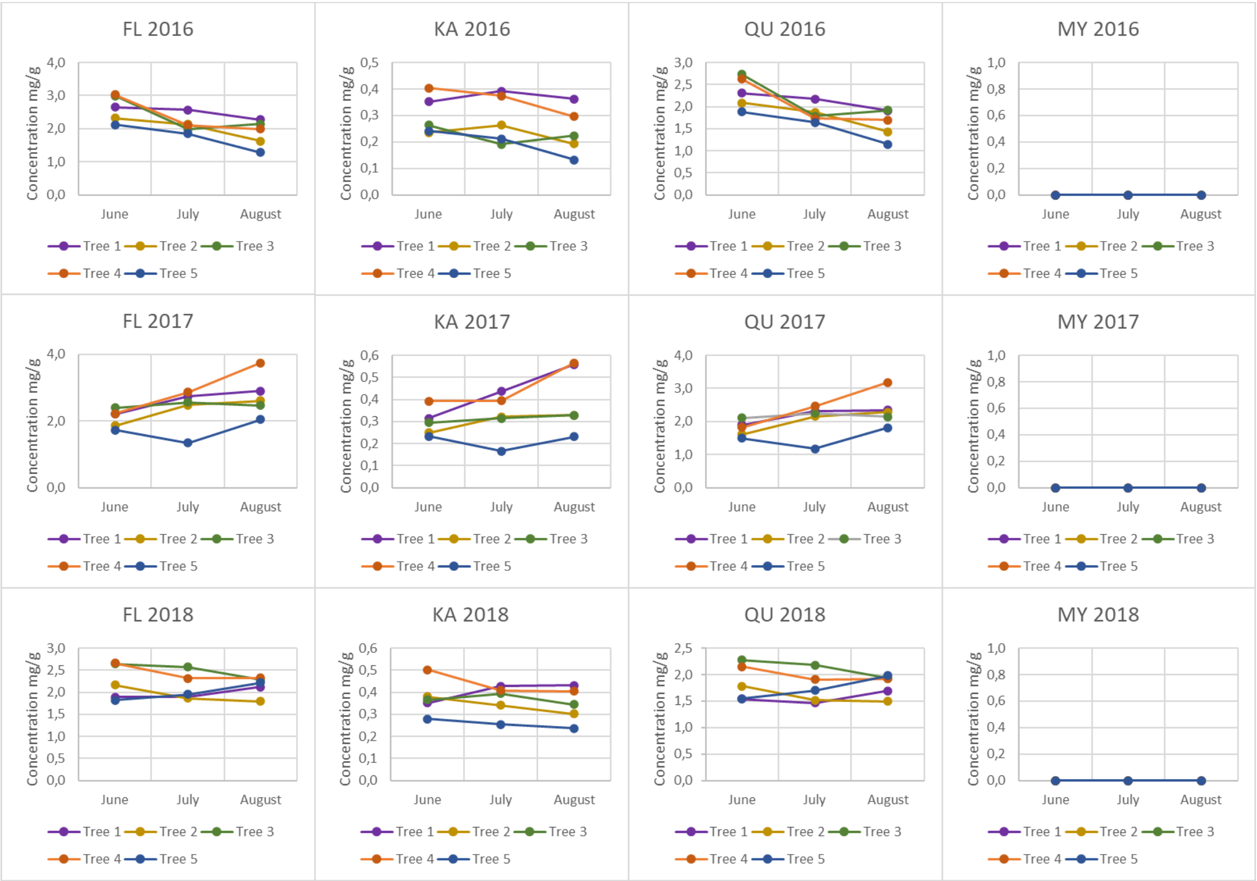

Figure S14C. The seasonal and yearly changes of flavonol glycosides (FL), kaempferol glycosides (KA), quercetin glycosides (QU) and myricetin glycosides (MY) of the five *Alnus glutinosa* trees.

Table S5. Initial concentrations of the *Alnus incana* population. Values are averages of the five individual trees ± standard error of the mean. Only three trees were available in 2018.

Alnus incana		2016			2017			2018		
		June	July	August	June	July	August	June	July	August
Polyphenols	**TP**	41 ± 4.5	43.5 ± 4.4	49.8 ± 3.2	57.7 ± 1.9	58.3 ± 2.5	60.8 ± 2.9	50.4 ± 4.2	49.5 ± 2.1	49.2 ± 0.3
	**HT**	ND	ND	ND	ND	ND	ND	ND	ND	ND
	G	ND	ND	ND	ND	ND	ND	ND	ND	ND
	HHDP	ND	ND	ND	ND	ND	ND	ND	ND	ND
	**PA**	NS	0.3 ± 0.3	2.1 ± 0.6	0.2 ± 0.2	1.0 ± 0.3	1.5 ± 0.5	0.9 ± 0.4	0.4 ± 0.3	0.6 ± 0.5
	PC	NS	0.3 ± 0.3	2.1 ± 0.6	0.2 ± 0.2	1.0 ± 0.3	1.5 ± 0.5	0.9 ± 0.4	0.4 ± 0.3	0.6 ± 0.5
	PD	ND	ND	ND	ND	ND	ND	ND	ND	ND
	mdp	2.0 ± 0.1	1.9 ± 0.1	2.7 ± 0.3	2.0 ± 0.1	2.0 ± 0.1	2.5 ± 0.1	1.9 ± 0	1.6 ± 0.1	2.1 ± 0.1
	**FL**	5.6 ± 0.2	5.5 ± 0.3	5.7 ± 0.2	5.5 ± 0.2	6 ± 0.3	6.2 ± 0.4	5.4 ± 0.3	5.8 ± 0.3	5.7 ± 0.2
	KA	0.4 ± 0.0	0.3 ± 0.0	0.3 ± 0.0	0.3 ± 0.0	0.3 ± 0	0.3 ± 0.0	0.4 ± 0.0	0.4 ± 0.0	0.4 ± 0.0
	QU	5.2 ± 0.2	5.2 ± 0.3	5.4 ± 0.2	5.2 ± 0.2	5.7 ± 0.3	5.9 ± 0.4	5.0 ± 0.3	5.4 ± 0.3	5.3 ± 0.2
	MY	ND	ND	ND	ND	ND	ND	ND	ND	ND
	**QA**	4.8 ± 0.4	4.3 ± 0.5	3.9 ± 0.4	4.8 ± 0.3	5.2 ± 0.4	5.3 ± 0.4	5.0 ± 0.5	5.0 ± 0.4	3.8 ± 0.5
Activities	**OX**	15.4 ± 2.2	17.8 ± 2.6	20.6 ± 1.2	24.3 ± 0.7	24.8 ± 1.1	25.6 ± 1.1	21.1 ± 2.1	20.4 ± 0.8	20.8 ± 0.9
	**PPC**	ND	ND	ND	ND	ND	ND	ND	ND	ND

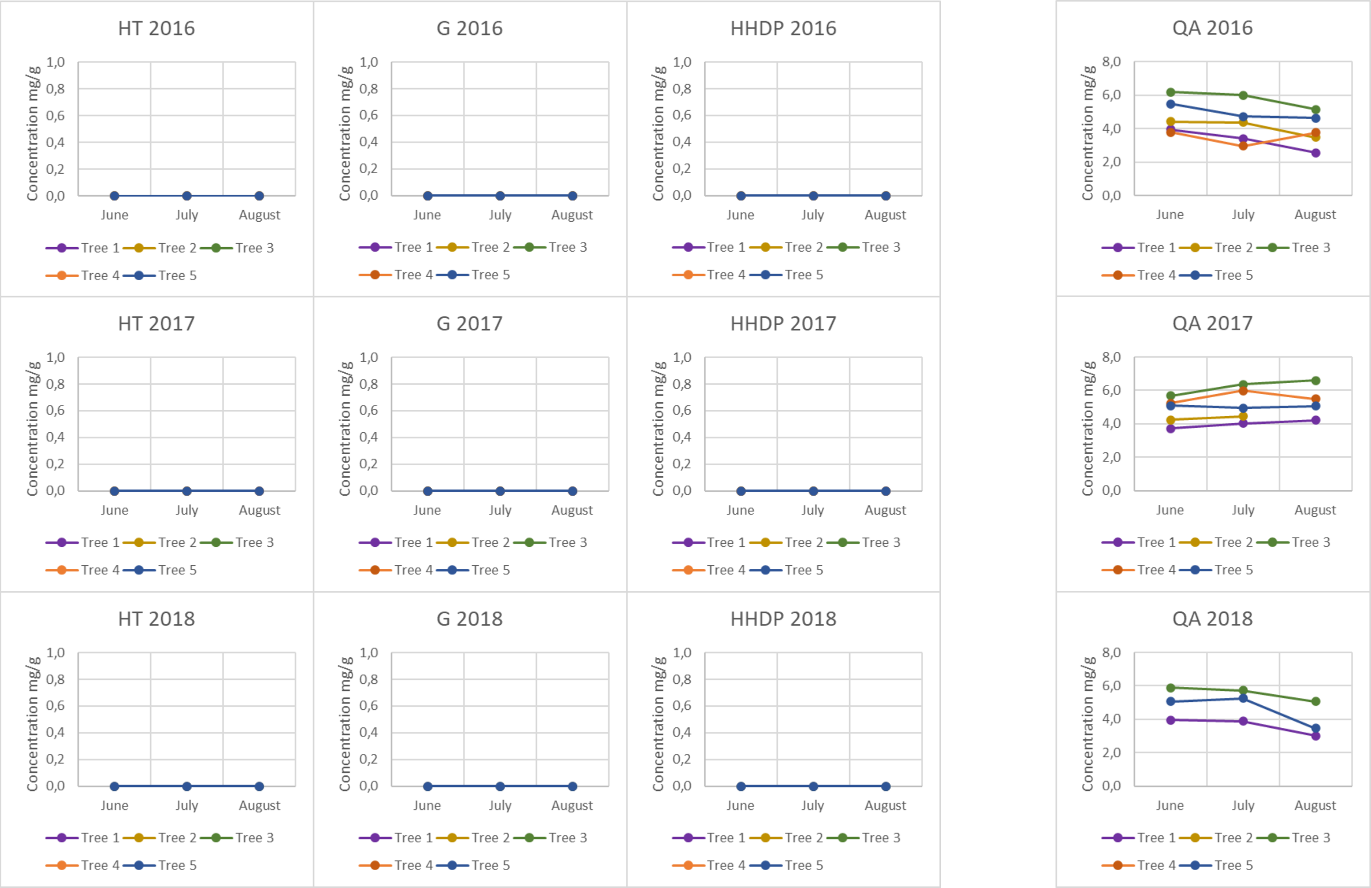

Figure S15A. The seasonal and yearly changes of hydrolysable tannins (HT), galloyl and hexahydroxydiphenoyl derivatives (G and HHDP) and quinic acid derivatives (QA) of the three to five *Alnus incana* trees. Only three trees were available in 2018.

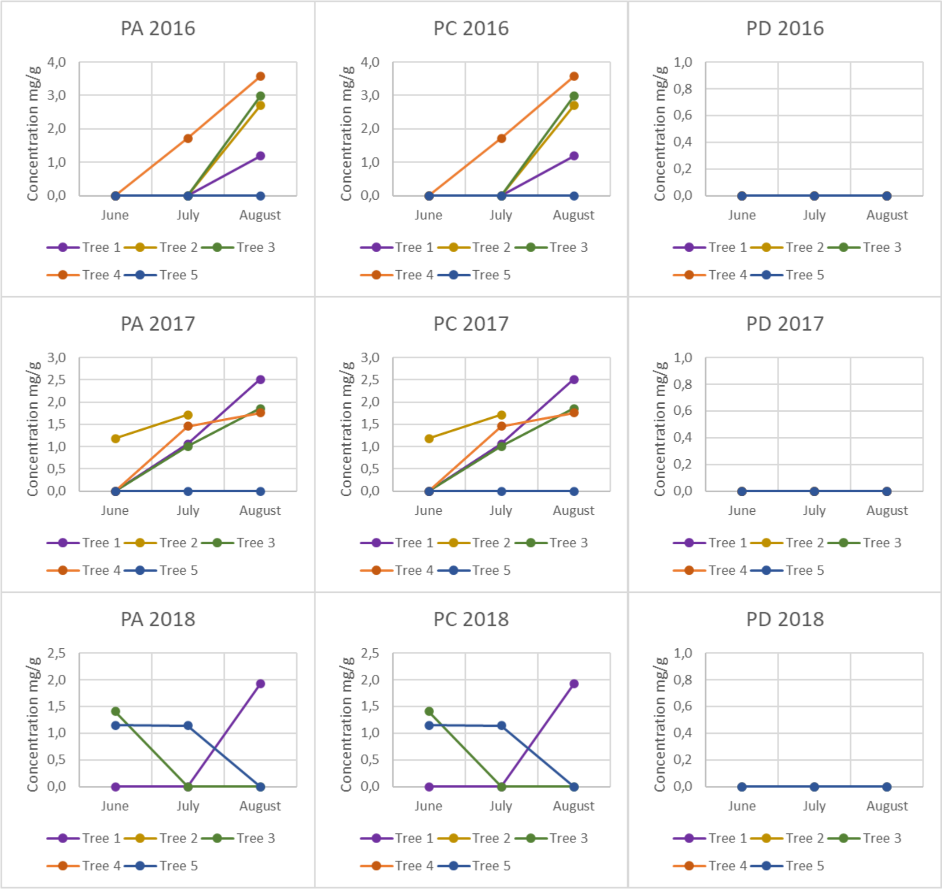

Figure S15B. The seasonal and yearly changes of proanthocyanidins (PA), procyanidins (PC) and prodelphinidins (PD) of the three to five *Alnus incana* trees. Only three trees were available in 2018.

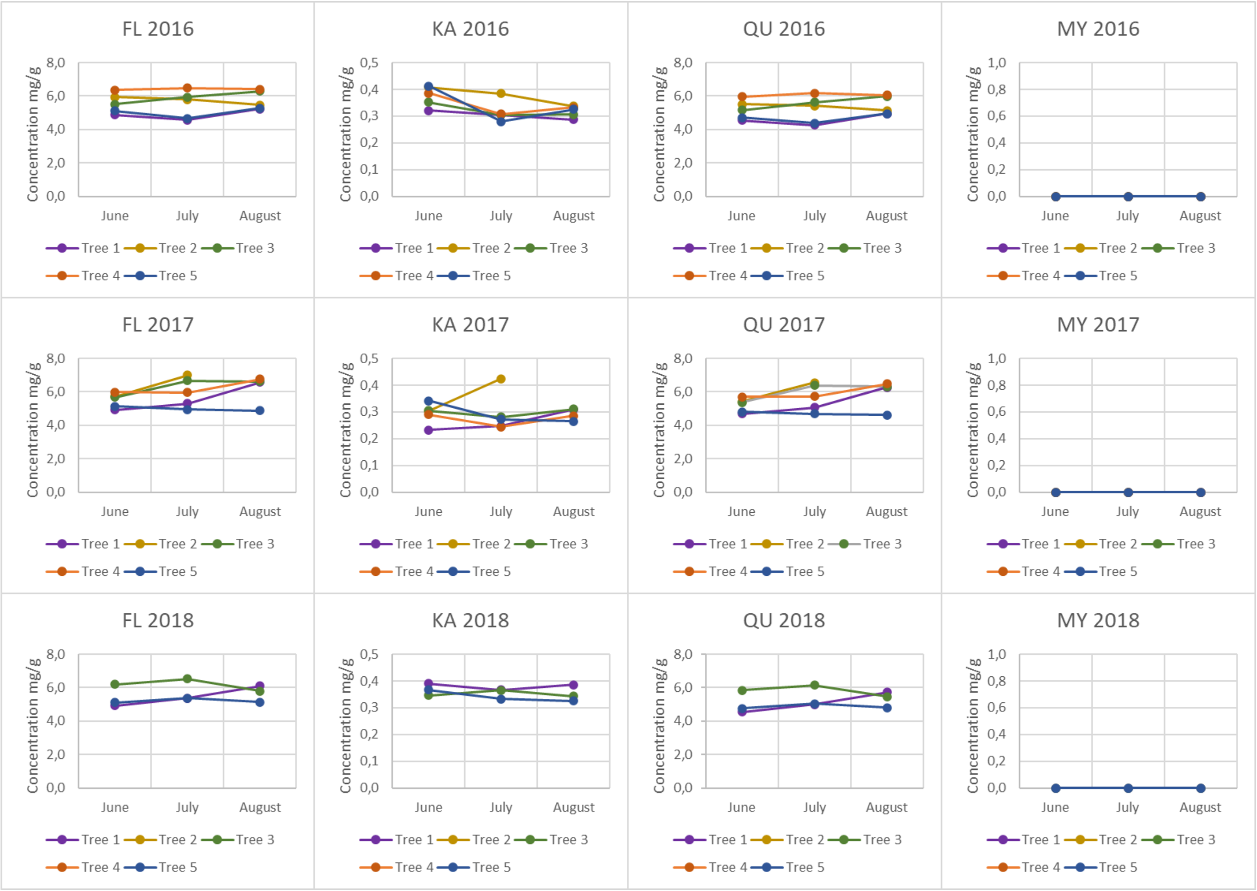

Figure S15C. The seasonal and yearly changes of flavonol glycosides (FL), kaempferol glycosides (KA), quercetin glycosides (QU) and myricetin glycosides (MY) of the three to five *Alnus incana* trees.

Table S6. Initial concentrations of the *Salix phylicifolia* population. Values are averages of the five individual trees ± standard error of the mean.

Salix phylicifolia		2016			2017			2018		
		June	July	August	June	July	August	June	July	August
Polyphenols	**TP**	81.6 ± 2.5	97.4 ± 3.3	87.3 ± 3.6	95.5 ± 2.3	128.7 ± 10.2	131.5 ± 18.1	108.2 ± 5.6	104.6 ± 5.0	89.7 ± 5.5
	**HT**	ND	ND	ND	ND	ND	ND	ND	ND	ND
	G	ND	ND	ND	ND	ND	ND	ND	ND	ND
	HHDP	ND	ND	ND	ND	ND	ND	ND	ND	ND
	**PA**	19.3 ± 1.1	28.1 ± 0.7	34.3 ± 1.8	20.1 ± 0.7	28.9 ± 0.5	28.5 ± 2.0	16.0 ± 1.6	24.3 ± 2.2	31.2 ± 2.7
	PC	3.3 ± 0.3	4.6 ± 0.4	5.1 ± 0.6	3.0 ± 0.3	4.1 ± 0.5	3.7 ± 0.4	2.6 ± 0.3	4.4 ± 0.7	4.1 ± 0.6
	PD	16.0 ± 0.9	23.5 ± 0.6	29.3 ± 1.7	17.0 ± 0.5	24.7 ± 0.7	24.8 ± 2.0	13.4 ± 1.3	19.9 ± 1.8	27.2 ± 2.4
	mDP	17.3 ± 0.6	16.4 ± 0.7	17.8 ± 1.2	17.0 ± 0.9	19.1 ± 1.7	20.3 ± 1.7	14.2 ± 0.6	11.9 ± 1	18.9 ± 1.6
	**FL**	5.7 ± 0.6	5.0 ± 0.5	5.0 ± 0.5	6.1 ± 0.8	5.5 ± 0.6	4.5 ± 0.6	4.7 ± 0.5	4.4 ± 0.6	3.5 ± 0.4
	KA	0.4 ± 0.0	0.5 ± 0.0	0.4 ± 0.0	0.3 ± 0.0	0.3 ± 0.0	0.3 ± 0.1	0.3 ± 0.0	0.3 ± 0.0	0.3 ± 0.0
	QU	1.8 ± 0.3	1.6 ± 0.3	1.6 ± 0.3	1.8 ± 0.4	1.6 ± 0.3	1.5 ± 0.3	1.5 ± 0.3	1.4 ± 0.3	1.1 ± 0.2
	MY	3.5 ± 0.3	3.0 ± 0.3	3.0 ± 0.3	4.0 ± 0.4	3.6 ± 0.4	2.7 ± 0.3	2.9 ± 0.3	2.7 ± 0.3	2.1 ± 0.2
	**QA**	0.9 ± 0.5	1.0 ± 0.3	1.0 ± 0.1	0.3 ± 0.1	0.6 ± 0.2	0.9 ± 0.2	0.4 ± 0.2	0.7 ± 0.2	0.7 ± 0.1
Activities	**OX**	26.7 ± 1.4	31.7 ± 1.3	30.1 ± 1.1	32.9 ± 1.2	45.7 ± 3.6	44.9 ± 7.3	37.6 ± 1.9	35.4 ± 1.2	29.9 ± 1.4
	**PPC**	6.0 ± 0.4	9.5 ± 0.3	12.9 ± 0.8	7.3 ± 0.2	8.5 ± 3.1	13.1 ± 1.8	5.3 ± 1.0	9.8 ± 1.4	10.6 ± 1.4

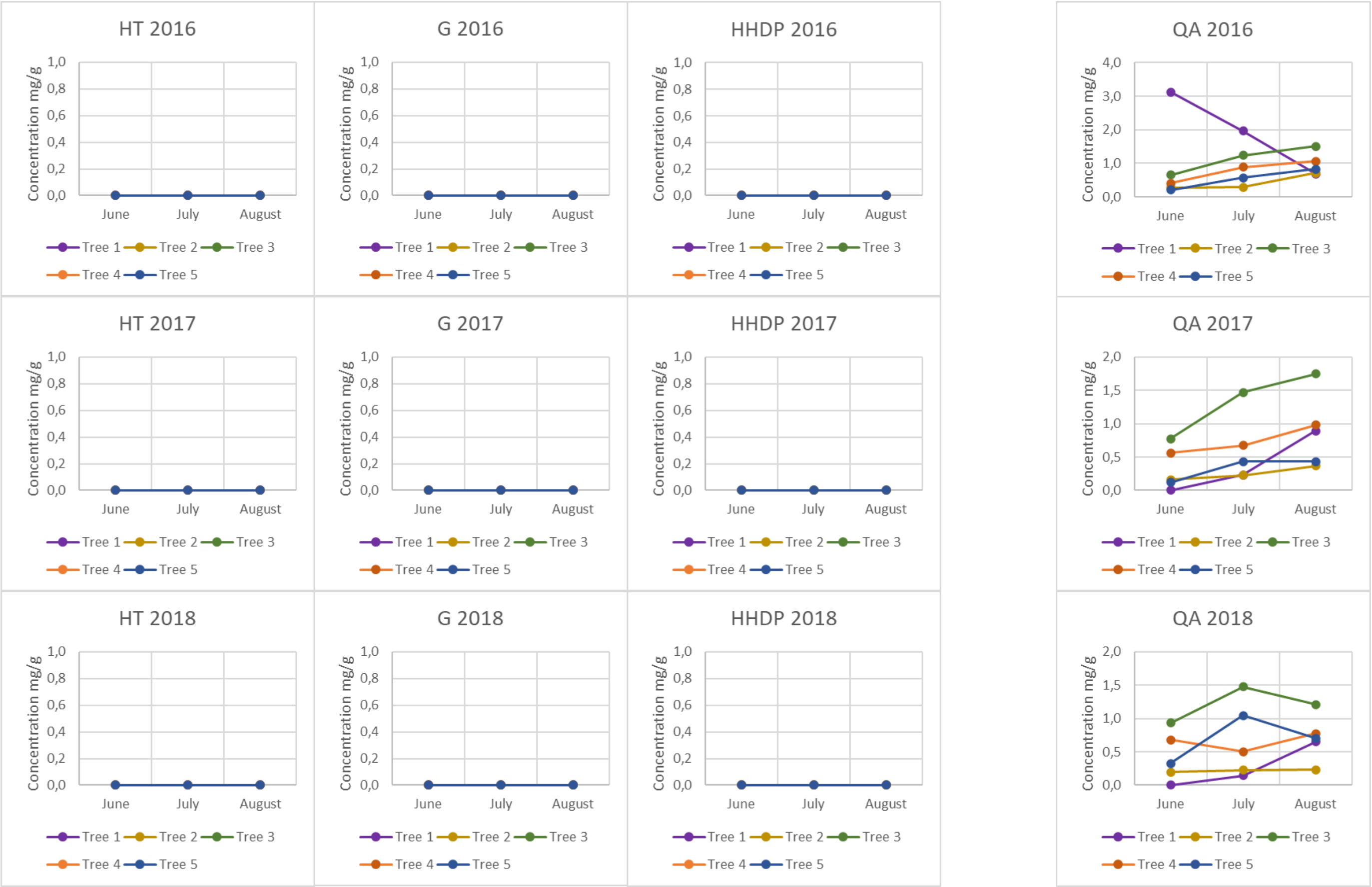

Figure S16A. The seasonal and yearly changes of hydrolysable tannins (HT), galloyl and hexahydroxydiphenoyl derivatives (G and HHDP) and quinic acid derivatives (QA) of the five *Salix phylicifolia* trees.

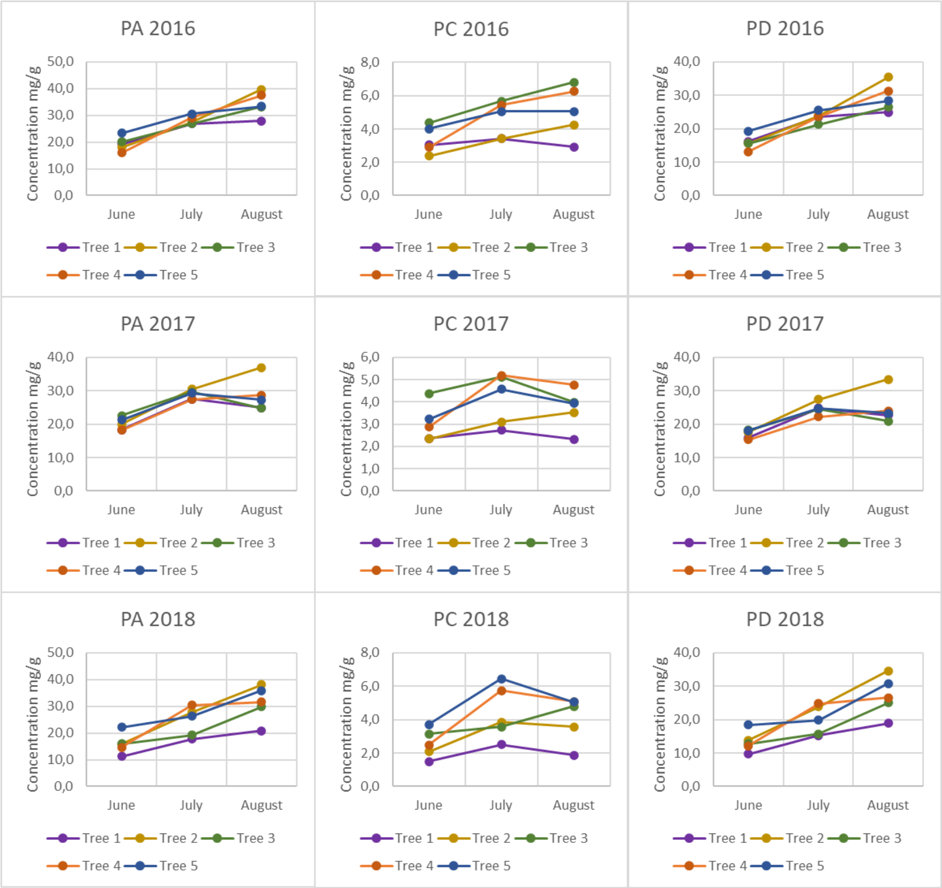

Figure S16B. The seasonal and yearly changes of proanthocyanidins (PA), procyanidins (PC) and prodelphinidins (PD) of the five *Salix phylicifolia* trees.

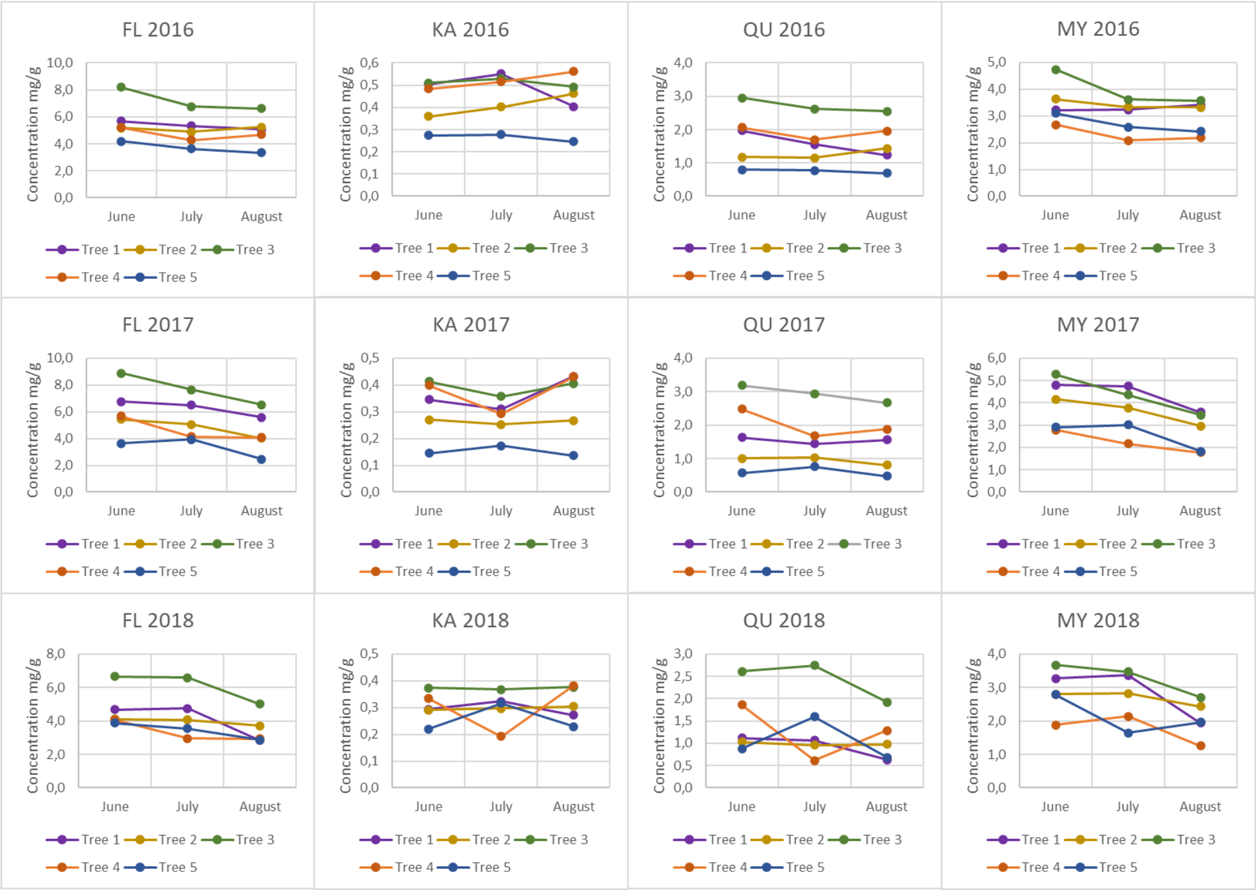

Figure S16C. The seasonal and yearly changes of flavonol glycosides (FL), kaempferol glycosides (KA), quercetin glycosides (QU) and myricetin glycosides (MY) of the five *Salix phylicifolia* trees.

Table S7. Initial concentrations of the *Sorbus aucuparia* population. Values are averages of the five individual trees ± standard error of the mean.

Sorbus aucuparia		2016			2017			2018		
		June	July	August	June	July	August	June	July	August
Polyphenols	**TP**	49.5 ± 2.0	36.1 ± 4.1	54.7 ± 2.6	60.3 ± 3.5	62.2 ± 4.8	66.5 ± 3.9	48.5 ± 3.4	58.8 ± 3.4	53.4 ± 4.7
	**HT**	ND	ND	ND	ND	ND	ND	ND	ND	ND
	G	ND	ND	ND	ND	ND	ND	ND	ND	ND
	HHDP	ND	ND	ND	ND	ND	ND	ND	ND	ND
	**PA**	22.7 ± 1.8	15.2 ± 2.2	25 ± 2.6	24.1 ± 3	26.4 ± 3.8	23.8 ± 3.0	17.3 ± 2.3	19.4 ± 2.4	19.4 ± 3.2
	PC	22.7 ± 1.8	15.2 ± 2.2	25 ± 2.6	24.1 ± 3	26.4 ± 3.8	23.8 ± 3.0	17.3 ± 2.3	19.4 ± 2.4	19.4 ± 3.2
	PD	ND	ND	ND	ND	ND	ND	ND	ND	ND
	mDP	6.6 ± 0.1	6.2 ± 0.2	5.4 ± 0.1	5.9 ± 0.2	4.9 ± 0.2	4.9 ± 0.2	5.5 ± 0.2	4.7 ± 0.1	5.0 ± 0.2
	**FL**	11.6 ± 0.9	8.0 ± 0.4	8.9 ± 0.5	11.5 ± 0.5	10.5 ± 0.4	10.3 ± 0.7	10.0 ± 0.5	11.6 ± 0.5	10.6 ± 0.8
	KA	4.3 ± 0.6	2.9 ± 0.5	3.3 ± 0.6	3.2 ± 0.5	3.3 ± 0.5	3.4 ± 0.6	3.5 ± 0.5	3.9 ± 0.6	4.0 ± 0.7
	QU	7.2 ± 0.3	5.0 ± 0.4	5.5 ± 0.2	8.1 ± 0.5	7.0 ± 0.2	6.7 ± 0.2	6.4 ± 0.3	7.4 ± 0.1	6.4 ± 0.4
	MY	0.2 ± 0.0	NS	0.2 ± 0.1	0.2 ± 0.1	0.2 ± 0.1	0.2 ± 0.1	0.1 ± 0.1	0.2 ± 0.1	0.3 ± 0.1
	**QA**	11.7 ± 0.3	6.5 ± 0.7	10.2 ± 0.2	11.4 ± 0.4	12.4 ± 0.7	12.8 ± 0.6	11.6 ± 0.3	12.9 ± 0.4	9.9 ± 0.8
Activities	**OX**	7.8 ± 1.1	5.4 ± 1.3	10.8 ± 0.8	10.2 ± 1.2	11.2 ± 0.8	14.2 ± 0.8	10.3 ± 1.3	16.5 ± 1.1	15.2 ± 2.6
	**PPC**	4.5 ± 1.3	1.8 ± 1.3	4.5 ± 1.1	6.5 ± 1.4	6.4 ± 1.7	4.6 ± 1.2	3.4 ± 1.2	5.1 ± 1.4	4.3 ± 1.8

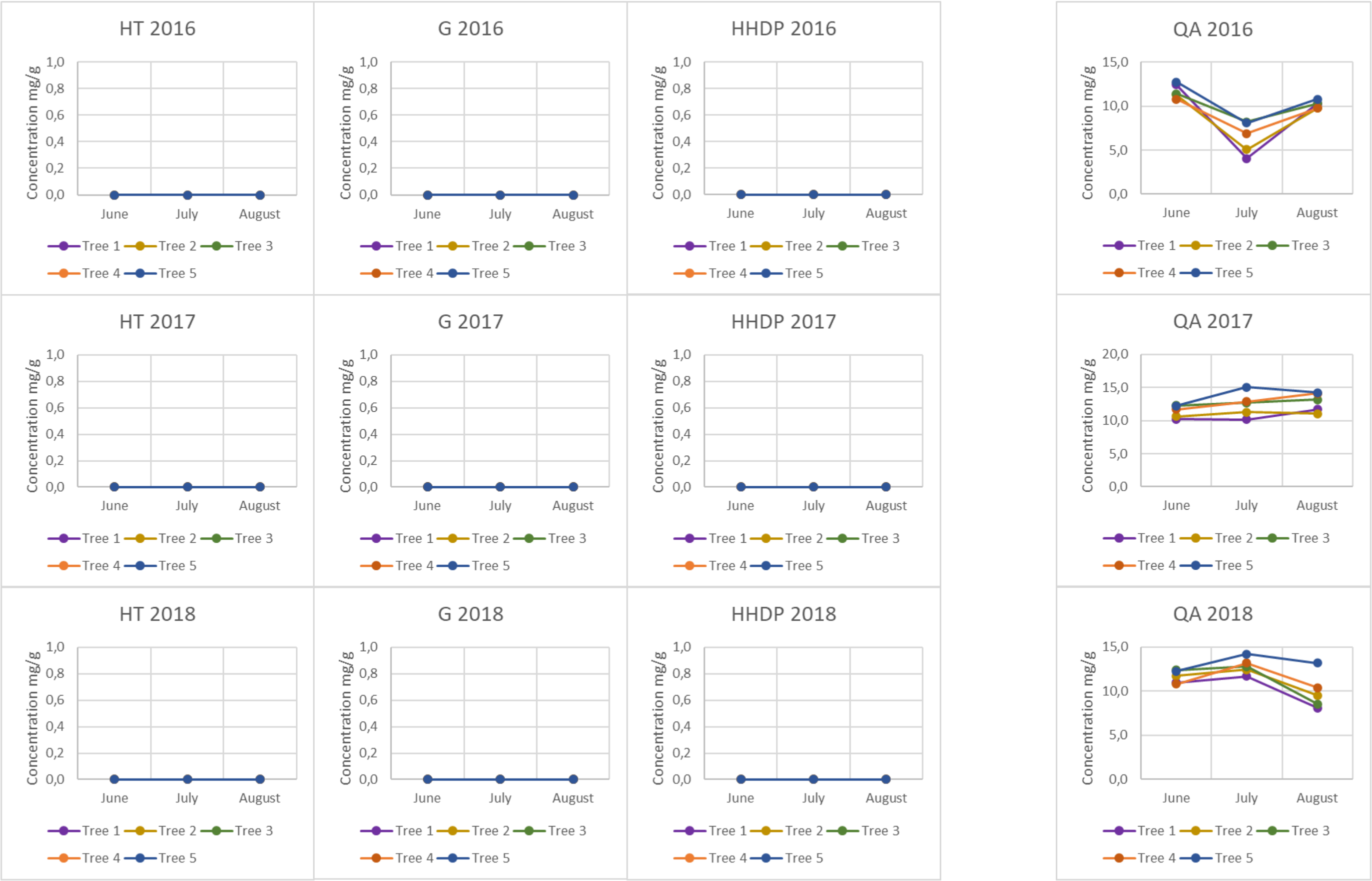

Figure S17A. The seasonal and yearly changes of hydrolysable tannins (HT), galloyl and hexahydroxydiphenoyl derivatives (G and HHDP) and quinic acid derivatives (QA) of the five *Sorbus aucuparia* trees.

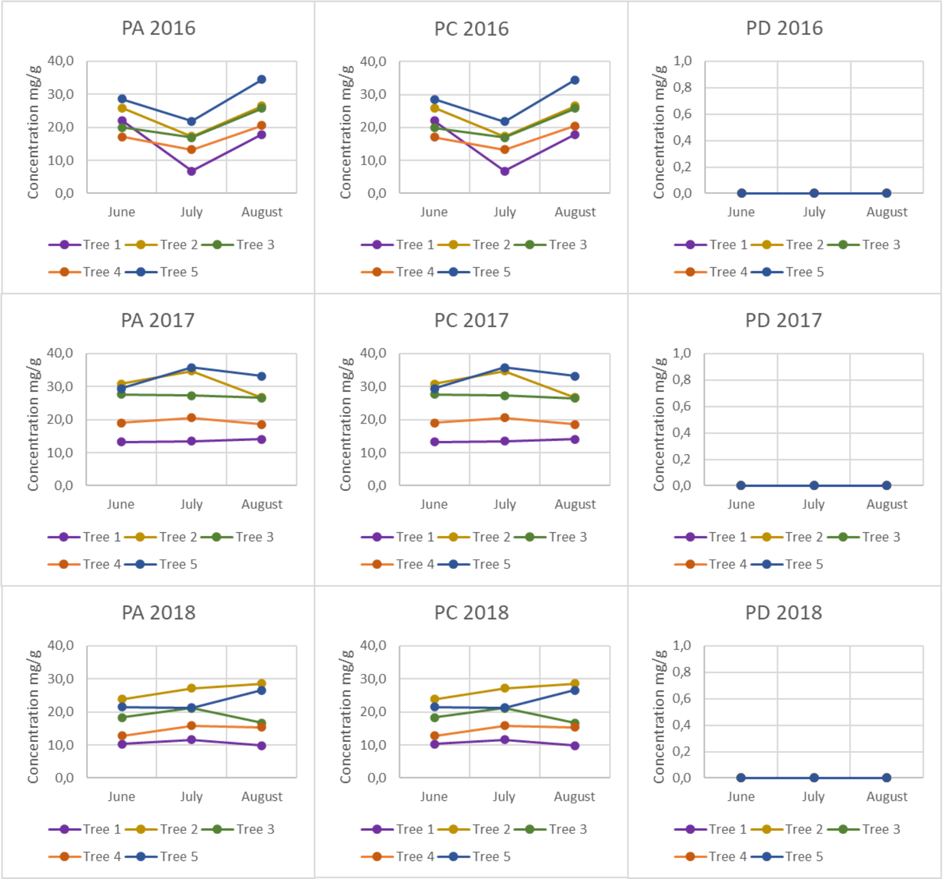

Figure S17B. The seasonal and yearly changes of proanthocyanidins (PA), procyanidins (PC) and prodelphinidins (PD) of the five *Sorbus aucuparia* trees.

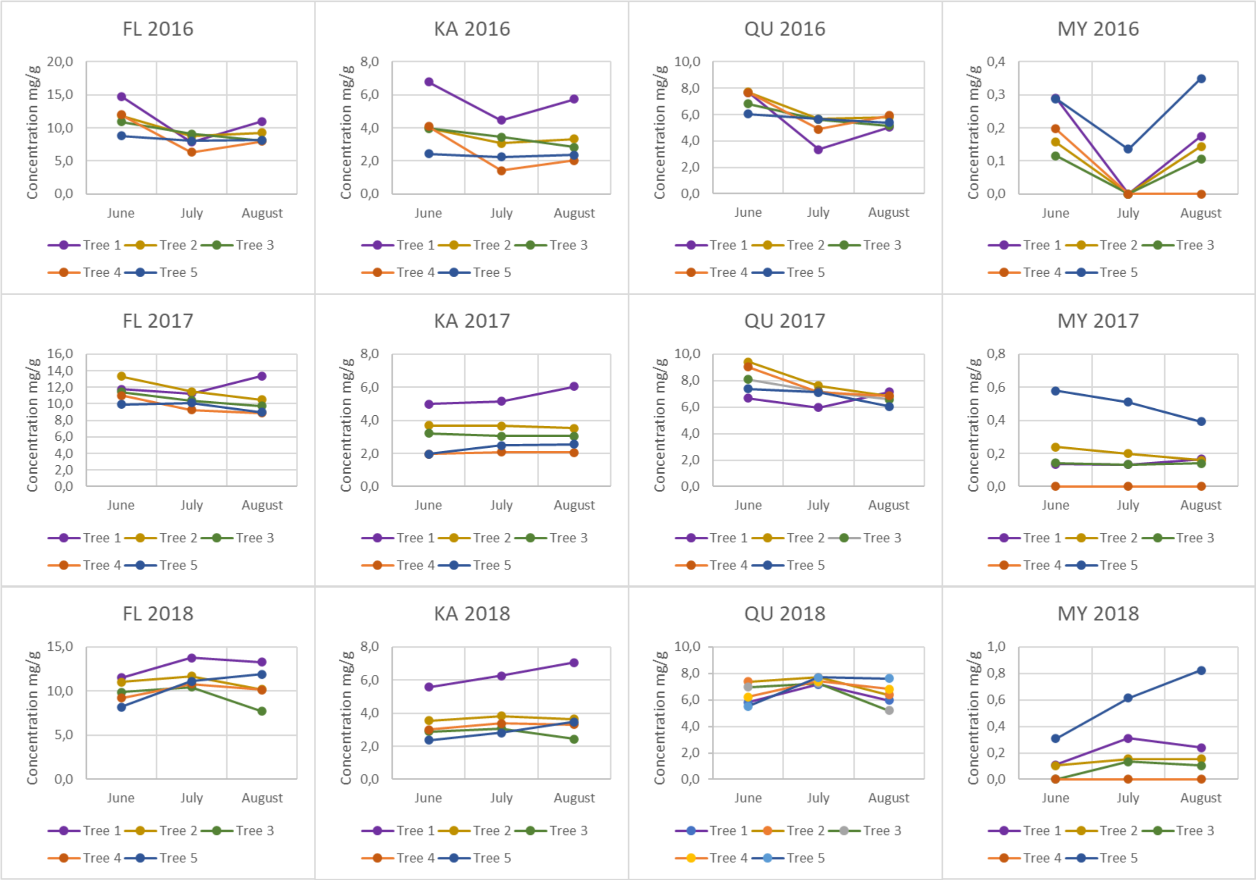

Figure S17C. The seasonal and yearly changes of flavonol glycosides (FL), kaempferol glycosides (KA), quercetin glycosides (QU) and myricetin glycosides (MY) of the five *Sorbus aucuparia* trees.

Table S8. Initial concentrations of the *Prunus padus* population. Values are averages of the five individual trees ± standard error of the mean.

Prunus padus		2016			2017			2018		
		June	July	August	June	July	August	June	July	August
Polyphenols	**TP**	28.6 ± 1.3	26.8 ± 1.4	30.5 ± 2.5	28.3 ± 1.3	29.6 ± 1.4	36.3 ± 2.3	27.1 ± 1.7	36.8 ± 3.6	35.9 ± 3.7
	**HT**	ND	ND	ND	ND	ND	ND	ND	ND	ND
	G	ND	ND	ND	ND	ND	ND	ND	ND	ND
	HHDP	ND	ND	ND	ND	ND	ND	ND	ND	ND
	**PA**	11.3 ± 1.4	11.9 ± 1.6	11.7 ± 2.6	9.2 ± 0.9	8.8 ± 1.3	12.8 ± 2.3	9.8 ± 1.4	18.5 ± 3.2	19.9 ± 3
	PC	11.3 ± 1.4	11.9 ± 1.6	11.7 ± 2.6	9.2 ± 0.9	8.8 ± 1.3	12.8 ± 2.3	9.8 ± 1.4	18.5 ± 3.2	19.9 ± 3
	PD	ND	ND	ND	ND	ND	ND	ND	ND	ND
	mDP	4.1 ± 0.1	3.9 ± 0.3	3.4 ± 0.3	3.2 ± 0.1	3.0 ± 0.1	3.1 ± 0.2	3.0 ± 0.2	3.3 ± 0.2	4.4 ± 0.2
	**FL**	7.1 ± 0.3	6.0 ± 0.4	6.0 ± 0.4	6.3 ± 0.4	5.9 ± 0.4	6.7 ± 0.2	6.0 ± 0.4	5.3 ± 0.3	4.3 ± 0.2
	KA	0.9 ± 0.1	0.8 ± 0.1	0.8 ± 0.1	0.7 ± 0.1	0.6 ± 0.1	0.8 ± 0.0	0.8 ± 0.0	0.7 ± 0.0	0.6 ± 0.1
	QU	6.2 ± 0.3	5.2 ± 0.4	5.2 ± 0.4	5.6 ± 0.3	5.2 ± 0.3	5.9 ± 0.2	5.2 ± 0.4	4.6 ± 0.2	3.7 ± 0.2
	MY	ND	ND	ND	ND	ND	ND	ND	ND	ND
	**QA**	6.4 ± 0.2	5.0 ± 0.3	3.6 ± 0.2	4.6 ± 0.2	4.8 ± 0.2	4.5 ± 0.2	4.1 ± 0.3	4.1 ± 0.2	2.6 ± 0.1
Activities	**OX**	1.5 ± 0.1	2.9 ± 1.0	3.6 ± 0.7	2.3 ± 0.3	3.3 ± 0.4	3.2 ± 0.9	1.8 ± 0.4	3.1 ± 0.6	2.0 ± 0.6
	**PPC**	0.2 ± 0.2	0.7 ± 0.3	0.2 ± 0.2	0.4 ± 0.3	NS	NS	NS	4.3 ± 1.5	1.6 ± 0.9

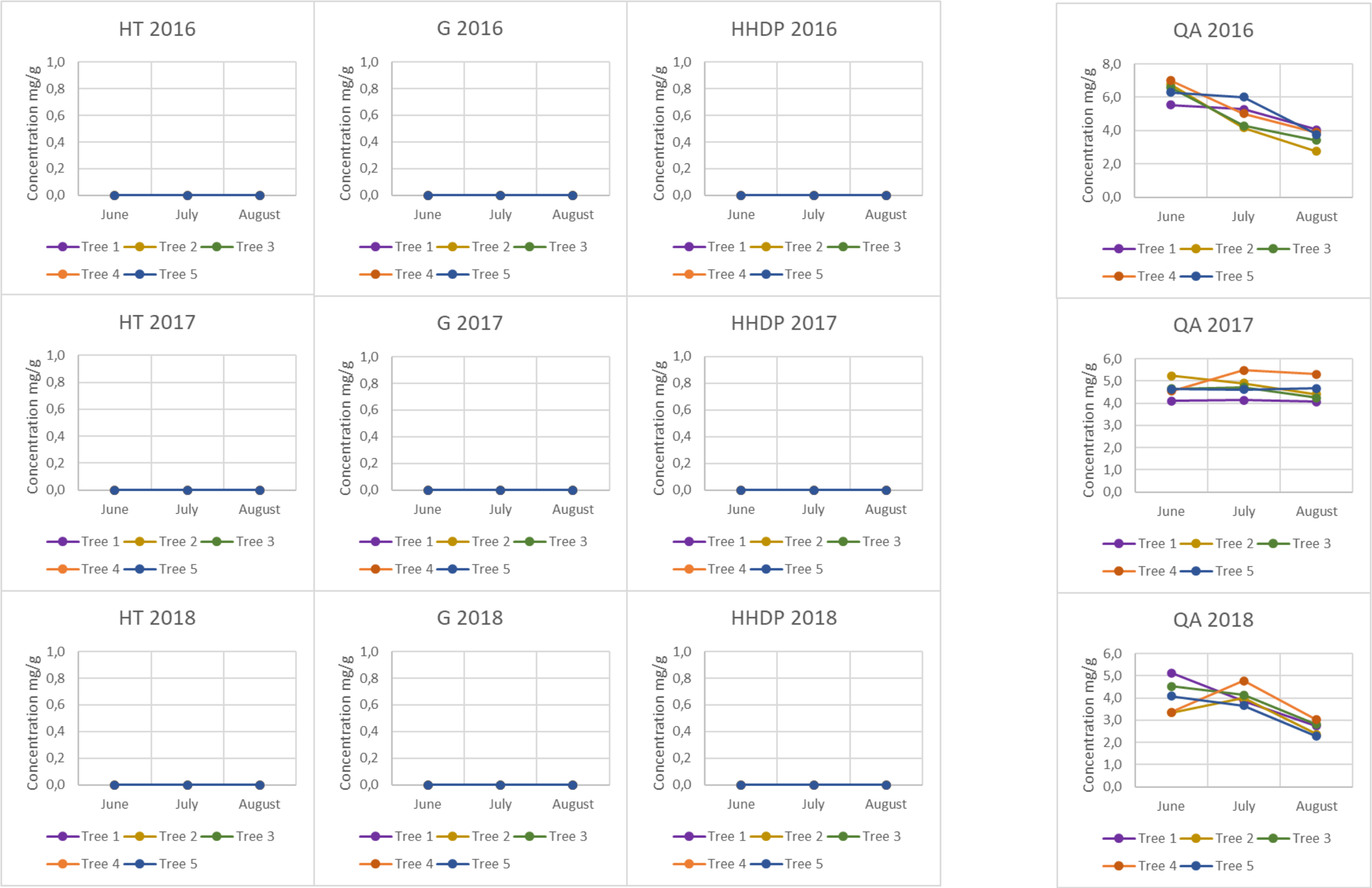

Figure S18A. The seasonal and yearly changes of hydrolysable tannins (HT), galloyl and hexahydroxydiphenoyl derivatives (G and HHDP) and quinic acid derivatives (QA) of the five *Prunus padus* trees.

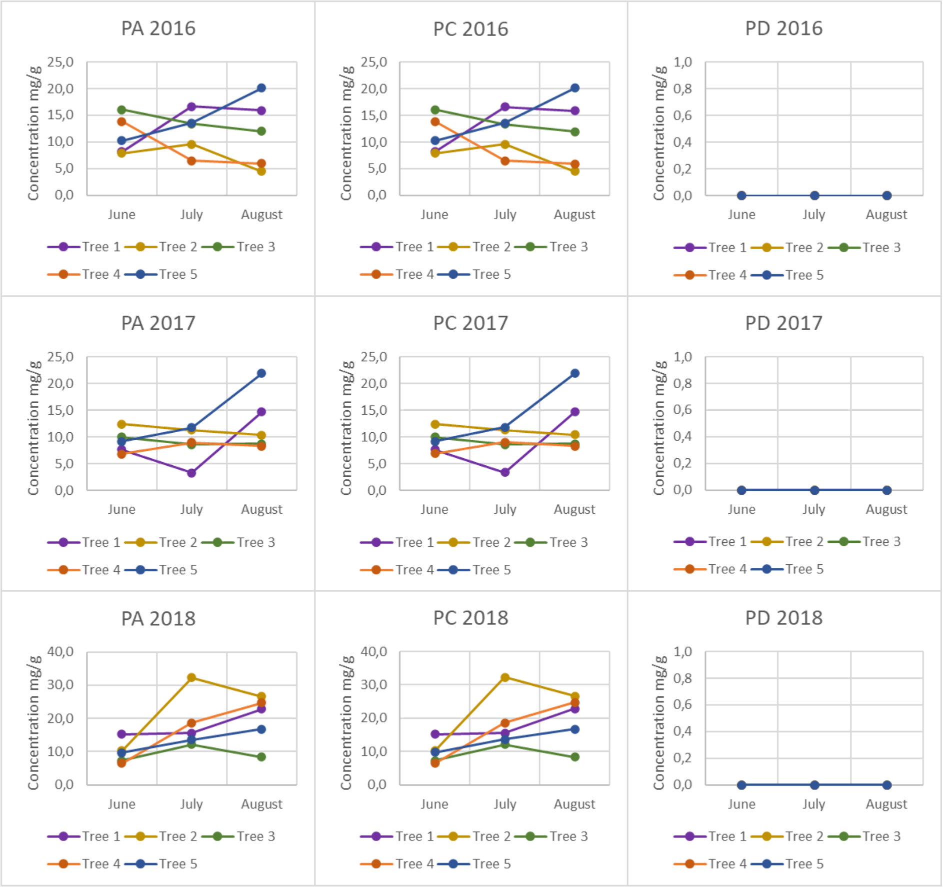

Figure S18B. The seasonal and yearly changes of proanthocyanidins (PA), procyanidins (PC) and prodelphinidins (PD) of the five *Prunus padus* trees.

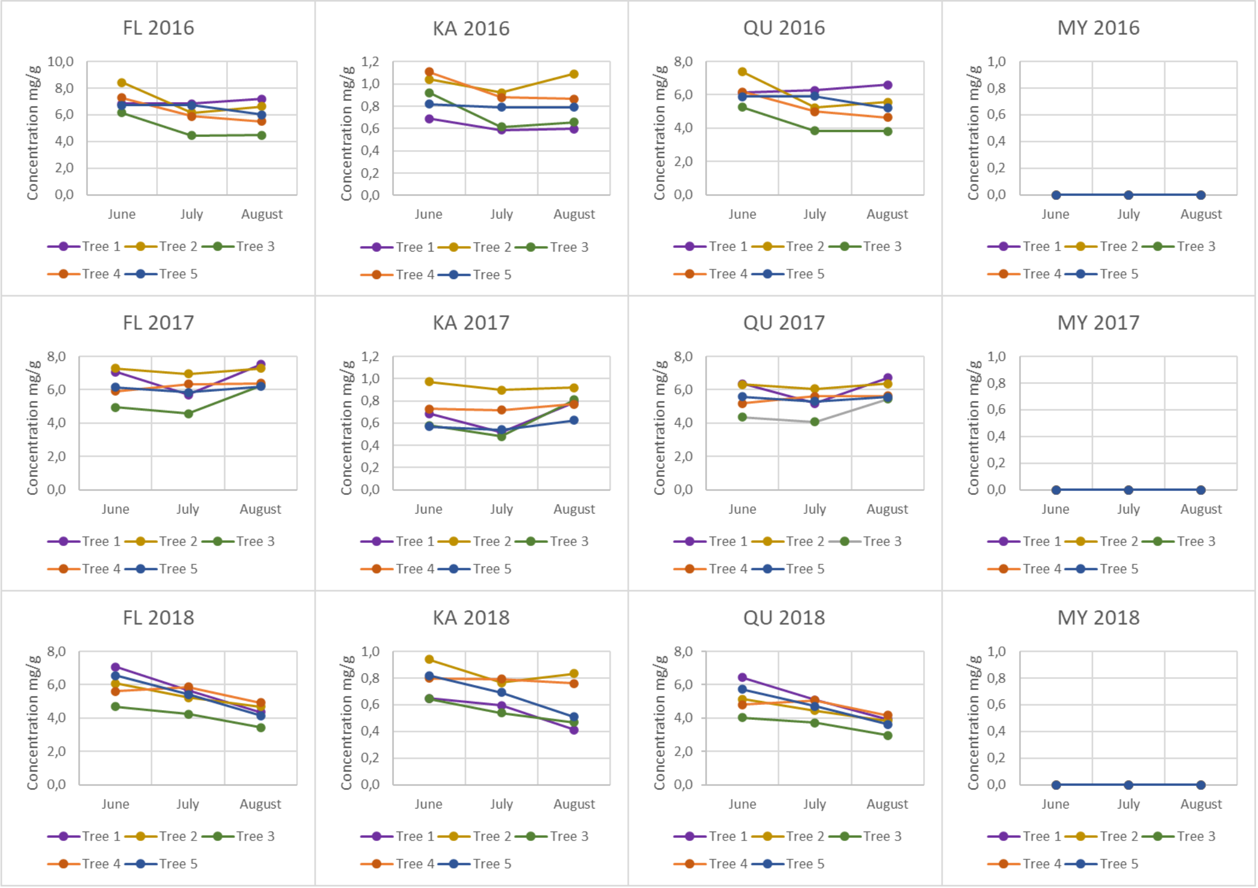

Figure S18C. The seasonal and yearly changes of flavonol glycosides (FL), kaempferol glycosides (KA), quercetin glycosides (QU) and myricetin glycosides (MY) of the five *Prunus padus* trees.

Table S9. Initial concentrations of the *Juniperus communis* population. Values are averages of the five individual trees ± standard error of the mean.

Juniperus communis		2016			2017			2018		
		June	July	August	June	July	August	June	July	August
Polyphenols	**TP**	41.6 ± 0.9	33.9 ± 1.3	46.7 ± 1.6	39.1 ± 0.6	46.2 ± 1	52.2 ± 1.3	45.5 ± 1.2	46.5 ± 1.9	46.8 ± 2.6
	**HT**	ND	ND	ND	ND	ND	ND	ND	ND	ND
	G	ND	ND	ND	ND	ND	ND	ND	ND	ND
	HHDP	ND	ND	ND	ND	ND	ND	ND	ND	ND
	**PA**	36.0 ± 1.8	30.4 ± 1.5	37.9 ± 1.6	29.1 ± 1.3	37.4 ± 1.1	34.7 ± 1.3	29.2 ± 1.3	30.8 ± 1.9	32.9 ± 1.7
	PC	32.6 ± 2.2	27.8 ± 1.6	34.0 ± 1.7	26.1 ± 1.5	32.9 ± 1	31.1 ± 1.1	25.6 ± 1.4	27.0 ± 1.9	30.4 ± 1.6
	PD	3.4 ± 0.6	2.5 ± 0.3	3.9 ± 0.4	3.0 ± 0.5	4.4 ± 0.4	3.6 ± 0.4	3.6 ± 0.4	3.8 ± 0.2	2.5 ± 0.3
	mDP	7.5 ± 0.3	6.0 ± 0.1	5.1 ± 0.1	6.9 ± 0.2	5.7 ± 0.2	5.1 ± 0.1	5.9 ± 0.2	4.8 ± 0.1	5.4 ± 0.2
	**FL**	4.5 ± 0.2	2.9 ± 0.1	2.7 ± 0.1	3.7 ± 0.1	2.9 ± 0.1	2.9 ± 0.1	4.0 ± 0.2	3.2 ± 0.1	2.7 ± 0.1
	KA	1.0 ± 0.1	0.6 ± 0.0	0.6 ± 0.0	0.6 ± 0.0	0.5 ± 0.0	0.6 ± 0.0	0.7 ± 0.1	0.5 ± 0.0	0.5 ± 0.0
	QU	3.3 ± 0.1	2.1 ± 0.1	2.1 ± 0.1	3.0 ± 0.1	2.3 ± 0.1	2.3 ± 0.1	3.2 ± 0.1	2.5 ± 0.1	2.1 ± 0.1
	MY	0.1 ± 0.1	0.1 ± 0.0	0.1 ± 0.0	0.1 ± 0.0	0.1 ± 0.0	0.1 ± 0.0	0.1 ± 0.0	0.1 ± 0.0	NS
	**QA**	0.9 ± 0.2	0.4 ± 0.1	0.2 ± 0.1	0.7 ± 0.2	0.3 ± 0.1	0.2 ± 0.1	0.7 ± 0.1	0.3 ± 0.1	0.1 ± 0.1
Activities	**OX**	4.9 ± 0.9	4.3 ± 0.5	6.4 ± 0.4	4.4 ± 0.7	6.0 ± 0.4	5.4 ± 0.4	7.5 ± 0.9	5.9 ± 0.3	5.9 ± 1.2
	**PPC**	8.1 ± 1.1	2.8 ± 0.9	5.7 ± 0.3	5.9 ± 1	6.6 ± 0.6	6.3 ± 0.8	6.5 ± 0.8	6.8 ± 0.7	4.6 ± 0.9

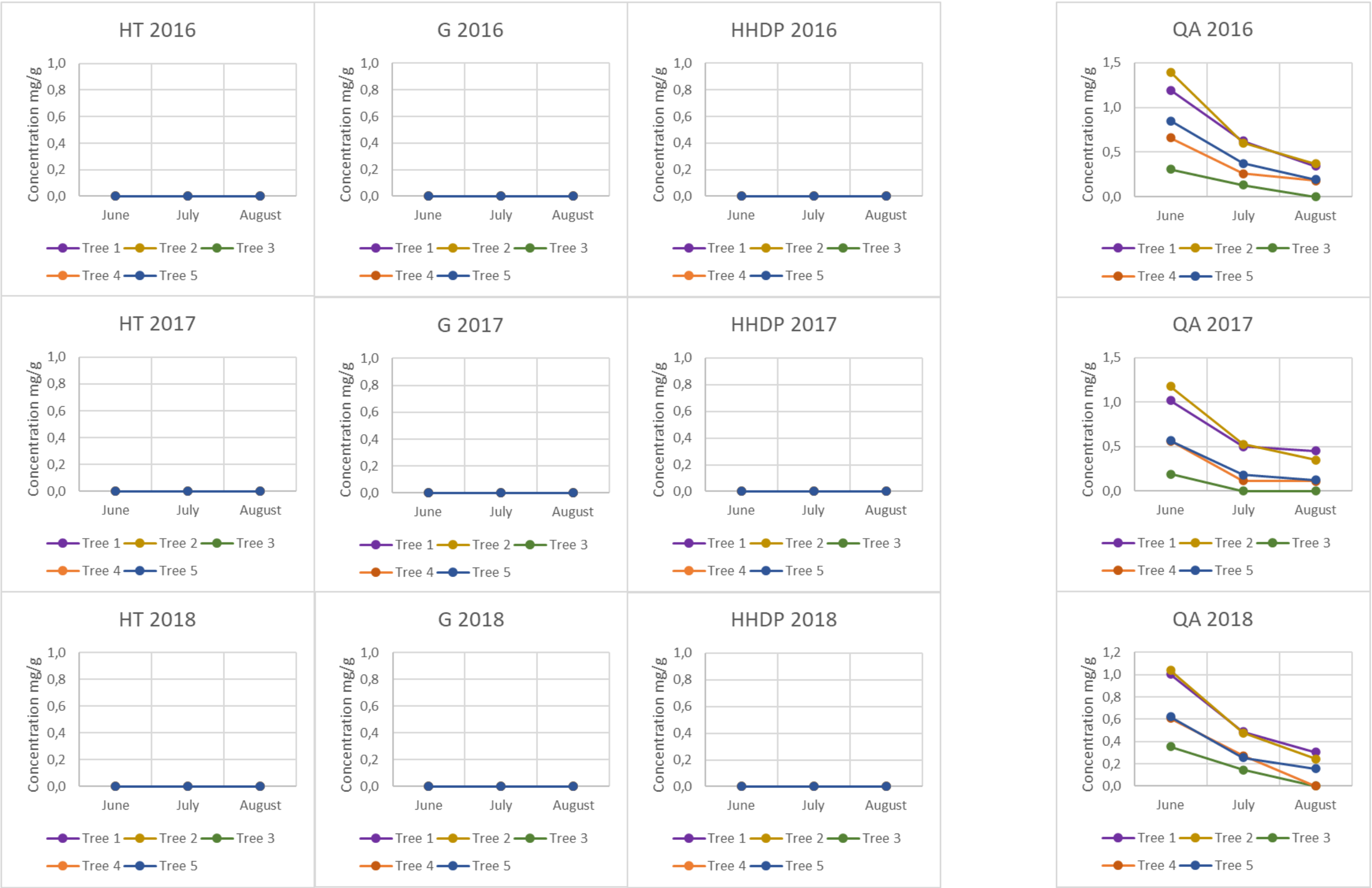

Figure S19A. The seasonal and yearly changes of hydrolysable tannins (HT), galloyl and hexahydroxydiphenoyl derivatives (G and HHDP) and quinic acid derivatives (QA) of the five *Juniperus communis* trees.

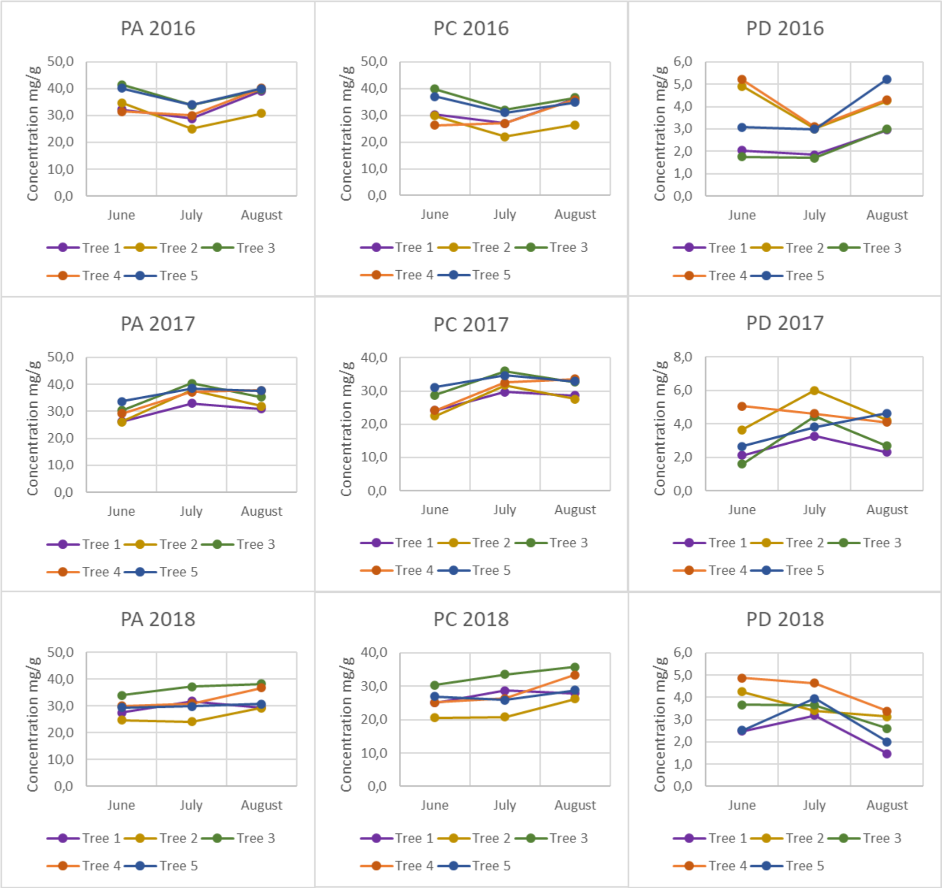

Figure S19B. The seasonal and yearly changes of proanthocyanidins (PA), procyanidins (PC) and prodelphinidins (PD) of the five *Juniperus communis* trees.

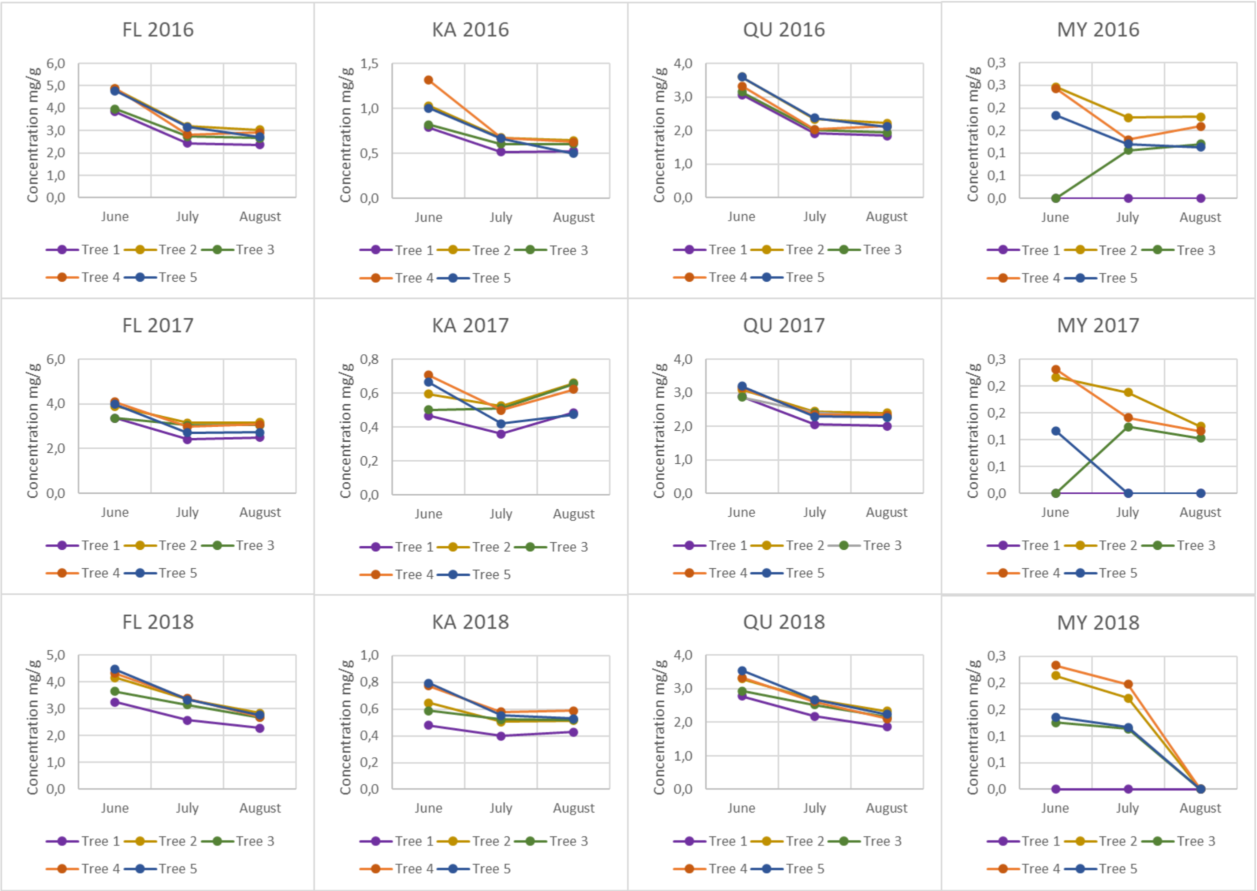

Figure S19C. The seasonal and yearly changes of flavonol glycosides (FL), kaempferol glycosides (KA), quercetin glycosides (QU) and myricetin glycosides (MY) of the five *Juniperus communis* trees.

Table S10. Initial concentrations of the *Picea abies* population. Values are averages of the five individual trees ± standard error of the mean.

Picea abies		2016			2017			2018		
		June	July	August	June	July	August	June	July	August
Polyphenols	**TP**	47.2 ± 2	31.1 ± 1.9	43.4 ± 2.2	42.1 ± 2.5	41.5 ± 2.6	55 ± 3.6	33.6 ± 3.2	46.2 ± 3	55 ± 4.2
	**HT**	ND	ND	ND	ND	ND	ND	ND	ND	ND
	G	ND	ND	ND	ND	ND	ND	ND	ND	ND
	HHDP	ND	ND	ND	ND	ND	ND	ND	ND	ND
	**PA**	36.9 ± 2.7	20.4 ± 2.5	29.5 ± 2.3	32.3 ± 2.7	28.1 ± 2.7	32.7 ± 3.3	22.2 ± 2.6	27.1 ± 2.7	37.5 ± 3.7
	PC	31.0 ± 4.1	18.4 ± 2.7	26.0 ± 2.5	27.4 ± 3.7	24.3 ± 3.1	28.1 ± 3.1	19.3 ± 2.9	23.7 ± 2.4	32.7 ± 3.7
	PD	5.8 ± 1.5	2.0 ± 0.4	3.5 ± 0.5	4.9 ± 1.7	3.8 ± 0.6	4.6 ± 0.8	3.0 ± 0.5	3.4 ± 0.7	4.8 ± 0.6
	mDP	8.7 ± 0.3	7.9 ± 0.3	6.3 ± 0.2	8.6 ± 0.5	7.3 ± 0.5	5.9 ± 0.4	6.0 ± 0.4	5.3 ± 0.4	6.3 ± 0.3
	**FL**	7.3 ± 0.2	1.4 ± 0.2	1.4 ± 0.1	5.0 ± 0.2	1.2 ± 0.1	1.5 ± 0.1	1.9 ± 0.2	1.2 ± 0.1	1.2 ± 0.1
	KA	5.2 ± 0.1	0.9 ± 0.1	1.0 ± 0.1	3.6 ± 0.2	0.8 ± 0.1	1.1 ± 0.1	1.1 ± 0.1	0.8 ± 0.1	0.9 ± 0.1
	QU	1.5 ± 0.2	0.3 ± 0.1	0.2 ± 0.0	1.0 ± 0.1	0.2 ± 0.0	0.2 ± 0.0	0.5 ± 0.1	0.2 ± 0.0	0.2 ± 0.0
	MY	0.6 ± 0.1	0.2 ± 0.0	0.2 ± 0.0	0.5 ± 0.1	0.2 ± 0.0	0.1 ± 0.0	0.3 ± 0.0	0.2 ± 0.0	0.1 ± 0.0
	**QA**	1 ± 0.0	0.4 ± 0.1	0.3 ± 0.1	0.6 ± 0.1	0.3 ± 0.1	0.3 ± 0.1	0.6 ± 0.1	0.4 ± 0.1	0.3 ± 0.1
Activities	**OX**	6.4 ± 1.6	6.7 ± 0.8	10.5 ± 0.7	7.1 ± 2.2	9 ± 0.5	13.8 ± 0.6	7.0 ± 2.2	13.1 ± 0.9	17.7 ± 1.3
	**PPC**	12.2 ± 0.9	1.2 ± 0.7	5.3 ± 0.9	8.7 ± 0.7	3.4 ± 1	4.2 ± 0.9	3.9 ± 1	5.2 ± 0.9	7.2 ± 1.5

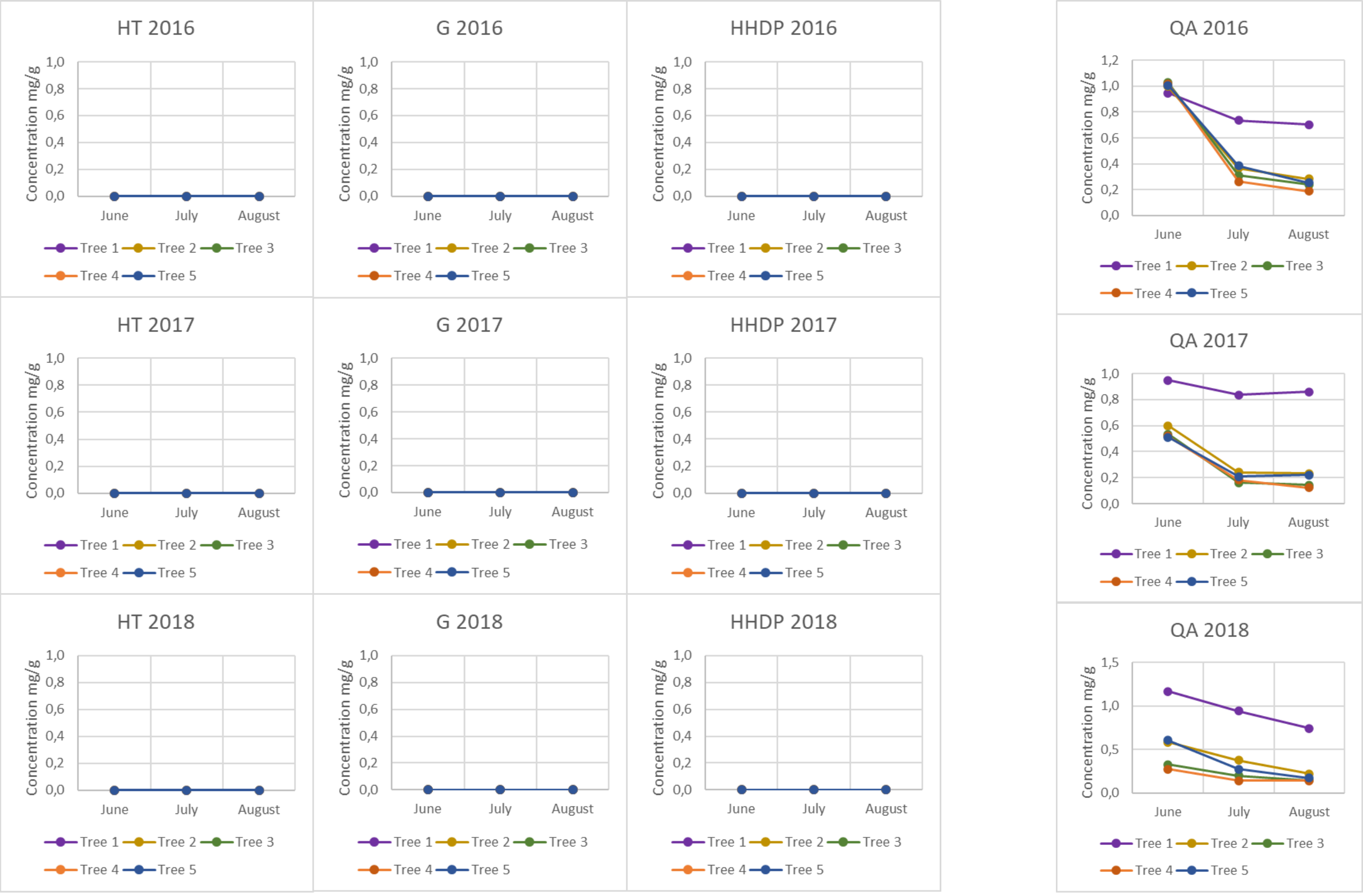

Figure S20A. The seasonal and yearly changes of hydrolysable tannins (HT), galloyl and hexahydroxydiphenoyl derivatives (G and HHDP) and quinic acid derivatives (QA) of the five *Picea abies* trees.

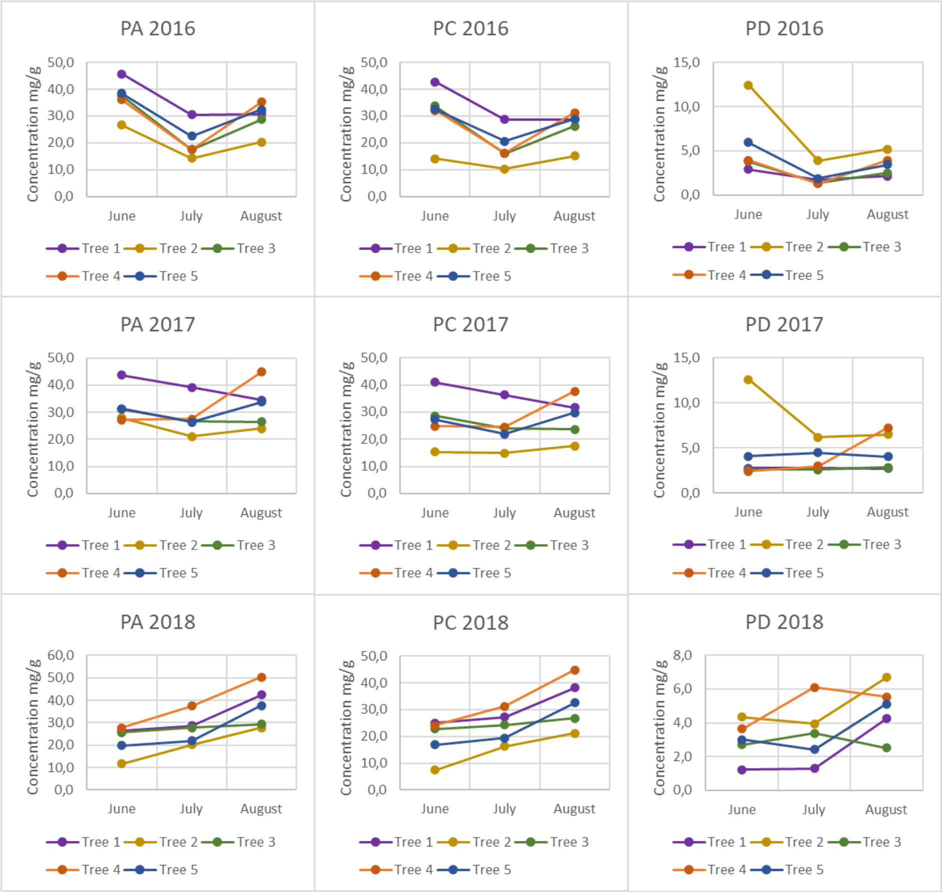

Figure S20B. The seasonal and yearly changes of proanthocyanidins (PA), procyanidins (PC) and prodelphinidins (PD) of the five *Picea abies* trees.

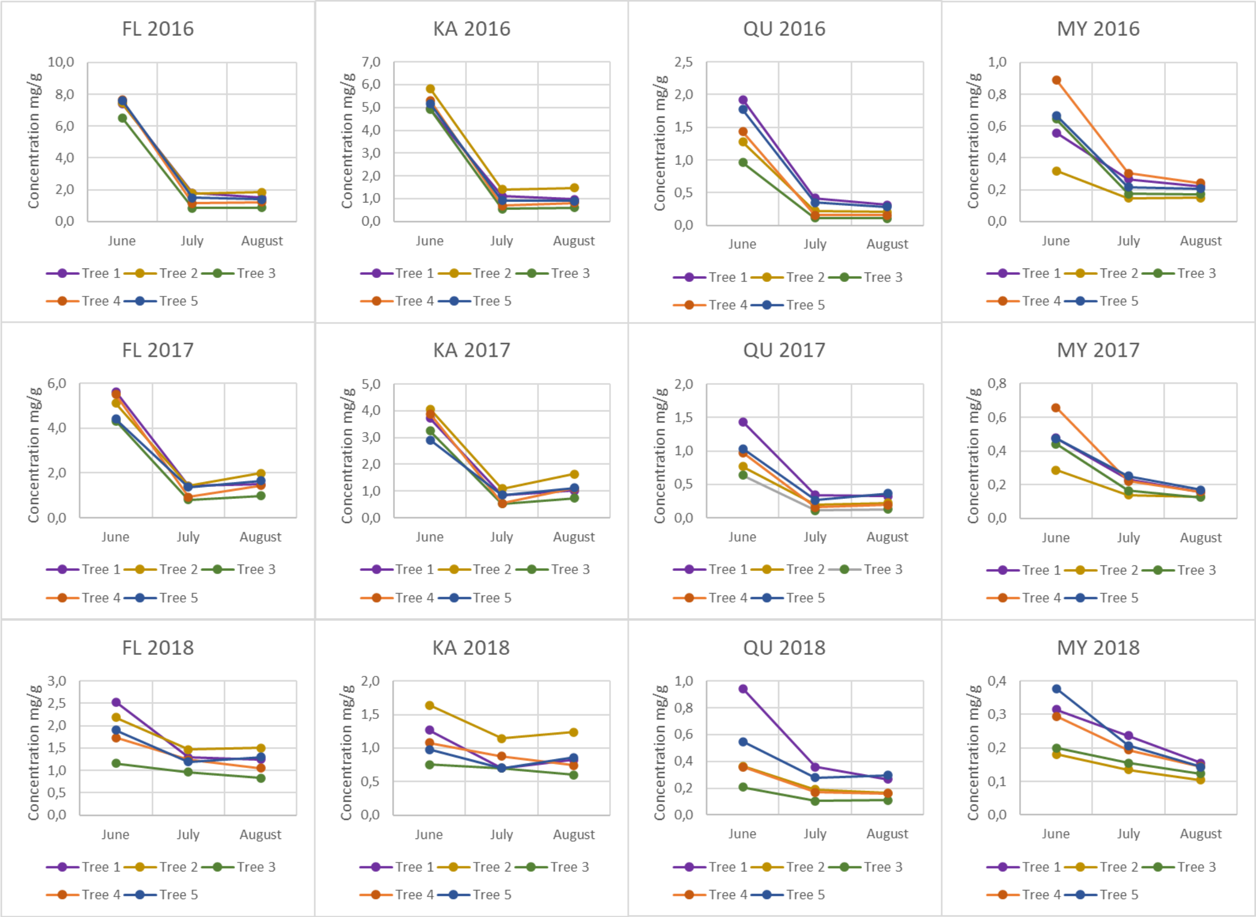

Figure S20C. The seasonal and yearly changes of flavonol glycosides (FL), kaempferol glycosides (KA), quercetin glycosides (QU) and myricetin glycosides (MY) of the five *Picea abies* trees.

Table S11. Coordinates of the plant populations and collection dates of the 1st, 2nd, and 3rd collection.

			2016			2017			2018		
**Species**	**Coordinates**		**1st**	**2st**	**3rd**	**1st**	**2st**	**3rd**	**1st**	**2st**	**3rd**
Juniperus communis	N 60° 27.505'	E 22° 18.773'	31.5.	4.7.	17.8.	19.6.	3.8.	5.9.	2.6.	5.7.	8.8.
Picea abies	N 60° 28.060'	E 22° 19.739'	27.5.	30.6.	17.8.	16.6.	1.8.	5.9.	5.6.	5.7.	8.8.
Sorbus aucuparia	N 60° 27.512'	E 22° 18.659'	31.5.	4.7.	17.8.	19.6.	3.8.	5.9.	2.6.	5.7.	8.8.
Prunus padus	N 60° 27.710'	E 22° 16.921'	2.6.	6.7.	19.8.	16.6.	3.8.	6.9.	1.6.	6.7.	13.8.
Betula pubescens	N 60° 26.083'	E 22° 10.397'	25.5.	29.6.	15.8.	14.6.	1.8.	5.9.	4.6.	5.7.	8.8.
Alnus glutinosa	N 60° 26.238'	E 22° 12.247'	30.5.	5.7.	18.8.	14.6.	1.8.	5.9.	4.6.	5.7.	8.8.
Alnus incana	N 60° 27.872'	E 22° 19.274'	27.5.	30.6.	17.8.	16.6.	1.8.	5.9.	5.6.	5.7.	8.8.
Quercus robur	N 60° 26.200'	E 22° 10.280'	30.5.	5.7.	18.8.	14.6.	1.8.	5.9.	4.6.	5.7.	8.8.
Salix phylicifolia	N 60° 27.576'	E 22° 22.473'	3.6.	7.7.	18.8.	19.6.	3.8.	5.9.	2.6.	5.7.	8.8.
Acer platanoides	N 60° 27.444'	E 22° 16.679'	2.6.	6.7.	19.8.	16.6.	3.8.	6.9.	1.6.	6.7.	13.8.

Table S12. The initial concentration values (mg/g) of each polyphenol group and bioactivity at normalized scale (0.0-1.0), detection limit (0.0) and detected maximal values (max).

	**0.0**	**0.2**	**0.4**	**0.6**	**0.8**	**1.0**	**max**
Gallic acid derivatives	0.1	5.0	10.0	15.0	20.0	25.0	98.6
HHDP derivatives	0.1	15.0	30.0	45.0	60.0	75.0	213.6
**Total hydrolysable tannins**	0.1	17.0	34.0	51.0	68.0	85.0	244.9
Procyanidins	1.0	15.0	30.0	45.0	60.0	75.0	307.4
Prodelphinidins	1.0	9.0	18.0	27.0	36.0	45.0	212.2
**Total proanthocyanidins**	1.0	17.0	34.0	51.0	68.0	85.0	309.0
Kaempferol derivatives	0.1	1.2	2.4	3.6	4.8	6.0	40.2
Quercetin derivatives	0.1	1.7	3.4	5.1	6.8	8.5	24.9
Myricetin derivatives	0.1	1.2	2.4	3.6	4.8	6.0	25.1
**Total flavonol derivatives**	0.1	2.5	5.0	7.5	10.0	12.5	49.2
**Quinic acid derivatives**	0.1	2.6	5.2	7.8	10.4	13.0	41.4
Oxidative activity	0.1	8.0	16.0	24.0	32.0	40.0	129.4
Protein precipitation capacity	0.1	14.0	28.0	42.0	56.0	70.0	188.4

Table S13. The initial concentration values (mg/g) of each flavonol group and glycosylation pattern at normalized scale.

	**0.0**	**0.2**	**0.4**	**0.6**	**0.8**	**1.0**	**max**
KA-3-*O*-glycosides	0.10	1.20	2.40	3.60	4.80	6.00	39.74
Other KA-glycosides	0.10	0.15	0.20	0.25	0.30	0.35	2.92
**Total kaempferol derivatives**	0.10	1.20	2.40	3.60	4.80	6.00	40.20
QU-3-*O*-glycosides	0.10	1.20	2.40	3.60	4.80	6.00	19.48
Other QU-glycosides	0.10	0.60	1.20	1.80	2.40	3.00	15.89
**Total quercetin derivatives**	0.10	1.70	3.40	5.10	6.80	8.50	24.90
MY-3-*O*-glycosides	0.10	0.80	1.60	2.40	3.20	4.00	23.11
Other MY-glycosides	0.10	0.15	0.20	0.25	0.30	0.35	2.03
**Total myricetin derivatives**	0.10	1.20	2.40	3.60	4.80	6.00	25.10

Table S14. The results of statistical analyses of *Betula pubescens*. The differences in the mean values of quantitative results of each polyphenol group between individual trees, years and sampling times were analyzed using three-way analysis of variance (Three-way ANOVA). Pairwise multiple comparison procedures were performed with Holm-Sidak method, when significant differences were detected. The significance level was set to p=0.05.

Dependent Variable: **GALL_TOT**
Normality Test (Shapiro-Wilk): Failed (P < 0,050)
Equal Variance Test (Brown-Forsythe): Passed (P = 1,000)

Source of Variation	DF	SS	MS	F	P
individual	4	35,868	8,967	15,226	<0,001
month	2	21,061	10,531	17,881	<0,001
year	2	12,005	6,002	10,192	<0,001
Residual	36	21,201	0,589		
Total	44	90,135	2,049		

Comparisons for factor: **individual**

Comparison	Diff of Means	t	P	P<0,050
1,000 vs. 5,000	2,378	6,572	<0,001	Yes
3,000 vs. 5,000	2,315	6,399	<0,001	Yes
4,000 vs. 5,000	2,144	5,926	<0,001	Yes
2,000 vs. 5,000	1,432	3,958	0,002	Yes
1,000 vs. 2,000	0,946	2,614	0,075	No
3,000 vs. 2,000	0,883	2,440	0,095	No
4,000 vs. 2,000	0,712	1,968	0,208	No
1,000 vs. 4,000	0,234	0,646	0,891	No
3,000 vs. 4,000	0,171	0,472	0,891	No
1,000 vs. 3,000	0,0629	0,174	0,891	No

Comparisons for factor: **month**

Comparison	Diff of Means	t	P	P<0,050
june vs. august	1,594	5,688	<0,001	Yes
june vs. july	1,245	4,443	<0,001	Yes
july vs. august	0,349	1,245	0,221	No

Comparisons for factor: **year**

Comparison	Diff of Means	t	P	P<0,050
2016,000 vs. 2018,000	1,166	4,160	<0,001	Yes
2017,000 vs. 2018,000	1,009	3,600	0,002	Yes
2016,000 vs. 2017,000	0,157	0,560	0,579	No

Dependent Variable: **HHDP_TOT**
Normality Test (Shapiro-Wilk): Passed (P = 0,158)
Equal Variance Test (Brown-Forsythe): Passed (P = 1,000)

Source of Variation	DF	SS	MS	F	P
individual	4	308,677	77,169	7,352	<0,001
month	2	477,766	238,883	22,759	<0,001
year	2	78,867	39,434	3,757	0,033
Residual	36	377,866	10,496		
Total	44	1243,176	28,254		

Comparisons for factor: **individual**

Comparison	Diff of Means	t	P	P<0,050
4,000 vs. 5,000	7,738	5,067	<0,001	Yes
1,000 vs. 5,000	5,929	3,882	0,004	Yes
3,000 vs. 5,000	5,396	3,533	0,009	Yes
4,000 vs. 2,000	4,090	2,678	0,075	No
2,000 vs. 5,000	3,648	2,389	0,126	No
4,000 vs. 3,000	2,342	1,533	0,513	No
1,000 vs. 2,000	2,281	1,493	0,513	No
4,000 vs. 1,000	1,809	1,184	0,568	No
3,000 vs. 2,000	1,748	1,144	0,568	No
1,000 vs. 3,000	0,533	0,349	0,729	No

Comparisons for factor: **month**

Comparison	Diff of Means	t	P	P<0,050
june vs. august	7,698	6,507	<0,001	Yes
june vs. july	5,674	4,797	<0,001	Yes
july vs. august	2,024	1,711	0,096	No

Comparisons for factor: **year**

Comparison	Diff of Means	t	P	P<0,050
2016,000 vs. 2018,000	3,184	2,691	0,032	Yes
2017,000 vs. 2018,000	2,126	1,797	0,155	No
2016,000 vs. 2017,000	1,058	0,894	0,377	No

Dependent Variable: **PC_TOT**
Normality Test (Shapiro-Wilk): Passed (P = 0,085)
Equal Variance Test (Brown-Forsythe): Passed (P = 1,000)

Source of Variation	DF	SS	MS	F	P
individual	4	63,496	15,874	7,997	<0,001
month	2	76,157	38,079	19,183	<0,001
year	2	46,692	23,346	11,761	<0,001
Residual	36	71,461	1,985		
Total	44	257,806	5,859		

Comparisons for factor: **individual**

Comparison	Diff of Means	t	P	P<0,050
1,000 vs. 3,000	3,259	4,906	<0,001	Yes
1,000 vs. 4,000	2,592	3,903	0,004	Yes
5,000 vs. 3,000	2,405	3,620	0,007	Yes
2,000 vs. 3,000	2,114	3,183	0,021	Yes
5,000 vs. 4,000	1,738	2,617	0,075	No
2,000 vs. 4,000	1,448	2,180	0,167	No
1,000 vs. 2,000	1,144	1,723	0,325	No
1,000 vs. 5,000	0,854	1,286	0,501	No
4,000 vs. 3,000	0,666	1,003	0,541	No
5,000 vs. 2,000	0,290	0,437	0,665	No

Comparisons for factor: **month**

Comparison	Diff of Means	t	P	P<0,050
august vs. june	3,092	6,011	<0,001	Yes
july vs. june	2,212	4,299	<0,001	Yes
august vs. july	0,881	1,712	0,096	No

Comparisons for factor: **year**

Comparison	Diff of Means	t	P	P<0,050
2016,000 vs. 2017,000	2,296	4,464	<0,001	Yes
2016,000 vs. 2018,000	1,993	3,874	<0,001	Yes
2018,000 vs. 2017,000	0,303	0,589	0,559	No

Dependent Variable: **PD_TOT**
Normality Test (Shapiro-Wilk): Failed (P < 0,050)
Equal Variance Test (Brown-Forsythe): Passed (P = 1,000)

Source of Variation	DF	SS	MS	F	P
individual	4	327,745	81,936	12,036	<0,001
month	2	60,056	30,028	4,411	0,019
year	2	36,154	18,077	2,656	0,084
Residual	36	245,065	6,807		
Total	44	669,021	15,205		

Comparisons for factor: **individual**

Comparison	Diff of Means	t	P	P<0,050
1,000 vs. 3,000	7,502	6,100	<0,001	Yes
4,000 vs. 3,000	6,086	4,948	<0,001	Yes
1,000 vs. 2,000	5,313	4,320	<0,001	Yes
5,000 vs. 3,000	4,760	3,870	0,003	Yes
4,000 vs. 2,000	3,897	3,168	0,019	Yes
1,000 vs. 5,000	2,742	2,230	0,151	No
5,000 vs. 2,000	2,571	2,090	0,164	No
2,000 vs. 3,000	2,189	1,780	0,230	No
1,000 vs. 4,000	1,416	1,151	0,448	No
4,000 vs. 5,000	1,326	1,078	0,448	No

Comparisons for factor: **month**

Comparison	Diff of Means	t	P	P<0,050
august vs. june	2,814	2,953	0,016	Yes
july vs. june	1,668	1,751	0,169	No
august vs. july	1,145	1,202	0,237	No

Comparisons for factor: **year**

Comparison	Diff of Means	t	P	P<0,050
2016,000 vs. 2018,000	2,175	2,283	0,083	No
2017,000 vs. 2018,000	1,348	1,415	0,304	No
2016,000 vs. 2017,000	0,827	0,868	0,391	No

Dependent Variable: **QUIN_TOT**

Normality Test (Shapiro-Wilk): Failed (P < 0,050)

Equal Variance Test (Brown-Forsythe): Passed (P = 1,000)

Source of Variation	DF	SS	MS	F	P
individual	4	37,010	9,252	11,110	<0,001
month	2	69,955	34,977	42,000	<0,001
year	2	6,710	3,355	4,029	0,026
Residual	36	29,981	0,833		
Total	44	143,655	3,265		

Comparisons for factor: **individual**

Comparison	Diff of Means	t	P	P<0,050
4,000 vs. 3,000	2,620	6,089	<0,001	Yes
4,000 vs. 1,000	2,241	5,210	<0,001	Yes
4,000 vs. 5,000	1,387	3,223	0,021	Yes
2,000 vs. 3,000	1,335	3,103	0,026	Yes
4,000 vs. 2,000	1,285	2,986	0,030	Yes
5,000 vs. 3,000	1,233	2,866	0,034	Yes
2,000 vs. 1,000	0,957	2,224	0,124	No
5,000 vs. 1,000	0,855	1,987	0,155	No
1,000 vs. 3,000	0,378	0,879	0,622	No
2,000 vs. 5,000	0,102	0,237	0,814	No

Comparisons for factor: **month**

Comparison	Diff of Means	t	P	P<0,050
june vs. august	3,029	9,090	<0,001	Yes
june vs. july	1,854	5,562	<0,001	Yes
july vs. august	1,175	3,527	0,001	Yes

Comparisons for factor: **year**

Comparison	Diff of Means	t	P	P<0,050
2017,000 vs. 2018,000	0,887	2,663	0,034	Yes
2016,000 vs. 2018,000	0,727	2,182	0,070	No
2017,000 vs. 2016,000	0,160	0,481	0,634	No

Dependent Variable: **KAEM_TOT**

Normality Test (Shapiro-Wilk): Passed (P = 0,214)

Equal Variance Test (Brown-Forsythe): Passed (P = 1,000)

Source of Variation	DF	SS	MS	F	P
individual	4	20,275	5,069	18,324	<0,001
month	2	4,704	2,352	8,503	<0,001
year	2	1,107	0,553	2,000	0,150
Residual	36	9,958	0,277		
Total	44	36,045	0,819		

Comparisons for factor: **individual**

Comparison	Diff of Means	t	P	P<0,050
2,000 vs. 5,000	1,804	7,276	<0,001	Yes
1,000 vs. 5,000	1,508	6,080	<0,001	Yes
2,000 vs. 3,000	1,391	5,609	<0,001	Yes
4,000 vs. 5,000	1,103	4,450	<0,001	Yes
1,000 vs. 3,000	1,094	4,413	<0,001	Yes
2,000 vs. 4,000	0,701	2,826	0,038	Yes
4,000 vs. 3,000	0,690	2,782	0,038	Yes
3,000 vs. 5,000	0,413	1,668	0,281	No
1,000 vs. 4,000	0,404	1,630	0,281	No
2,000 vs. 1,000	0,297	1,196	0,281	No

Comparisons for factor: **month**

Comparison	Diff of Means	t	P	P<0,050
june vs. august	0,786	4,090	<0,001	Yes
june vs. july	0,480	2,499	0,034	Yes
july vs. august	0,306	1,592	0,120	No

Comparisons for factor: **year**

Comparison	Diff of Means	t	P	P<0,050
2017,000 vs. 2016,000	0,337	1,754	0,241	No
2017,000 vs. 2018,000	0,328	1,709	0,241	No
2018,000 vs. 2016,000	0,00857	0,0446	0,965	No

Dependent Variable: **QUER_TOT**
Normality Test (Shapiro-Wilk): Failed (P < 0,050)
Equal Variance Test (Brown-Forsythe): Passed (P = 1,000)

Source of Variation	DF	SS	MS	F	P
individual	4	1,908	0,477	1,822	0,146
month	2	6,102	3,051	11,652	<0,001
year	2	5,178	2,589	9,888	<0,001
Residual	36	9,426	0,262		
Total	44	22,615	0,514		

Comparisons for factor: **individual**

Comparison	Diff of Means	t	P	P<0,050
2,000 vs. 5,000	0,567	2,349	0,219	No
4,000 vs. 5,000	0,525	2,175	0,283	No
3,000 vs. 5,000	0,468	1,939	0,393	No
1,000 vs. 5,000	0,314	1,303	0,792	No
2,000 vs. 1,000	0,252	1,046	0,885	No
4,000 vs. 1,000	0,210	0,872	0,915	No
3,000 vs. 1,000	0,153	0,636	0,951	No
2,000 vs. 3,000	0,0990	0,410	0,968	No
4,000 vs. 3,000	0,0570	0,236	0,968	No
2,000 vs. 4,000	0,0420	0,174	0,968	No

Comparisons for factor: **month**

Comparison	Diff of Means	t	P	P<0,050
june vs. august	0,886	4,744	<0,001	Yes
june vs. july	0,588	3,148	0,007	Yes
july vs. august	0,298	1,596	0,119	No

Comparisons for factor: **year**

Comparison	Diff of Means	t	P	P<0,050
2016,000 vs. 2018,000	0,720	3,852	0,001	Yes
2017,000 vs. 2018,000	0,719	3,850	0,001	Yes
2016,000 vs. 2017,000	0,000255	0,00136	0,999	No

Dependent Variable: **MYRI_TOT**
Normality Test (Shapiro-Wilk): Passed (P = 0,192)
Equal Variance Test (Brown-Forsythe): Passed (P = 1,000)

Source of Variation	DF	SS	MS	F	P
individual	4	1,167	0,292	50,798	<0,001
month	2	0,115	0,0577	10,046	<0,001
year	2	0,00618	0,00309	0,538	0,588
Residual	36	0,207	0,00574		
Total	44	1,495	0,0340		

Comparisons for factor: **individual**

Comparison	Diff of Means	t	P	P<0,050
4,000 vs. 5,000	0,472	13,228	<0,001	Yes
4,000 vs. 1,000	0,371	10,393	<0,001	Yes
4,000 vs. 2,000	0,346	9,697	<0,001	Yes
4,000 vs. 3,000	0,240	6,715	<0,001	Yes
3,000 vs. 5,000	0,233	6,514	<0,001	Yes
3,000 vs. 1,000	0,131	3,679	0,004	Yes
2,000 vs. 5,000	0,126	3,532	0,005	Yes
3,000 vs. 2,000	0,107	2,982	0,015	Yes
1,000 vs. 5,000	0,101	2,835	0,015	Yes
2,000 vs. 1,000	0,0249	0,697	0,491	No

Comparisons for factor: **month**

Comparison	Diff of Means	t	P	P<0,050
june vs. august	0,124	4,473	<0,001	Yes
june vs. july	0,0687	2,484	0,035	Yes
july vs. august	0,0550	1,990	0,054	No

Comparisons for factor: **year**

Comparison	Diff of Means	t	P	P<0,050
2017,000 vs. 2016,000	0,0277	1,002	0,690	No
2017,000 vs. 2018,000	0,0203	0,735	0,716	No
2018,000 vs. 2016,000	0,00740	0,267	0,791	No

Table S15. The results of statistical analyses of *Quercus robur*. The differences in the mean values of quantitative results of each polyphenol group between individual trees, years and sampling times were analyzed using three-way analysis of variance (Three-way ANOVA). Pairwise multiple comparison procedures were performed with Holm-Sidak method, when significant differences were detected. The significance level was set to p=0.05.

Dependent Variable: **[GALL_TOT]**
Normality Test (Shapiro-Wilk): Failed (P < 0,050)
Equal Variance Test (Brown-Forsythe): Passed (P = 1,000)

Source of Variation	DF	SS	MS	F	P
Individual	4	12,664	3,166	5,564	0,001
Month	2	49,826	24,913	43,783	<0,001
Year	2	10,084	5,042	8,861	<0,001
Residual	36	20,484	0,569		
Total	44	93,057	2,115		

Comparisons for factor: **Individual**

Comparison	Diff of Means	t	P	P<0,050
5,000 vs. 2,000	1,484	4,172	0,002	Yes
3,000 vs. 2,000	1,285	3,615	0,008	Yes
4,000 vs. 2,000	1,136	3,193	0,023	Yes
5,000 vs. 1,000	0,815	2,292	0,180	No
1,000 vs. 2,000	0,669	1,881	0,345	No
3,000 vs. 1,000	0,617	1,734	0,381	No
4,000 vs. 1,000	0,467	1,312	0,586	No
5,000 vs. 4,000	0,348	0,979	0,705	No
5,000 vs. 3,000	0,198	0,558	0,824	No
3,000 vs. 4,000	0,150	0,421	0,824	No

Comparisons for factor: **Month**

Comparison	Diff of Means	t	P	P<0,050
June vs. August	2,373	8,616	<0,001	Yes
June vs. July	2,057	7,469	<0,001	Yes
July vs. August	0,316	1,147	0,259	No

Comparisons for factor: **Year**

Comparison	Diff of Means	t	P	P<0,050
2017,000 vs. 2018,000	1,071	3,889	0,001	Yes
2017,000 vs. 2016,000	0,920	3,340	0,004	Yes
2016,000 vs. 2018,000	0,151	0,549	0,586	No

Dependent Variable: **[HHDP_TOT]**
Normality Test (Shapiro-Wilk): Passed (P = 0,967)
Equal Variance Test (Brown-Forsythe): Passed (P = 1,000)

Source of Variation	DF	SS	MS	F	P
Individual	4	398,758	99,690	11,298	<0,001
Month	2	1158,095	579,047	65,627	<0,001
Year	2	428,732	214,366	24,295	<0,001
Residual	36	317,638	8,823		
Total	44	2303,224	52,346		

Comparisons for factor: **Individual**

Comparison	Diff of Means	t	P	P<0,050
4,000 vs. 1,000	8,255	5,895	<0,001	Yes
4,000 vs. 2,000	7,112	5,079	<0,001	Yes
5,000 vs. 1,000	5,476	3,911	0,003	Yes
4,000 vs. 3,000	5,011	3,579	0,007	Yes
5,000 vs. 2,000	4,333	3,095	0,023	Yes

3,000 vs. 1,000	3,243	2,316	0,125	No
4,000 vs. 5,000	2,779	1,984	0,202	No
5,000 vs. 3,000	2,233	1,594	0,318	No
3,000 vs. 2,000	2,100	1,500	0,318	No
2,000 vs. 1,000	1,143	0,816	0,420	No

Comparisons for factor: **Month**

**Comparison**	**Diff of Means**	**t**	**P**	**P<0,050**
June vs. August	11,597	10,692	<0,001	Yes
June vs. July	9,664	8,910	<0,001	Yes
July vs. August	1,932	1,782	0,083	No

Comparisons for factor: **Year**

**Comparison**	**Diff of Means**	**t**	**P**	**P<0,050**
2017,000 vs. 2018,000	7,458	6,876	<0,001	Yes
2017,000 vs. 2016,000	4,806	4,431	<0,001	Yes
2016,000 vs. 2018,000	2,652	2,445	0,020	Yes

Dependent Variable: **[PC_TOT]**
Normality Test (Shapiro-Wilk): Passed (P = 0,144)
Equal Variance Test (Brown-Forsythe): Passed (P = 1,000)

**Source of Variation**	**DF**	**SS**	**MS**	**F**	**P**
Individual	4	3663,435	915,859	18,498	<0,001
Month	2	3612,927	1806,464	36,485	<0,001
Year	2	100,373	50,187	1,014	0,373
Residual	36	1782,438	49,512		
Total	44	9159,173	208,163		

Comparisons for factor: **Individual**

**Comparison**	**Diff of Means**	**t**	**P**	**P<0,050**
1,000 vs. 4,000	23,387	7,051	<0,001	Yes
1,000 vs. 2,000	22,527	6,791	<0,001	Yes
3,000 vs. 4,000	16,658	5,022	<0,001	Yes
3,000 vs. 2,000	15,798	4,763	<0,001	Yes
1,000 vs. 5,000	12,696	3,828	0,003	Yes
5,000 vs. 4,000	10,691	3,223	0,013	Yes
5,000 vs. 2,000	9,831	2,964	0,021	Yes
1,000 vs. 3,000	6,729	2,029	0,142	No
3,000 vs. 5,000	5,967	1,799	0,154	No
2,000 vs. 4,000	0,860	0,259	0,797	No

Comparisons for factor: **Month**

**Comparison**	**Diff of Means**	**t**	**P**	**P<0,050**
August vs. June	19,429	7,562	<0,001	Yes
July vs. June	18,556	7,222	<0,001	Yes
August vs. July	0,873	0,340	0,736	No

Comparisons for factor: **Year**

**Comparison**	**Diff of Means**	**t**	**P**	**P<0,050**
2018,000 vs. 2016,000	3,653	1,422	0,415	No
2017,000 vs. 2016,000	1,997	0,777	0,689	No
2018,000 vs. 2017,000	1,656	0,644	0,689	No

Dependent Variable: **[PD_TOT]**
Normality Test (Shapiro-Wilk): Passed (P = 0,286)
Equal Variance Test (Brown-Forsythe): Passed (P = 1,000)

**Source of Variation**	**DF**	**SS**	**MS**	**F**	**P**
Individual	4	426,364	106,591	12,589	<0,001
Month	2	639,175	319,588	37,746	<0,001
Year	2	17,000	8,500	1,004	0,376
Residual	36	304,809	8,467		
Total	44	1387,348	31,531		

Comparisons for factor: **Individual**

**Comparison**	**Diff of Means**	**t**	**P**	**P<0,050**
1,000 vs. 3,000	8,746	6,376	<0,001	Yes
1,000 vs. 5,000	7,732	5,637	<0,001	Yes
1,000 vs. 4,000	6,870	5,009	<0,001	Yes
1,000 vs. 2,000	6,314	4,603	<0,001	Yes
2,000 vs. 3,000	2,432	1,773	0,412	No

4,000 vs. 3,000	1,875	1,367	0,629	No
2,000 vs. 5,000	1,418	1,034	0,771	No
5,000 vs. 3,000	1,013	0,739	0,847	No
4,000 vs. 5,000	0,862	0,628	0,847	No
2,000 vs. 4,000	0,557	0,406	0,847	No

Comparisons for factor: **Month**

**Comparison**	**Diff of Means**	**t**	**P**	**P<0,050**
August vs. June	8,104	7,627	<0,001	Yes
July vs. June	7,881	7,417	<0,001	Yes
August vs. July	0,223	0,210	0,835	No

Comparisons for factor: **Year**

**Comparison**	**Diff of Means**	**t**	**P**	**P<0,050**
2017,000 vs. 2018,000	1,363	1,283	0,503	No
2017,000 vs. 2016,000	1,235	1,162	0,503	No
2016,000 vs. 2018,000	0,128	0,120	0,905	No

Dependent Variable: **[QUIN_TOT]**
Normality Test (Shapiro-Wilk): Passed (P = 0,422)
Equal Variance Test (Brown-Forsythe): Passed (P = 1,000)

**Source of Variation**	**DF**	**SS**	**MS**	**F**	**P**
Individual	4	0,220	0,0549	2,841	0,038
Month	2	0,275	0,137	7,103	0,003
Year	2	0,512	0,256	13,246	<0,001
Residual	36	0,696	0,0193		
Total	44	1,702	0,0387		

Comparisons for factor: **Individual**

**Comparison**	**Diff of Means**	**t**	**P**	**P<0,050**
4,000 vs. 2,000	0,206	3,145	0,033	Yes
4,000 vs. 3,000	0,167	2,551	0,128	No
4,000 vs. 5,000	0,107	1,637	0,607	No
4,000 vs. 1,000	0,105	1,596	0,607	No
1,000 vs. 2,000	0,102	1,550	0,607	No
5,000 vs. 2,000	0,0988	1,508	0,607	No
1,000 vs. 3,000	0,0626	0,956	0,817	No
5,000 vs. 3,000	0,0599	0,914	0,817	No
3,000 vs. 2,000	0,0389	0,594	0,817	No
1,000 vs. 5,000	0,00272	0,0415	0,967	No

Comparisons for factor: **Month**

**Comparison**	**Diff of Means**	**t**	**P**	**P<0,050**
July vs. August	0,189	3,720	0,002	Yes
June vs. August	0,121	2,386	0,044	Yes
July vs. June	0,0677	1,334	0,191	No

Comparisons for factor: **Year**

**Comparison**	**Diff of Means**	**t**	**P**	**P<0,050**
2018,000 vs. 2016,000	0,252	4,954	<0,001	Yes
2018,000 vs. 2017,000	0,187	3,686	0,001	Yes
2017,000 vs. 2016,000	0,0644	1,268	0,213	No

Dependent Variable**: [KAEM_TOT]**
Normality Test (Shapiro-Wilk): Passed (P = 0,597)
Equal Variance Test (Brown-Forsythe): Passed (P = 1,000)

**Source of Variation**	**DF**	**SS**	**MS**	**F**	**P**
Individual	4	14,479	3,620	3,085	0,028
Month	2	221,660	110,830	94,450	<0,001
Year	2	10,041	5,021	4,279	0,022
Residual	36	42,244	1,173		
Total	44	288,424	6,555		

Comparisons for factor: **Individual**

**Comparison**	**Diff of Means**	**t**	**P**	**P<0,050**
2,000 vs. 4,000	1,264	2,475	0,168	No
2,000 vs. 5,000	1,227	2,402	0,178	No
1,000 vs. 4,000	1,199	2,349	0,180	No
1,000 vs. 5,000	1,162	2,276	0,186	No
2,000 vs. 3,000	1,049	2,054	0,252	No

1,000 vs. 3,000	0,984	1,928	0,273	No
3,000 vs. 4,000	0,215	0,421	0,989	No
3,000 vs. 5,000	0,178	0,348	0,989	No
2,000 vs. 1,000	0,0644	0,126	0,990	No
5,000 vs. 4,000	0,0372	0,0729	0,990	No

Comparisons for factor: **Month**

Comparison	Diff of Means	t	P	P<0,050
June vs. August	4,959	12,538	<0,001	Yes
June vs. July	4,408	11,145	<0,001	Yes
July vs. August	0,551	1,393	0,172	No

Comparisons for factor: **Year**

Comparison	Diff of Means	t	P	P<0,050
2016,000 vs. 2018,000	1,157	2,925	0,018	Yes
2017,000 vs. 2018,000	0,599	1,514	0,258	No
2016,000 vs. 2017,000	0,558	1,411	0,258	No

Dependent Variable: **[QUER_TOT]**
Normality Test (Shapiro-Wilk): Passed (P = 0,122)
Equal Variance Test (Brown-Forsythe): Passed (P = 1,000)

Source of Variation	DF	SS	MS	F	P
Individual	4	50,264	12,566	39,487	<0,001
Month	2	4,605	2,303	7,235	0,002
Year	2	13,015	6,508	20,450	<0,001
Residual	36	11,456	0,318		
Total	44	79,340	1,803		

Comparisons for factor: **Individual**

Comparison	Diff of Means	t	P	P<0,050
1,000 vs. 2,000	3,225	12,129	<0,001	Yes
1,000 vs. 4,000	2,304	8,664	<0,001	Yes
1,000 vs. 5,000	2,105	7,914	<0,001	Yes
1,000 vs. 3,000	1,779	6,690	<0,001	Yes
3,000 vs. 2,000	1,446	5,439	<0,001	Yes
5,000 vs. 2,000	1,121	4,215	<0,001	Yes
4,000 vs. 2,000	0,921	3,464	0,006	Yes
3,000 vs. 4,000	0,525	1,975	0,159	No
3,000 vs. 5,000	0,326	1,224	0,405	No
5,000 vs. 4,000	0,200	0,750	0,458	No

Comparisons for factor: **Month**

Comparison	Diff of Means	t	P	P<0,050
June vs. July	0,713	3,459	0,004	Yes
June vs. August	0,639	3,101	0,007	Yes
August vs. July	0,0738	0,358	0,722	No

Comparisons for factor: **Year**

Comparison	Diff of Means	t	P	P<0,050
2017,000 vs. 2018,000	1,315	6,386	<0,001	Yes
2016,000 vs. 2018,000	0,718	3,487	0,003	Yes
2017,000 vs. 2016,000	0,597	2,900	0,006	Yes

Dependent Variable: **[MYRI_TOT]**
Normality Test (Shapiro-Wilk): Failed (P < 0,050)
Equal Variance Test (Brown-Forsythe): Passed (P = 1,000)

Source of Variation	DF	SS	MS	F	P
Individual	4	0,942	0,235	29,261	<0,001
Month	2	0,0748	0,0374	4,648	0,016
Year	2	0,0189	0,00947	1,176	0,320
Residual	36	0,290	0,00805		
Total	44	1,325	0,0301		

Comparisons for factor: **Individual**

Comparison	Diff of Means	t	P	P<0,050
1,000 vs. 5,000	0,393	9,301	<0,001	Yes
1,000 vs. 3,000	0,368	8,701	<0,001	Yes
1,000 vs. 4,000	0,338	7,989	<0,001	Yes
1,000 vs. 2,000	0,330	7,802	<0,001	Yes
2,000 vs. 5,000	0,0634	1,500	0,602	No

4,000 vs. 5,000	0,0555	1,312	0,668	No
2,000 vs. 3,000	0,0380	0,899	0,847	No
4,000 vs. 3,000	0,0301	0,712	0,860	No
3,000 vs. 5,000	0,0254	0,600	0,860	No
2,000 vs. 4,000	0,00792	0,187	0,860	No

Comparisons for factor: **Month**

Comparison	Diff of Means	t	P	P<0,050
July vs. June	0,0908	2,770	0,026	Yes
August vs. June	0,0815	2,488	0,035	Yes
July vs. August	0,00926	0,283	0,779	No

Comparisons for factor: **Year**

Comparison	Diff of Means	t	P	P<0,050
2017,000 vs. 2018,000	0,0502	1,533	0,351	No
2017,000 vs. 2016,000	0,0263	0,803	0,672	No
2016,000 vs. 2018,000	0,0239	0,730	0,672	No

Table S16. The results of statistical analyses of *Acer platanoides*. The differences in the mean values of quantitative results of each polyphenol group between individual trees, years and sampling times were analyzed using three-way analysis of variance (Three-way ANOVA). Pairwise multiple comparison procedures were performed with Holm-Sidak method, when significant differences were detected. The significance level was set to p=0.05.

Dependent Variable: **[GALL_TOT]**
Normality Test (Shapiro-Wilk): Passed (P = 0,111)
Equal Variance Test (Brown-Forsythe): Passed (P = 1,000)

Source of Variation	DF	SS	MS	F	P
Individual	4	343,377	85,844	12,589	<0,001
Month	2	29,930	14,965	2,195	0,126
Year	2	193,761	96,880	14,208	<0,001
Residual	36	245,477	6,819		
Total	44	812,545	18,467		

Comparisons for factor: **Individual**

Comparison	Diff of Means	t	P	P<0,050
5,000 vs. 2,000	6,908	5,612	<0,001	Yes
1,000 vs. 2,000	6,758	5,490	<0,001	Yes
4,000 vs. 2,000	6,215	5,049	<0,001	Yes
5,000 vs. 3,000	4,424	3,594	0,007	Yes
1,000 vs. 3,000	4,275	3,473	0,008	Yes
4,000 vs. 3,000	3,732	3,032	0,022	Yes
3,000 vs. 2,000	2,484	2,018	0,189	No
5,000 vs. 4,000	0,692	0,562	0,924	No
1,000 vs. 4,000	0,543	0,441	0,924	No
5,000 vs. 1,000	0,149	0,121	0,924	No

Comparisons for factor: **Month**

Comparison	Diff of Means	t	P	P<0,050
June vs. August	1,990	2,087	0,126	No
July vs. August	1,149	1,205	0,416	No
June vs. July	0,840	0,881	0,416	No

Comparisons for factor: **Year**

Comparison	Diff of Means	t	P	P<0,050
2017,000 vs. 2018,000	4,726	4,956	<0,001	Yes
2017,000 vs. 2016,000	3,983	4,177	<0,001	Yes
2016,000 vs. 2018,000	0,743	0,779	0,441	No

Dependent Variable: **[HHDP_TOT]**
Normality Test (Shapiro-Wilk): Failed (P < 0,050)
Equal Variance Test (Brown-Forsythe): Passed (P = 1,000)

Source of Variation	DF	SS	MS	F	P
Individual	4	0,000	0,000	0,000	1,000
Month	2	0,000	0,000	0,000	1,000
Year	2	0,000	0,000	0,000	1,000
Residual	36	0,000	0,000		
Total	44	0,000	0,000		

Dependent Variable: **[PC_TOT]**
Normality Test (Shapiro-Wilk): Failed (P < 0,050)
Equal Variance Test (Brown-Forsythe): Passed (P = 1,000)

**Source of Variation**	**DF**	**SS**	**MS**	**F**	**P**
Individual	4	3180,910	795,227	9,506	<0,001
Month	2	3490,425	1745,213	20,863	<0,001
Year	2	70,492	35,246	0,421	0,659
Residual	36	3011,459	83,652		
Total	44	9753,287	221,666		

Comparisons for factor: **Individual**

**Comparison**	**Diff of Means**	**t**	**P**	**P<0,050**
5,000 vs. 1,000	22,682	5,261	<0,001	Yes
5,000 vs. 2,000	21,484	4,983	<0,001	Yes
5,000 vs. 3,000	17,115	3,970	0,003	Yes
4,000 vs. 1,000	12,760	2,960	0,037	Yes
4,000 vs. 2,000	11,563	2,682	0,064	No
5,000 vs. 4,000	9,922	2,301	0,129	No
4,000 vs. 3,000	7,193	1,668	0,355	No
3,000 vs. 1,000	5,567	1,291	0,497	No
3,000 vs. 2,000	4,369	1,013	0,534	No
2,000 vs. 1,000	1,197	0,278	0,783	No

Comparisons for factor: **Month**

**Comparison**	**Diff of Means**	**t**	**P**	**P<0,050**
August vs. June	20,007	5,991	<0,001	Yes
July vs. June	16,992	5,088	<0,001	Yes
August vs. July	3,014	0,903	0,373	No

Comparisons for factor: **Year**

**Comparison**	**Diff of Means**	**t**	**P**	**P<0,050**
2017,000 vs. 2016,000	3,054	0,915	0,746	No
2018,000 vs. 2016,000	1,757	0,526	0,842	No
2017,000 vs. 2018,000	1,298	0,389	0,842	No

Dependent Variable**: [PD_TOT]**
Normality Test (Shapiro-Wilk): Failed (P < 0,050)
Equal Variance Test (Brown-Forsythe): Passed (P = 1,000)

**Source of Variation**	**DF**	**SS**	**MS**	**F**	**P**
Individual	4	2,611	0,653	2,543	0,056
Month	2	0,634	0,317	1,235	0,303
Year	2	0,0904	0,0452	0,176	0,839
Residual	36	9,240	0,257		
Total	44	12,575	0,286		

Dependent Variable**: [QUIN_TOT]**
Normality Test (Shapiro-Wilk): Passed (P = 0,377)
Equal Variance Test (Brown-Forsythe): Passed (P = 1,000)

**Source of Variation**	**DF**	**SS**	**MS**	**F**	**P**
Individual	4	18,921	4,730	60,061	<0,001
Month	2	3,318	1,659	21,062	<0,001
Year	2	0,203	0,101	1,286	0,289
Residual	36	2,835	0,0788		
Total	44	25,276	0,574		

Comparisons for factor: **Individual**

**Comparison**	**Diff of Means**	**t**	**P**	**P<0,050**
5,000 vs. 1,000	1,616	12,212	<0,001	Yes
5,000 vs. 2,000	1,604	12,128	<0,001	Yes
4,000 vs. 1,000	1,236	9,346	<0,001	Yes
4,000 vs. 2,000	1,225	9,262	<0,001	Yes
5,000 vs. 3,000	1,026	7,756	<0,001	Yes
4,000 vs. 3,000	0,647	4,890	<0,001	Yes
3,000 vs. 1,000	0,589	4,456	<0,001	Yes
3,000 vs. 2,000	0,578	4,372	<0,001	Yes
5,000 vs. 4,000	0,379	2,866	0,014	Yes
2,000 vs. 1,000	0,0110	0,0834	0,934	No

Comparisons for factor: **Month**

Comparison	Diff of Means	t	P	P<0,050
June vs. August	0,661	6,448	<0,001	Yes
June vs. July	0,396	3,862	<0,001	Yes
July vs. August	0,265	2,586	0,014	Yes

Comparisons for factor: **Year**

Comparison	Diff of Means	t	P	P<0,050
2016,000 vs. 2018,000	0,153	1,497	0,371	No
2017,000 vs. 2018,000	0,128	1,248	0,392	No
2016,000 vs. 2017,000	0,0255	0,249	0,805	No

Dependent Variable: **[KAEM_TOT]**
Normality Test (Shapiro-Wilk): Passed (P = 0,659)
Equal Variance Test (Brown-Forsythe): Passed (P = 1,000)

Source of Variation	DF	SS	MS	F	P
Individual	4	2,000	0,500	11,318	<0,001
Month	2	0,557	0,278	6,299	0,005
Year	2	0,274	0,137	3,107	0,057
Residual	36	1,590	0,0442		
Total	44	4,422	0,100		

Comparisons for factor: **Individual**

Comparison	Diff of Means	t	P	P<0,050
3,000 vs. 5,000	0,630	6,355	<0,001	Yes
3,000 vs. 4,000	0,495	4,992	<0,001	Yes
3,000 vs. 1,000	0,422	4,261	0,001	Yes
3,000 vs. 2,000	0,417	4,208	0,001	Yes
2,000 vs. 5,000	0,213	2,147	0,210	No
1,000 vs. 5,000	0,208	2,095	0,210	No
4,000 vs. 5,000	0,135	1,364	0,550	No
2,000 vs. 4,000	0,0777	0,784	0,823	No
1,000 vs. 4,000	0,0724	0,731	0,823	No
2,000 vs. 1,000	0,00523	0,0528	0,958	No

Comparisons for factor: **Month**

Comparison	Diff of Means	t	P	P<0,050
June vs. July	0,259	3,375	0,005	Yes
August vs. July	0,203	2,640	0,024	Yes
June vs. August	0,0564	0,735	0,467	No

Comparisons for factor: **Year**

Comparison	Diff of Means	t	P	P<0,050
2016,000 vs. 2018,000	0,167	2,180	0,104	No
2016,000 vs. 2017,000	0,164	2,137	0,104	No
2017,000 vs. 2018,000	0,00332	0,0432	0,966	No

Dependent Variable**: [QUER_TOT]**
Normality Test (Shapiro-Wilk): Passed (P = 0,165)
Equal Variance Test (Brown-Forsythe): Passed (P = 1,000)

Source of Variation	DF	SS	MS	F	P
Individual	4	5,168	1,292	2,232	0,085
Month	2	4,910	2,455	4,241	0,022
Year	2	4,472	2,236	3,863	0,030
Residual	36	20,839	0,579		
Total	44	35,389	0,804		

Comparisons for factor: **Individual**

Comparison	Diff of Means	t	P	P<0,050
1,000 vs. 5,000	0,798	2,225	0,281	No
3,000 vs. 5,000	0,797	2,222	0,281	No
4,000 vs. 5,000	0,715	1,994	0,358	No
1,000 vs. 2,000	0,616	1,717	0,501	No
3,000 vs. 2,000	0,615	1,715	0,501	No
4,000 vs. 2,000	0,533	1,486	0,546	No
2,000 vs. 5,000	0,182	0,508	0,978	No
1,000 vs. 4,000	0,0829	0,231	0,994	No
3,000 vs. 4,000	0,0820	0,229	0,994	No
1,000 vs. 3,000	0,000909	0,00253	0,998	No

Comparisons for factor: **Month**

Comparison	Diff of Means	t	P	P<0,050
August vs. June	0,809	2,912	0,018	Yes
July vs. June	0,417	1,500	0,264	No
August vs. July	0,392	1,412	0,264	No

Comparisons for factor: **Year**

Comparison	Diff of Means	t	P	P<0,050
2018,000 vs. 2016,000	0,744	2,679	0,033	Yes
2017,000 vs. 2016,000	0,550	1,981	0,107	No
2018,000 vs. 2017,000	0,194	0,698	0,490	No

Dependent Variable: **[MYRI_TOT]**
Normality Test (Shapiro-Wilk): Failed (P < 0,050)
Equal Variance Test (Brown-Forsythe): Passed (P = 1,000)

Source of Variation	DF	SS	MS	F	P
Individual	4	0,000	0,000	0,000	1,000
Month	2	0,000	0,000	0,000	1,000
Year	2	0,000	0,000	0,000	1,000
Residual	36	0,000	0,000		
Total	44	0,000	0,000		

Table S17. The results of statistical analyses of *Alnus glutinosa*. The differences in the mean values of quantitative results of each polyphenol group between individual trees, years and sampling times were analyzed using three-way analysis of variance (Three-way ANOVA). Pairwise multiple comparison procedures were performed with Holm-Sidak method, when significant differences were detected. The significance level was set to p=0.05.

Dependent Variable: **[GALL_TOT]**
Normality Test (Shapiro-Wilk): Failed (P < 0,050)
Equal Variance Test (Brown-Forsythe): Passed (P = 1,000)

Source of Variation	DF	SS	MS	F	P
Individual	4	173,913	43,478	38,903	<0,001
Month	2	1,695	0,847	0,758	0,476
Year	2	17,894	8,947	8,006	0,001
Residual	36	40,233	1,118		
Total	44	233,735	5,312		

Comparisons for factor: **Individual**

Comparison	Diff of Means	t	P	P<0,050
4,000 vs. 5,000	5,215	10,465	<0,001	Yes
4,000 vs. 2,000	5,068	10,170	<0,001	Yes
4,000 vs. 3,000	3,886	7,799	<0,001	Yes
1,000 vs. 5,000	3,056	6,132	<0,001	Yes
1,000 vs. 2,000	2,909	5,837	<0,001	Yes
4,000 vs. 1,000	2,159	4,333	<0,001	Yes
1,000 vs. 3,000	1,727	3,466	0,006	Yes
3,000 vs. 5,000	1,329	2,666	0,034	Yes
3,000 vs. 2,000	1,182	2,372	0,046	Yes
2,000 vs. 5,000	0,147	0,294	0,770	No

Comparisons for factor: **Month**

Comparison	Diff of Means	t	P	P<0,050
June vs. August	0,419	1,085	0,635	No
June vs. July	0,404	1,047	0,635	No
July vs. August	0,0149	0,0386	0,969	No

Comparisons for factor: **Year**

Comparison	Diff of Means	t	P	P<0,050
2017,000 vs. 2016,000	1,536	3,979	<0,001	Yes
2017,000 vs. 2018,000	0,908	2,352	0,048	Yes
2018,000 vs. 2016,000	0,628	1,627	0,112	No

Dependent Variable: **[HHDP_TOT]**
Normality Test (Shapiro-Wilk): Passed (P = 0,079)
Equal Variance Test (Brown-Forsythe): Passed (P = 1,000)

Source of Variation	DF	SS	MS	F	P
Individual	4	14111,613	3527,903	31,338	<0,001
Month	2	4,029	2,015	0,0179	0,982
Year	2	2387,583	1193,791	10,604	<0,001
Residual	36	4052,692	112,575		
Total	44	20555,917	467,180		

Comparisons for factor: **Individual**

Comparison	Diff of Means	t	P	P<0,050
4,000 vs. 5,000	47,995	9,596	<0,001	Yes
4,000 vs. 2,000	47,619	9,521	<0,001	Yes
4,000 vs. 3,000	39,256	7,849	<0,001	Yes
4,000 vs. 1,000	31,681	6,334	<0,001	Yes
1,000 vs. 5,000	16,315	3,262	0,014	Yes
1,000 vs. 2,000	15,938	3,187	0,015	Yes
3,000 vs. 5,000	8,739	1,747	0,312	No
3,000 vs. 2,000	8,362	1,672	0,312	No
1,000 vs. 3,000	7,575	1,515	0,312	No
2,000 vs. 5,000	0,377	0,0753	0,940	No

Comparisons for factor: **Month**

Comparison	Diff of Means	t	P	P<0,050
June vs. August	0,705	0,182	0,997	No
July vs. August	0,527	0,136	0,997	No
June vs. July	0,177	0,0457	0,997	No

Comparisons for factor: **Year**

Comparison	Diff of Means	t	P	P<0,050
2017,000 vs. 2016,000	17,763	4,585	<0,001	Yes
2018,000 vs. 2016,000	10,333	2,667	0,023	Yes
2017,000 vs. 2018,000	7,430	1,918	0,063	No

Dependent Variable: **[PC_TOT]**
Normality Test (Shapiro-Wilk): Passed (P = 0,732)
Equal Variance Test (Brown-Forsythe): Passed (P = 1,000)

Source of Variation	DF	SS	MS	F	P
Individual	4	46,967	11,742	41,606	<0,001
Month	2	1,920	0,960	3,402	0,044
Year	2	6,896	3,448	12,217	<0,001
Residual	36	10,160	0,282		
Total	44	65,943	1,499		

Comparisons for factor: **Individual**

Comparison	Diff of Means	t	P	P<0,050
4,000 vs. 5,000	2,287	9,133	<0,001	Yes
1,000 vs. 5,000	2,177	8,695	<0,001	Yes
3,000 vs. 5,000	2,086	8,329	<0,001	Yes
4,000 vs. 2,000	2,074	8,281	<0,001	Yes
1,000 vs. 2,000	1,964	7,843	<0,001	Yes
3,000 vs. 2,000	1,872	7,477	<0,001	Yes
2,000 vs. 5,000	0,213	0,852	0,870	No
4,000 vs. 3,000	0,201	0,804	0,870	No
4,000 vs. 1,000	0,110	0,438	0,887	No
1,000 vs. 3,000	0,0918	0,366	0,887	No

Comparisons for factor: **Month**

Comparison	Diff of Means	t	P	P<0,050
August vs. June	0,473	2,438	0,058	No
July vs. June	0,392	2,023	0,099	No
August vs. July	0,0804	0,414	0,681	No

Comparisons for factor: **Year**

Comparison	Diff of Means	t	P	P<0,050
2018,000 vs. 2016,000	0,941	4,851	<0,001	Yes
2017,000 vs. 2016,000	0,630	3,249	0,005	Yes
2018,000 vs. 2017,000	0,311	1,602	0,118	No

Dependent Variable: **[PD_TOT]**
Normality Test (Shapiro-Wilk): Failed (P < 0,050)
Equal Variance Test (Brown-Forsythe): Passed (P = 1,000)

Source of Variation	DF	SS	MS	F	P
Individual	4	0,000	0,000	0,000	1,000
Month	2	0,000	0,000	0,000	1,000
Year	2	0,000	0,000	0,000	1,000
Residual	36	0,000	0,000		
Total	44	0,000	0,000		

Dependent Variable: **[QUIN_TOT]**
Normality Test (Shapiro-Wilk): Passed (P = 0,491)
Equal Variance Test (Brown-Forsythe): Passed (P = 1,000)

Source of Variation	DF	SS	MS	F	P
Individual	4	67,360	16,840	58,108	<0,001
Month	2	0,379	0,190	0,655	0,526
Year	2	1,446	0,723	2,495	0,097
Residual	36	10,433	0,290		
Total	44	79,619	1,810		

Comparisons for factor: **Individual**

Comparison	Diff of Means	t	P	P<0,050
4,000 vs. 3,000	2,993	11,793	<0,001	Yes
4,000 vs. 5,000	2,730	10,758	<0,001	Yes
2,000 vs. 3,000	2,548	10,039	<0,001	Yes
2,000 vs. 5,000	2,285	9,004	<0,001	Yes
1,000 vs. 3,000	2,147	8,460	<0,001	Yes
1,000 vs. 5,000	1,884	7,424	<0,001	Yes
4,000 vs. 1,000	0,846	3,333	0,008	Yes
4,000 vs. 2,000	0,445	1,754	0,241	No
2,000 vs. 1,000	0,401	1,579	0,241	No
5,000 vs. 3,000	0,263	1,035	0,307	No

Comparisons for factor: **Month**

Comparison	Diff of Means	t	P	P<0,050
June vs. August	0,223	1,132	0,603	No
June vs. July	0,139	0,709	0,733	No
July vs. August	0,0833	0,424	0,733	No

Comparisons for factor: **Year**

Comparison	Diff of Means	t	P	P<0,050
2017,000 vs. 2018,000	0,434	2,205	0,098	No
2016,000 vs. 2018,000	0,277	1,409	0,307	No
2017,000 vs. 2016,000	0,156	0,796	0,431	No

Dependent Variable: **[KAEM_TOT]**
Normality Test (Shapiro-Wilk): Passed (P = 0,249)
Equal Variance Test (Brown-Forsythe): Passed (P = 1,000)

Source of Variation	DF	SS	MS	F	P
Individual	4	0,242	0,0604	20,180	<0,001
Month	20,000211		0,000106	0,0353	0,965
Year	20,0609		0,0304	10,163	<0,001
Residual	36	0,108	0,00299		
Total	44	0,411	0,00933		

Comparisons for factor: **Individual**

Comparison	Diff of Means	t	P	P<0,050
4,000 vs. 5,000	0,195	7,555	<0,001	Yes
1,000 vs. 5,000	0,182	7,061	<0,001	Yes
4,000 vs. 2,000	0,125	4,854	<0,001	Yes
4,000 vs. 3,000	0,113	4,398	<0,001	Yes
1,000 vs. 2,000	0,112	4,360	<0,001	Yes
1,000 vs. 3,000	0,101	3,904	0,002	Yes
3,000 vs. 5,000	0,0814	3,157	0,013	Yes
2,000 vs. 5,000	0,0697	2,702	0,031	Yes
4,000 vs. 1,000	0,0127	0,494	0,859	No
3,000 vs. 2,000	0,0118	0,456	0,859	No

Comparisons for factor: **Month**

Comparison	Diff of Means	t	P	P<0,050
August vs. June	0,00527	0,264	0,991	No
August vs. July	0,00319	0,159	0,991	No
July vs. June	0,00208	0,104	0,991	No

Comparisons for factor: **Year**

Comparison	Diff of Means	t	P	P<0,050
2018,000 vs. 2016,000	0,0859	4,301	<0,001	Yes
2017,000 vs. 2016,000	0,0663	3,320	0,004	Yes
2018,000 vs. 2017,000	0,0196	0,981	0,333	No

Dependent Variable**: [QUER_TOT]**
Normality Test (Shapiro-Wilk): Passed (P = 0,296)
Equal Variance Test (Brown-Forsythe): Passed (P = 1,000)

Source of Variation	DF	SS	MS	F	P
Individual	4	2,068	0,517	4,274	0,006
Month	2	0,0795	0,0398	0,329	0,722
Year	2	0,534	0,267	2,210	0,124
Residual	36	4,354	0,121		
Total	44	7,036	0,160		

Comparisons for factor: **Individual**

Comparison	Diff of Means	t	P	P<0,050
4,000 vs. 5,000	0,569	3,471	0,014	Yes
3,000 vs. 5,000	0,549	3,347	0,017	Yes
4,000 vs. 2,000	0,362	2,211	0,238	No
1,000 vs. 5,000	0,361	2,200	0,238	No
3,000 vs. 2,000	0,342	2,087	0,238	No
4,000 vs. 1,000	0,208	1,271	0,696	No
2,000 vs. 5,000	0,207	1,260	0,696	No
3,000 vs. 1,000	0,188	1,147	0,696	No
1,000 vs. 2,000	0,154	0,940	0,696	No
4,000 vs. 3,000	0,0204	0,124	0,902	No

Comparisons for factor: **Month**

Comparison	Diff of Means	t	P	P<0,050
June vs. July	0,102	0,801	0,813	No
June vs. August	0,0647	0,509	0,851	No
August vs. July	0,0371	0,292	0,851	No

Comparisons for factor: **Year**

Comparison	Diff of Means	t	P	P<0,050
2017,000 vs. 2018,000	0,267	2,100	0,123	No
2017,000 vs. 2016,000	0,144	1,132	0,460	No
2016,000 vs. 2018,000	0,123	0,968	0,460	No

Dependent Variable: **[MYRI_TOT]**
Normality Test (Shapiro-Wilk): Failed (P < 0,050)
Equal Variance Test (Brown-Forsythe): Passed (P = 1,000)

Source of Variation	DF	SS	MS	F	P
Individual	4	0,000	0,000	0,000	1,000
Month	2	0,000	0,000	0,000	1,000
Year	2	0,000	0,000	0,000	1,000
Residual	36	0,000	0,000		
Total	44	0,000	0,000		

The results of statistical analyses of *Alnus incana*. Due to the missing individuals after July 2017, the data was not balanced and statistical tests were not performed.

Table S18. The results of statistical analyses of *Salix phylicifolia*. The differences in the mean values of quantitative results of each polyphenol group between individual trees, years and sampling times were analyzed using three-way analysis of variance (Three-way ANOVA). Pairwise multiple comparison procedures were performed with Holm-Sidak method, when significant differences were detected. The significance level was set to p=0.05.

Dependent Variable: **[GALL_TOT]**
Normality Test (Shapiro-Wilk): Failed (P < 0,050)
Equal Variance Test (Brown-Forsythe): Passed (P = 1,000)

Source of Variation	DF	SS	MS	F	P
Individual	4	0,000	0,000	0,000	1,000
Month	2	0,000	0,000	0,000	1,000
Year	2	0,000	0,000	0,000	1,000
Residual	36	0,000	0,000		
Total	44	0,000	0,000		

Dependent Variable: **[HHDP_TOT]**
Normality Test (Shapiro-Wilk): Failed (P < 0,050)
Equal Variance Test (Brown-Forsythe): Passed (P = 1,000)

Source of Variation	DF	SS	MS	F	P
Individual	4	0,000	0,000	0,000	1,000
Month	2	0,000	0,000	0,000	1,000
Year	2	0,000	0,000	0,000	1,000
Residual	36	0,000	0,000		
Total	44	0,000	0,000		

Dependent Variable: **[PC_TOT]**
Normality Test (Shapiro-Wilk): Passed (P = 0,615)
Equal Variance Test (Brown-Forsythe): Passed (P = 1,000)

Source of Variation	DF	SS	MS	F	P
Individual	4	34,503	8,626	17,710	<0,001
Month	2	18,152	9,076	18,635	<0,001
Year	2	4,526	2,263	4,646	0,016
Residual	36	17,534	0,487		
Total	44	74,714	1,698		

Comparisons for factor: **Individual**

Comparison	Diff of Means	t	P	P<0,050
3,000 vs. 1,000	2,131	6,478	<0,001	Yes
5,000 vs. 1,000	2,046	6,219	<0,001	Yes
4,000 vs. 1,000	2,006	6,098	<0,001	Yes
3,000 vs. 2,000	1,479	4,496	<0,001	Yes
5,000 vs. 2,000	1,394	4,238	<0,001	Yes
4,000 vs. 2,000	1,354	4,117	0,001	Yes
2,000 vs. 1,000	0,652	1,982	0,203	No
3,000 vs. 4,000	0,125	0,380	0,975	No
3,000 vs. 5,000	0,0851	0,259	0,975	No
5,000 vs. 4,000	0,0398	0,121	0,975	No

Comparisons for factor: **Month**

Comparison	Diff of Means	t	P	P<0,050
July vs. June	1,398	5,488	<0,001	Yes
August vs. June	1,290	5,060	<0,001	Yes
July vs. August	0,109	0,428	0,672	No

Comparisons for factor: **Year**

Comparison	Diff of Means	t	P	P<0,050
2016,000 vs. 2017,000	0,705	2,765	0,027	Yes
2016,000 vs. 2018,000	0,636	2,494	0,034	Yes
2018,000 vs. 2017,000	0,0689	0,271	0,788	No

Dependent Variable: **[PD_TOT]**
Normality Test (Shapiro-Wilk): Passed (P = 0,487)
Equal Variance Test (Brown-Forsythe): Passed (P = 1,000)

Source of Variation	DF	SS	MS	F	P
Individual	4	205,931	51,483	5,233	0,002
Month	2	1031,901	515,950	52,440	<0,001
Year	2	62,427	31,213	3,172	0,054
Residual	36	354,201	9,839		
Total	44	1654,459	37,601		

Comparisons for factor: **Individual**

Comparison	Diff of Means	t	P	P<0,050
2,000 vs. 1,000	5,943	4,019	0,003	Yes
2,000 vs. 3,000	5,013	3,390	0,015	Yes
5,000 vs. 1,000	4,051	2,740	0,074	No
2,000 vs. 4,000	3,597	2,433	0,132	No
5,000 vs. 3,000	3,121	2,111	0,226	No

4,000 vs. 1,000	2,346	1,586	0,476	No
2,000 vs. 5,000	1,892	1,279	0,608	No
5,000 vs. 4,000	1,706	1,153	0,608	No
4,000 vs. 3,000	1,415	0,957	0,608	No
3,000 vs. 1,000	0,931	0,629	0,608	No

Comparisons for factor: **Month**

**Comparison**	**Diff of Means**	**t**	**P**	**P<0,050**
August vs. June	11,611	10,138	<0,001	Yes
July vs. June	7,245	6,326	<0,001	Yes
August vs. July	4,366	3,812	<0,001	Yes

Comparisons for factor: **Year**

**Comparison**	**Diff of Means**	**t**	**P**	**P<0,050**
2016,000 vs. 2018,000	2,780	2,427	0,060	No
2017,000 vs. 2018,000	2,059	1,798	0,155	No
2016,000 vs. 2017,000	0,720	0,629	0,533	No

Dependent Variable**: [QUIN_TOT]**
Normality Test (Shapiro-Wilk): Failed (P < 0,050)
Equal Variance Test (Brown-Forsythe): Passed (P = 1,000)

**Source of Variation**	**DF**	**SS**	**MS**	**F**	**P**
Individual	4	4,409	1,102	4,258	0,006
Month	2	0,648	0,324	1,251	0,298
Year	2	1,263	0,631	2,439	0,102
Residual	36	9,319	0,259		
Total	44	15,638	0,355		

Comparisons for factor: **Individual**

**Comparison**	**Diff of Means**	**t**	**P**	**P<0,050**
3,000 vs. 2,000	0,924	3,854	0,005	Yes
3,000 vs. 5,000	0,706	2,943	0,050	Yes
1,000 vs. 2,000	0,556	2,319	0,191	No
3,000 vs. 4,000	0,499	2,079	0,274	No
4,000 vs. 2,000	0,426	1,775	0,411	No
3,000 vs. 1,000	0,368	1,535	0,511	No
1,000 vs. 5,000	0,338	1,408	0,520	No
5,000 vs. 2,000	0,219	0,911	0,748	No
4,000 vs. 5,000	0,207	0,864	0,748	No
1,000 vs. 4,000	0,130	0,544	0,748	No

Comparisons for factor: **Month**

**Comparison**	**Diff of Means**	**t**	**P**	**P<0,050**
August vs. June	0,288	1,549	0,342	No
July vs. June	0,196	1,054	0,508	No
August vs. July	0,0919	0,495	0,624	No

Comparisons for factor: **Year**

**Comparison**	**Diff of Means**	**t**	**P**	**P<0,050**
2016,000 vs. 2017,000	0,356	1,919	0,177	No
2016,000 vs. 2018,000	0,354	1,907	0,177	No
2018,000 vs. 2017,000	0,00221	0,0119	0,991	No

Dependent Variable: **[KAEM_TOT]**
Normality Test (Shapiro-Wilk): Passed (P = 0,475)
Equal Variance Test (Brown-Forsythe): Passed (P = 1,000)

**Source of Variation**	**DF**	**SS**	**MS**	**F**	**P**
Individual	4	0,230	0,0576	18,115	<0,001
Month	2	0,00219	0,00109	0,344	0,711
Year	2	0,173	0,0867	27,252	<0,001
Residual	36	0,114	0,00318		
Total	44	0,520	0,0118		

Comparisons for factor: **Individual**

**Comparison**	**Diff of Means**	**t**	**P**	**P<0,050**
3,000 vs. 5,000	0,201	7,556	<0,001	Yes
4,000 vs. 5,000	0,175	6,570	<0,001	Yes
1,000 vs. 5,000	0,158	5,934	<0,001	Yes
3,000 vs. 2,000	0,102	3,846	0,003	Yes
2,000 vs. 5,000	0,0986	3,710	0,004	Yes

4,000 vs. 2,000	0,0760	2,860	0,035	Yes
1,000 vs. 2,000	0,0591	2,224	0,124	No
3,000 vs. 1,000	0,0431	1,621	0,304	No
3,000 vs. 4,000	0,0262	0,985	0,552	No
4,000 vs. 1,000	0,0169	0,636	0,552	No

Comparisons for factor: **Month**

Comparison	Diff of Means	t	P	P<0,050
August vs. July	0,0164	0,796	0,816	No
August vs. June	0,0123	0,598	0,816	No
June vs. July	0,00407	0,198	0,844	No

Comparisons for factor: **Year**

Comparison	Diff of Means	t	P	P<0,050
2016,000 vs. 2018,000	0,134	6,486	<0,001	Yes
2016,000 vs. 2017,000	0,130	6,297	<0,001	Yes
2017,000 vs. 2018,000	0,00389	0,189	0,851	No

Dependent Variable**: [QUER_TOT]**
Normality Test (Shapiro-Wilk): Failed (P < 0,050)
Equal Variance Test (Brown-Forsythe): Passed (P = 1,000)

Source of Variation	DF	SS	MS	F	P
Individual	4	19,395	4,849	44,458	<0,001
Month	2	0,704	0,352	3,230	0,051
Year	2	0,870	0,435	3,988	0,027
Residual	36	3,926	0,109		
Total	44	24,896	0,566		

Comparisons for factor: **Individual**

Comparison	Diff of Means	t	P	P<0,050
3,000 vs. 5,000	1,886	12,112	<0,001	Yes
3,000 vs. 2,000	1,624	10,430	<0,001	Yes
3,000 vs. 1,000	1,332	8,558	<0,001	Yes
3,000 vs. 4,000	0,965	6,197	<0,001	Yes
4,000 vs. 5,000	0,921	5,915	<0,001	Yes
4,000 vs. 2,000	0,659	4,233	<0,001	Yes
1,000 vs. 5,000	0,553	3,554	0,004	Yes
4,000 vs. 1,000	0,368	2,361	0,070	No
1,000 vs. 2,000	0,291	1,872	0,134	No
2,000 vs. 5,000	0,262	1,682	0,134	No

Comparisons for factor: **Month**

Comparison	Diff of Means	t	P	P<0,050
June vs. August	0,305	2,526	0,048	Yes
June vs. July	0,182	1,509	0,260	No
July vs. August	0,123	1,016	0,316	No

Comparisons for factor: **Year**

Comparison	Diff of Means	t	P	P<0,050
2016,000 vs. 2018,000	0,311	2,579	0,042	Yes
2017,000 vs. 2018,000	0,276	2,287	0,056	No
2016,000 vs. 2017,000	0,0352	0,292	0,772	No

Dependent Variable: **[MYRI_TOT]**
Normality Test (Shapiro-Wilk): Passed (P = 0,943)
Equal Variance Test (Brown-Forsythe): Passed (P = 1,000)

Source of Variation	DF	SS	MS	F	P
Individual	4	19,425	4,856	32,857	<0,001
Month	2	5,595	2,797	18,927	<0,001
Year	2	6,248	3,124	21,137	<0,001
Residual	36	5,321	0,148		
Total	44	36,589	0,832		

Comparisons for factor: **Individual**

Comparison	Diff of Means	t	P	P<0,050
3,000 vs. 4,000	1,771	9,772	<0,001	Yes
1,000 vs. 4,000	1,405	7,754	<0,001	Yes
3,000 vs. 5,000	1,401	7,730	<0,001	Yes
2,000 vs. 4,000	1,147	6,329	<0,001	Yes
1,000 vs. 5,000	1,035	5,712	<0,001	Yes

2,000 vs. 5,000	0,777	4,287	<0,001	Yes
3,000 vs. 2,000	0,624	3,444	0,006	Yes
5,000 vs. 4,000	0,370	2,042	0,139	No
3,000 vs. 1,000	0,366	2,019	0,139	No
1,000 vs. 2,000	0,258	1,425	0,163	No

Comparisons for factor: **Month**

Comparison	Diff of Means	t	P	P<0,050
June vs. August	0,860	6,123	<0,001	Yes
July vs. August	0,503	3,582	0,002	Yes
June vs. July	0,357	2,541	0,016	Yes

Comparisons for factor: **Year**

Comparison	Diff of Means	t	P	P<0,050
2017,000 vs. 2018,000	0,896	6,380	<0,001	Yes
2016,000 vs. 2018,000	0,600	4,275	<0,001	Yes
2017,000 vs. 2016,000	0,296	2,106	0,042	Yes

Table S19. The results of statistical analyses of *Sorbus aucuparia*. The differences in the mean values of quantitative results of each polyphenol group between individual trees, years and sampling times were analyzed using three-way analysis of variance (Three-way ANOVA). Pairwise multiple comparison procedures were performed with Holm-Sidak method, when significant differences were detected. The significance level was set to p=0.05.

Dependent Variable: **[GALL_TOT]**
Normality Test (Shapiro-Wilk): Failed (P < 0,050)
Equal Variance Test (Brown-Forsythe): Passed (P = 1,000)

Source of Variation	DF	SS	MS	F	P
Individual	4	0,000	0,000	0,000	1,000
Month	2	0,000	0,000	0,000	1,000
Year	2	0,000	0,000	0,000	1,000
Residual	36	0,000	0,000		
Total	44	0,000	0,000		

Dependent Variable: **[HHDP_TOT]**
Normality Test (Shapiro-Wilk): Failed (P < 0,050)
Equal Variance Test (Brown-Forsythe): Passed (P = 1,000)

Source of Variation	DF	SS	MS	F	P
Individual	4	0,000	0,000	0,000	1,000
Month	2	0,000	0,000	0,000	1,000
Year	2	0,000	0,000	0,000	1,000
Residual	36	0,000	0,000		
Total	44	0,000	0,000		

Dependent Variable**: [PC_TOT]**
Normality Test (Shapiro-Wilk): Passed (P = 0,802)
Equal Variance Test (Brown-Forsythe): Passed (P = 1,000)

Source of Variation	DF	SS	MS	F	P
Individual	4	1438,712	359,678	24,883	<0,001
Month	2	44,382	22,191	1,535	0,229
Year	2	277,053	138,527	9,583	<0,001
Residual	36	520,372	14,455		
Total	44	2280,520	51,830		

Comparisons for factor: **Individual**

Comparison	Diff of Means	t	P	P<0,050
5,000 vs. 1,000	14,780	8,247	<0,001	Yes
2,000 vs. 1,000	13,575	7,574	<0,001	Yes
5,000 vs. 4,000	11,031	6,155	<0,001	Yes
2,000 vs. 4,000	9,826	5,483	<0,001	Yes
3,000 vs. 1,000	8,983	5,012	<0,001	Yes
5,000 vs. 3,000	5,798	3,235	0,013	Yes
3,000 vs. 4,000	5,234	2,920	0,024	Yes
2,000 vs. 3,000	4,593	2,563	0,044	Yes
4,000 vs. 1,000	3,749	2,092	0,085	No
5,000 vs. 2,000	1,205	0,672	0,506	No

Comparisons for factor: **Month**

Comparison	Diff of Means	t	P	P<0,050
August vs. July	2,424	1,746	0,245	No
August vs. June	1,388	1,000	0,543	No
June vs. July	1,036	0,746	0,543	No

Comparisons for factor: **Year**

Comparison	Diff of Means	t	P	P<0,050
2017,000 vs. 2018,000	6,017	4,334	<0,001	Yes
2017,000 vs. 2016,000	3,753	2,703	0,021	Yes
2016,000 vs. 2018,000	2,264	1,631	0,112	No

Dependent Variable: **[PD_TOT]**
Normality Test (Shapiro-Wilk): Failed (P < 0,050)
Equal Variance Test (Brown-Forsythe): Passed (P = 1,000)

Source of Variation	DF	SS	MS	F	P
Individual	4	0,000	0,000	0,000	1,000
Month	2	0,000	0,000	0,000	1,000
Year	2	0,000	0,000	0,000	1,000
Residual	36	0,000	0,000		
Total	44	0,000	0,000		

Dependent Variable: **[QUIN_TOT]**
Normality Test (Shapiro-Wilk): Passed (P = 0,396)
Equal Variance Test (Brown-Forsythe): Passed (P = 1,000)

Source of Variation	DF	SS	MS	F	P
Individual	4	36,268	9,067	2,712	0,045
Month	2	7,726	3,863	1,155	0,326
Year	2	61,336	30,668	9,172	<0,001
Residual	36	120,373	3,344		
Total	44	225,703	5,130		

Comparisons for factor: **Individual**

Comparison	Diff of Means	t	P	P<0,050
5,000 vs. 1,000	2,574	2,986	0,049	Yes
5,000 vs. 2,000	2,229	2,586	0,118	No
5,000 vs. 4,000	1,363	1,581	0,649	No
3,000 vs. 1,000	1,355	1,572	0,649	No
5,000 vs. 3,000	1,219	1,414	0,663	No
4,000 vs. 1,000	1,211	1,405	0,663	No
3,000 vs. 2,000	1,010	1,172	0,682	No
4,000 vs. 2,000	0,866	1,005	0,688	No
2,000 vs. 1,000	0,345	0,400	0,905	No
3,000 vs. 4,000	0,144	0,167	0,905	No

Comparisons for factor: **Month**

Comparison	Diff of Means	t	P	P<0,050
June vs. July	1,008	1,510	0,363	No
June vs. August	0,604	0,905	0,605	No
August vs. July	0,404	0,606	0,605	No

Comparisons for factor: **Year**

Comparison	Diff of Means	t	P	P<0,050
2017,000 vs. 2016,000	2,763	4,138	<0,001	Yes
2018,000 vs. 2016,000	2,021	3,027	0,009	Yes
2017,000 vs. 2018,000	0,742	1,111	0,274	No

Dependent Variable: **[KAEM_TOT]**
Normality Test (Shapiro-Wilk): Passed (P = 0,975)
Equal Variance Test (Brown-Forsythe): Passed (P = 1,000)

Source of Variation	DF	SS	MS	F	P
Individual	4	64,385	16,096	44,242	<0,001
Month	2	0,588	0,294	0,809	0,453
Year	2	1,748	0,874	2,402	0,105
Residual	36	13,098	0,364		
Total	44	79,818	1,814		

Comparisons for factor: **Individual**

Comparison	Diff of Means	t	P	P<0,050
1,000 vs. 5,000	3,260	11,465	<0,001	Yes
1,000 vs. 4,000	3,189	11,216	<0,001	Yes
1,000 vs. 3,000	2,683	9,436	<0,001	Yes
1,000 vs. 2,000	2,196	7,723	<0,001	Yes
2,000 vs. 5,000	1,064	3,742	0,004	Yes
2,000 vs. 4,000	0,993	3,493	0,006	Yes
3,000 vs. 5,000	0,577	2,029	0,185	No
3,000 vs. 4,000	0,506	1,780	0,230	No
2,000 vs. 3,000	0,487	1,713	0,230	No
4,000 vs. 5,000	0,0710	0,250	0,804	No

Comparisons for factor: **Month**

Comparison	Diff of Means	t	P	P<0,050
June vs. July	0,270	1,226	0,540	No
August vs. July	0,200	0,906	0,604	No
June vs. August	0,0703	0,319	0,751	No

Comparisons for factor: **Year**

Comparison	Diff of Means	t	P	P<0,050
2018,000 vs. 2017,000	0,478	2,169	0,106	No
2018,000 vs. 2016,000	0,299	1,359	0,332	No
2016,000 vs. 2017,000	0,178	0,810	0,423	No

Dependent Variable**: [QUER_TOT]**
Normality Test (Shapiro-Wilk): Passed (P = 0,974)
Equal Variance Test (Brown-Forsythe): Passed (P = 1,000)

Source of Variation	DF	SS	MS	F	P
Individual	4	5,996	1,499	1,945	0,124
Month	2	8,674	4,337	5,627	0,007
Year	2	14,441	7,221	9,368	<0,001
Residual	36	27,747	0,771		
Total	44	56,858	1,292		

Comparisons for factor: **Individual**

Comparison	Diff of Means	t	P	P<0,050
2,000 vs. 1,000	1,072	2,591	0,129	No
4,000 vs. 1,000	0,797	1,925	0,439	No
2,000 vs. 5,000	0,665	1,606	0,631	No
2,000 vs. 3,000	0,622	1,502	0,657	No
3,000 vs. 1,000	0,451	1,089	0,865	No
5,000 vs. 1,000	0,408	0,985	0,866	No
4,000 vs. 5,000	0,389	0,940	0,866	No
4,000 vs. 3,000	0,346	0,836	0,866	No
2,000 vs. 4,000	0,276	0,666	0,866	No
3,000 vs. 5,000	0,0430	0,104	0,918	No

Comparisons for factor: **Month**

Comparison	Diff of Means	t	P	P<0,050
June vs. August	1,049	3,271	0,007	Yes
June vs. July	0,731	2,280	0,056	No
July vs. August	0,318	0,991	0,328	No

Comparisons for factor: **Year**

Comparison	Diff of Means	t	P	P<0,050
2017,000 vs. 2016,000	1,376	4,291	<0,001	Yes
2018,000 vs. 2016,000	0,845	2,637	0,024	Yes
2017,000 vs. 2018,000	0,530	1,654	0,107	No

Dependent Variable: **[MYRI_TOT]**
Normality Test (Shapiro-Wilk): Passed (P = 0,078)
Equal Variance Test (Brown-Forsythe): Passed (P = 1,000)

Source of Variation	DF	SS	MS	F	P
Individual	4	0,926	0,231	18,396	<0,001
Month	2	0,0134	0,00668	0,531	0,593
Year	2	0,0477	0,0238	1,896	0,165
Residual	36	0,453	0,0126		
Total	44	1,440	0,0327		

Comparisons for factor: **Individual**

Comparison	Diff of Means	t	P	P<0,050
5,000 vs. 4,000	0,422	7,989	<0,001	Yes
5,000 vs. 3,000	0,347	6,563	<0,001	Yes
5,000 vs. 2,000	0,299	5,649	<0,001	Yes
5,000 vs. 1,000	0,271	5,127	<0,001	Yes
1,000 vs. 4,000	0,151	2,861	0,041	Yes
2,000 vs. 4,000	0,124	2,339	0,119	No
1,000 vs. 3,000	0,0759	1,436	0,501	No
3,000 vs. 4,000	0,0754	1,425	0,501	No
2,000 vs. 3,000	0,0483	0,914	0,599	No
1,000 vs. 2,000	0,0276	0,522	0,605	No

Comparisons for factor: **Month**

Comparison	Diff of Means	t	P	P<0,050
August vs. July	0,0421	1,029	0,672	No
June vs. July	0,0229	0,560	0,823	No
August vs. June	0,0192	0,469	0,823	No

Comparisons for factor: **Year**

Comparison	Diff of Means	t	P	P<0,050
2018,000 vs. 2016,000	0,0728	1,778	0,231	No
2017,000 vs. 2016,000	0,0646	1,577	0,232	No
2018,000 vs. 2017,000	0,00824	0,201	0,842	No

Table S20. The results of statistical analyses of *Prunus padus*. The differences in the mean values of quantitative results of each polyphenol group between individual trees, years and sampling times were analyzed using three-way analysis of variance (Three-way ANOVA). Pairwise multiple comparison procedures were performed with Holm-Sidak method, when significant differences were detected. The significance level was set to p=0.05.

Dependent Variable: **[GALL_TOT]**
Normality Test (Shapiro-Wilk): Failed (P < 0,050)
Equal Variance Test (Brown-Forsythe): Passed (P = 1,000)

Source of Variation	DF	SS	MS	F	P
Individual	4	0,000	0,000	0,000	1,000
Month	2	0,000	0,000	0,000	1,000
Year	2	0,000	0,000	0,000	1,000
Residual	36	0,000	0,000		
Total	44	0,000	0,000		

Dependent Variable: **[HHDP_TOT]**
Normality Test (Shapiro-Wilk): Failed (P < 0,050)
Equal Variance Test (Brown-Forsythe): Passed (P = 1,000)

Source of Variation	DF	SS	MS	F	P
Individual	4	0,000	0,000	0,000	1,000
Month	2	0,000	0,000	0,000	1,000
Year	2	0,000	0,000	0,000	1,000
Residual	36	0,000	0,000		
Total	44	0,000	0,000		

Dependent Variable: **[PC_TOT]**
Normality Test (Shapiro-Wilk): Passed (P = 0,584)
Equal Variance Test (Brown-Forsythe): Passed (P = 1,000)

Source of Variation	DF	SS	MS	F	P
Individual	4	90,447	22,612	0,743	0,569
Month	2	168,535	84,268	2,770	0,076
Year	2	272,446	136,223	4,477	0,018
Residual	36	1095,369	30,427		
Total	44	1626,798	36,973		

Comparisons for factor: **Individual**

Comparison	Diff of Means	t	P	P<0,050
5,000 vs. 3,000	3,358	1,291	0,899	No
2,000 vs. 3,000	3,175	1,221	0,905	No
5,000 vs. 4,000	2,944	1,132	0,915	No
2,000 vs. 4,000	2,762	1,062	0,915	No

1,000 vs. 3,000	2,592	0,997	0,915	No
1,000 vs. 4,000	2,179	0,838	0,927	No
5,000 vs. 1,000	0,766	0,294	0,997	No
2,000 vs. 1,000	0,583	0,224	0,997	No
4,000 vs. 3,000	0,413	0,159	0,997	No
5,000 vs. 2,000	0,182	0,0701	0,997	No

Comparisons for factor: **Month**

**Comparison**	**Diff of Means**	**t**	**P**	**P<0,050**
August vs. June	4,684	2,326	0,075	No
July vs. June	2,972	1,476	0,275	No
August vs. July	1,712	0,850	0,401	No

Comparisons for factor: **Year**

**Comparison**	**Diff of Means**	**t**	**P**	**P<0,050**
2018,000 vs. 2017,000	5,758	2,859	0,021	Yes
2018,000 vs. 2016,000	4,421	2,195	0,068	No
2016,000 vs. 2017,000	1,338	0,664	0,511	No

Dependent Variable: **[PD_TOT]**
Normality Test (Shapiro-Wilk): Failed (P < 0,050)
Equal Variance Test (Brown-Forsythe): Passed (P = 1,000)

**Source of Variation**	**DF**	**SS**	**MS**	**F**	**P**
Individual	4	0,000	0,000	0,000	1,000
Month	2	0,000	0,000	0,000	1,000
Year	2	0,000	0,000	0,000	1,000
Residual	36	0,000	0,000		
Total	44	0,000	0,000		

Dependent Variable**: [QUIN_TOT]**
Normality Test (Shapiro-Wilk): Passed (P = 0,526)
Equal Variance Test (Brown-Forsythe): Passed (P = 1,000)

**Source of Variation**	**DF**	**SS**	**MS**	**F**	**P**
Individual	4	1,243	0,311	0,565	0,690
Month	2	16,752	8,376	15,225	<0,001
Year	2	15,598	7,799	14,177	<0,001
Residual	36	19,805	0,550		
Total	44	53,398	1,214		

Comparisons for factor: **Individual**

**Comparison**	**Diff of Means**	**t**	**P**	**P<0,050**
4,000 vs. 2,000	0,495	1,417	0,836	No
4,000 vs. 1,000	0,385	1,102	0,947	No
4,000 vs. 3,000	0,336	0,961	0,965	No
4,000 vs. 5,000	0,261	0,746	0,987	No
5,000 vs. 2,000	0,234	0,670	0,987	No
3,000 vs. 2,000	0,159	0,455	0,995	No
5,000 vs. 1,000	0,124	0,355	0,995	No
1,000 vs. 2,000	0,110	0,315	0,995	No
5,000 vs. 3,000	0,0752	0,215	0,995	No
3,000 vs. 1,000	0,0491	0,140	0,995	No

Comparisons for factor: **Month**

**Comparison**	**Diff of Means**	**t**	**P**	**P<0,050**
June vs. August	1,458	5,382	<0,001	Yes
July vs. August	1,014	3,745	0,001	Yes
June vs. July	0,443	1,637	0,110	No

Comparisons for factor: **Year**

**Comparison**	**Diff of Means**	**t**	**P**	**P<0,050**
2016,000 vs. 2018,000	1,384	5,109	<0,001	Yes
2017,000 vs. 2018,000	1,044	3,853	<0,001	Yes
2016,000 vs. 2017,000	0,340	1,256	0,217	No

Dependent Variable: **[KAEM_TOT]**
Normality Test (Shapiro-Wilk): Passed (P = 0,115)
Equal Variance Test (Brown-Forsythe): Passed (P = 1,000)

**Source of Variation**	**DF**	**SS**	**MS**	**F**	**P**

Individual	4	0,667	0,167	18,715	<0,001
Month	2	0,0934	0,0467	5,242	0,010
Year	2	0,179	0,0895	10,046	<0,001
Residual	36	0,321	0,00890		
Total	44	1,259	0,0286		

Comparisons for factor: **Individual**

**Comparison**	**Diff of Means**	**t**	**P**	**P<0,050**
2,000 vs. 1,000	0,318	7,157	<0,001	Yes
2,000 vs. 3,000	0,297	6,669	<0,001	Yes
2,000 vs. 5,000	0,247	5,550	<0,001	Yes
4,000 vs. 1,000	0,212	4,759	<0,001	Yes
4,000 vs. 3,000	0,190	4,271	<0,001	Yes
4,000 vs. 5,000	0,140	3,153	0,016	Yes
2,000 vs. 4,000	0,107	2,398	0,084	No
5,000 vs. 1,000	0,0715	1,606	0,311	No
5,000 vs. 3,000	0,0498	1,119	0,468	No
3,000 vs. 1,000	0,0217	0,488	0,629	No

Comparisons for factor: **Month**

**Comparison**	**Diff of Means**	**t**	**P**	**P<0,050**
June vs. July	0,110	3,188	0,009	Yes
June vs. August	0,0718	2,084	0,087	No
August vs. July	0,0381	1,104	0,277	No

Comparisons for factor: **Year**

**Comparison**	**Diff of Means**	**t**	**P**	**P<0,050**
2016,000 vs. 2018,000	0,145	4,197	<0,001	Yes
2016,000 vs. 2017,000	0,119	3,461	0,003	Yes
2017,000 vs. 2018,000	0,0254	0,736	0,466	No

Dependent Variable: **[QUER_TOT]**
Normality Test (Shapiro-Wilk): Passed (P = 0,517)
Equal Variance Test (Brown-Forsythe): Passed (P = 1,000)

**Source of Variation**	**DF**	**SS**	**MS**	**F**	**P**
Individual	4	14,999	3,750	10,822	<0,001
Month	2	4,470	2,235	6,450	0,004
Year	2	10,820	5,410	15,613	<0,001
Residual	36	12,474	0,346		
Total	44	42,762	0,972		

Comparisons for factor: **Individual**

**Comparison**	**Diff of Means**	**t**	**P**	**P<0,050**
1,000 vs. 3,000	1,692	6,097	<0,001	Yes
2,000 vs. 3,000	1,427	5,143	<0,001	Yes
5,000 vs. 3,000	1,119	4,032	0,002	Yes
4,000 vs. 3,000	0,977	3,522	0,008	Yes
1,000 vs. 4,000	0,714	2,575	0,083	No
1,000 vs. 5,000	0,573	2,066	0,210	No
2,000 vs. 4,000	0,450	1,620	0,383	No
2,000 vs. 5,000	0,308	1,111	0,617	No
1,000 vs. 2,000	0,265	0,954	0,617	No
5,000 vs. 4,000	0,141	0,509	0,617	No

Comparisons for factor: **Month**

**Comparison**	**Diff of Means**	**t**	**P**	**P<0,050**
June vs. August	0,711	3,309	0,006	Yes
June vs. July	0,616	2,864	0,014	Yes
July vs. August	0,0958	0,446	0,659	No

Comparisons for factor: **Year**

**Comparison**	**Diff of Means**	**t**	**P**	**P<0,050**
2017,000 vs. 2018,000	1,070	4,976	<0,001	Yes
2016,000 vs. 2018,000	1,008	4,690	<0,001	Yes
2017,000 vs. 2016,000	0,0615	0,286	0,776	No

Dependent Variable: **[MYRI_TOT]**
Normality Test (Shapiro-Wilk): Failed (P < 0,050)
Equal Variance Test (Brown-Forsythe): Passed (P = 1,000)

**Source of Variation**	**DF**	**SS**	**MS**	**F**	**P**

Individual	4	0,000	0,000	0,000	1,000
Month	2	0,000	0,000	0,000	1,000
Year	2	0,000	0,000	0,000	1,000
Residual	36	0,000	0,000		
Total	44	0,000	0,000		

Table S21. The results of statistical analyses of *Juniperus communis*. The differences in the mean values of quantitative results of each polyphenol group between individual trees, years and sampling times were analyzed using three-way analysis of variance (Three-way ANOVA). Pairwise multiple comparison procedures were performed with Holm-Sidak method, when significant differences were detected. The significance level was set to p=0.05.

Dependent Variable: **[GALL_TOT]**
Normality Test (Shapiro-Wilk): Failed (P < 0,050)
Equal Variance Test (Brown-Forsythe): Passed (P = 1,000)

**Source of Variation**	**DF**	**SS**	**MS**	**F**	**P**
Individual	4	0,000	0,000	0,000	1,000
Month	2	0,000	0,000	0,000	1,000
Year	2	0,000	0,000	0,000	1,000
Residual	36	0,000	0,000		
Total	44	0,000	0,000		

Dependent Variable: **[HHDP_TOT]**
Normality Test (Shapiro-Wilk): Failed (P < 0,050)
Equal Variance Test (Brown-Forsythe): Passed (P = 1,000)

**Source of Variation**	**DF**	**SS**	**MS**	**F**	**P**
Individual	4	0,000	0,000	0,000	1,000
Month	2	0,000	0,000	0,000	1,000
Year	2	0,000	0,000	0,000	1,000
Residual	36	0,000	0,000		
Total	44	0,000	0,000		

Dependent Variable: **[PC_TOT]**
Normality Test (Shapiro-Wilk): Passed (P = 0,118)
Equal Variance Test (Brown-Forsythe): Passed (P = 1,000)

**Source of Variation**	**DF**	**SS**	**MS**	**F**	**P**
Individual	4	376,321	94,080	9,072	<0,001
Month	2	108,447	54,223	5,229	0,010
Year	2	110,761	55,381	5,340	0,009
Residual	36	373,338	10,370		
Total	44	968,867	22,020		

Comparisons for factor: **Individual**

**Comparison**	**Diff of Means**	**t**	**P**	**P<0,050**
3,000 vs. 2,000	8,650	5,698	<0,001	Yes
5,000 vs. 2,000	6,183	4,073	0,002	Yes
3,000 vs. 1,000	5,305	3,495	0,010	Yes
3,000 vs. 4,000	4,578	3,015	0,032	Yes
4,000 vs. 2,000	4,073	2,683	0,064	No
1,000 vs. 2,000	3,345	2,204	0,159	No
5,000 vs. 1,000	2,838	1,870	0,251	No
3,000 vs. 5,000	2,467	1,625	0,302	No
5,000 vs. 4,000	2,111	1,390	0,316	No
4,000 vs. 1,000	0,727	0,479	0,635	No

Comparisons for factor: **Month**

**Comparison**	**Diff of Means**	**t**	**P**	**P<0,050**
August vs. June	3,713	3,158	0,010	Yes
August vs. July	2,565	2,182	0,070	No
July vs. June	1,148	0,976	0,335	No

Comparisons for factor: **Year**

**Comparison**	**Diff of Means**	**t**	**P**	**P<0,050**
2016,000 vs. 2018,000	3,806	3,237	0,008	Yes
2017,000 vs. 2018,000	2,362	2,009	0,102	No
2016,000 vs. 2017,000	1,444	1,228	0,227	No

Dependent Variable**: [PD_TOT]**
Normality Test (Shapiro-Wilk): Passed (P = 0,454)
Equal Variance Test (Brown-Forsythe): Passed (P = 1,000)

**Source of Variation**	**DF**	**SS**	**MS**	**F**	**P**
Individual	4	24,913	6,228	7,668	<0,001
Month	2	0,568	0,284	0,349	0,707
Year	2	1,448	0,724	0,891	0,419
Residual	36	29,239	0,812		
Total	44	56,167	1,277		

Comparisons for factor: **Individual**

**Comparison**	**Diff of Means**	**t**	**P**	**P<0,050**
4,000 vs. 1,000	1,955	4,601	<0,001	Yes
2,000 vs. 1,000	1,687	3,971	0,003	Yes
4,000 vs. 3,000	1,575	3,706	0,006	Yes
2,000 vs. 3,000	1,307	3,076	0,028	Yes
5,000 vs. 1,000	1,021	2,403	0,122	No
4,000 vs. 5,000	0,934	2,198	0,161	No
2,000 vs. 5,000	0,666	1,568	0,416	No
5,000 vs. 3,000	0,641	1,509	0,416	No
3,000 vs. 1,000	0,380	0,895	0,612	No
4,000 vs. 2,000	0,268	0,630	0,612	No

Comparisons for factor: **Month**

**Comparison**	**Diff of Means**	**t**	**P**	**P<0,050**
July vs. June	0,251	0,764	0,833	No
July vs. August	0,222	0,676	0,833	No
August vs. June	0,0290	0,0881	0,930	No

Comparisons for factor: **Year**

**Comparison**	**Diff of Means**	**t**	**P**	**P<0,050**
2017,000 vs. 2018,000	0,385	1,171	0,577	No
2017,000 vs. 2016,000	0,376	1,141	0,577	No
2016,000 vs. 2018,000	0,00966	0,0294	0,977	No

Dependent Variable**: [QUIN_TOT]**
Normality Test (Shapiro-Wilk): Passed (P = 0,680)
Equal Variance Test (Brown-Forsythe): Passed (P = 1,000)

**Source of Variation**	**DF**	**SS**	**MS**	**F**	**P**
Individual	4	2,067	0,517	38,262	<0,001
Month	2	2,759	1,380	102,179	<0,001
Year	2	0,105	0,0524	3,880	0,030
Residual	36	0,486	0,0135		
Total	44	5,417	0,123		

Comparisons for factor: **Individual**

**Comparison**	**Diff of Means**	**t**	**P**	**P<0,050**
2,000 vs. 3,000	0,561	10,250	<0,001	Yes
1,000 vs. 3,000	0,533	9,732	<0,001	Yes
2,000 vs. 4,000	0,379	6,916	<0,001	Yes
1,000 vs. 4,000	0,350	6,397	<0,001	Yes
2,000 vs. 5,000	0,318	5,811	<0,001	Yes
1,000 vs. 5,000	0,290	5,292	<0,001	Yes
5,000 vs. 3,000	0,243	4,439	<0,001	Yes
4,000 vs. 3,000	0,183	3,334	0,006	Yes
5,000 vs. 4,000	0,0605	1,105	0,477	No
2,000 vs. 1,000	0,0284	0,519	0,607	No

Comparisons for factor: **Month**

**Comparison**	**Diff of Means**	**t**	**P**	**P<0,050**
June vs. August	0,582	13,710	<0,001	Yes
June vs. July	0,440	10,362	<0,001	Yes
July vs. August	0,142	3,347	0,002	Yes

Comparisons for factor: **Year**

**Comparison**	**Diff of Means**	**t**	**P**	**P<0,050**
2016,000 vs. 2017,000	0,105	2,477	0,053	No
2016,000 vs. 2018,000	0,0994	2,343	0,053	No
2018,000 vs. 2017,000	0,00568	0,134	0,894	No

Dependent Variable: **[KAEM_TOT]**
Normality Test (Shapiro-Wilk): Failed (P < 0,050)
Equal Variance Test (Brown-Forsythe): Passed (P = 1,000)

**Source of Variation**	**DF**	**SS**	**MS**	**F**	**P**
Individual	4	0,222	0,0556	4,861	0,003
Month	2	0,399	0,199	17,446	<0,001
Year	2	0,323	0,162	14,150	<0,001
Residual	36	0,411	0,0114		
Total	44	1,356	0,0308		

Comparisons for factor: **Individual**

**Comparison**	**Diff of Means**	**t**	**P**	**P<0,050**
4,000 vs. 1,000	0,214	4,252	0,001	Yes
2,000 vs. 1,000	0,149	2,961	0,048	Yes
5,000 vs. 1,000	0,127	2,528	0,121	No
4,000 vs. 3,000	0,117	2,324	0,168	No
3,000 vs. 1,000	0,0971	1,928	0,318	No
4,000 vs. 5,000	0,0869	1,724	0,387	No
4,000 vs. 2,000	0,0650	1,290	0,601	No
2,000 vs. 3,000	0,0521	1,034	0,669	No
5,000 vs. 3,000	0,0303	0,600	0,799	No
2,000 vs. 5,000	0,0218	0,433	0,799	No

Comparisons for factor: **Month**

**Comparison**	**Diff of Means**	**t**	**P**	**P<0,050**
June vs. July	0,211	5,394	<0,001	Yes
June vs. August	0,187	4,782	<0,001	Yes
August vs. July	0,0239	0,613	0,544	No

Comparisons for factor: **Year**

**Comparison**	**Diff of Means**	**t**	**P**	**P<0,050**
2016,000 vs. 2017,000	0,188	4,824	<0,001	Yes
2016,000 vs. 2018,000	0,170	4,354	<0,001	Yes
2018,000 vs. 2017,000	0,0183	0,470	0,641	No

Dependent Variable: **[QUER_TOT]**
Normality Test (Shapiro-Wilk): Passed (P = 0,486)
Equal Variance Test (Brown-Forsythe): Passed (P = 1,000)

**Source of Variation**	**DF**	**SS**	**MS**	**F**	**P**
Individual	4	1,106	0,276	9,962	<0,001
Month	2	9,240	4,620	166,487	<0,001
Year	2	0,0929	0,0465	1,674	0,202
Residual	36	0,999	0,0278		
Total	44	11,438	0,260		

Comparisons for factor: **Individual**

**Comparison**	**Diff of Means**	**t**	**P**	**P<0,050**
2,000 vs. 1,000	0,421	5,358	<0,001	Yes
5,000 vs. 1,000	0,409	5,207	<0,001	Yes
4,000 vs. 1,000	0,304	3,877	0,003	Yes
2,000 vs. 3,000	0,234	2,980	0,035	Yes
5,000 vs. 3,000	0,222	2,829	0,045	Yes
3,000 vs. 1,000	0,187	2,378	0,109	No
4,000 vs. 3,000	0,118	1,498	0,460	No
2,000 vs. 4,000	0,116	1,482	0,460	No
5,000 vs. 4,000	0,104	1,330	0,460	No
2,000 vs. 5,000	0,0119	0,151	0,881	No

Comparisons for factor: **Month**

**Comparison**	**Diff of Means**	**t**	**P**	**P<0,050**
June vs. August	1,037	17,055	<0,001	Yes
June vs. July	0,860	14,146	<0,001	Yes
July vs. August	0,177	2,909	0,006	Yes

Comparisons for factor: **Year**

**Comparison**	**Diff of Means**	**t**	**P**	**P<0,050**
2018,000 vs. 2016,000	0,106	1,744	0,246	No
2018,000 vs. 2017,000	0,0823	1,353	0,335	No
2017,000 vs. 2016,000	0,0238	0,391	0,698	No

Dependent Variable: **[MYRI_TOT]**
Normality Test (Shapiro-Wilk): Failed (P < 0,050)
Equal Variance Test (Brown-Forsythe): Passed (P = 1,000)

**Source of Variation**	**DF**	**SS**	**MS**	**F**	**P**
Individual	4	0,173	0,0432	14,322	<0,001
Month	2	0,0364	0,0182	6,037	0,005
Year	2	0,00906	0,00453	1,502	0,236
Residual	36	0,109	0,00302		
Total	44	0,327	0,00743		

Comparisons for factor: **Individual**

**Comparison**	**Diff of Means**	**t**	**P**	**P<0,050**
2,000 vs. 1,000	0,169	6,528	<0,001	Yes
4,000 vs. 1,000	0,161	6,226	<0,001	Yes
2,000 vs. 3,000	0,0923	3,564	0,008	Yes
5,000 vs. 1,000	0,0874	3,375	0,012	Yes
4,000 vs. 3,000	0,0845	3,261	0,014	Yes
2,000 vs. 5,000	0,0817	3,153	0,016	Yes
3,000 vs. 1,000	0,0768	2,964	0,021	Yes
4,000 vs. 5,000	0,0738	2,851	0,021	Yes
5,000 vs. 3,000	0,0106	0,410	0,900	No
2,000 vs. 4,000	0,00783	0,302	0,900	No

Comparisons for factor: **Month**

**Comparison**	**Diff of Means**	**t**	**P**	**P<0,050**
June vs. August	0,0687	3,424	0,005	Yes
July vs. August	0,0446	2,224	0,064	No
June vs. July	0,0241	1,201	0,238	No

Comparisons for factor: **Year**

**Comparison**	**Diff of Means**	**t**	**P**	**P<0,050**
2016,000 vs. 2018,000	0,0317	1,581	0,325	No
2016,000 vs. 2017,000	0,0282	1,405	0,325	No
2017,000 vs. 2018,000	0,00353	0,176	0,861	No

Table S22. The results of statistical analyses of *Picea abies*. The differences in the mean values of quantitative results of each polyphenol group between individual trees, years and sampling times were analyzed using three-way analysis of variance (Three-way ANOVA). Pairwise multiple comparison procedures were performed with Holm-Sidak method, when significant differences were detected. The significance level was set to p=0.05.

Dependent Variable: **[GALL_TOT]**
Normality Test (Shapiro-Wilk): Failed (P < 0,050)
Equal Variance Test (Brown-Forsythe): Passed (P = 1,000)

**Source of Variation**	**DF**	**SS**	**MS**	**F**	**P**
Individual	4	0,000	0,000	0,000	1,000
Month	2	0,000	0,000	0,000	1,000
Year	2	0,000	0,000	0,000	1,000
Residual	36	0,000	0,000		
Total	44	0,000	0,000		

Dependent Variable: **[HHDP_TOT]**
Normality Test (Shapiro-Wilk): Failed (P < 0,050)
Equal Variance Test (Brown-Forsythe): Passed (P = 1,000)

**Source of Variation**	**DF**	**SS**	**MS**	**F**	**P**
Individual	4	0,000	0,000	0,000	1,000
Month	2	0,000	0,000	0,000	1,000
Year	2	0,000	0,000	0,000	1,000
Residual	36	0,000	0,000		
Total	44	0,000	0,000		

Dependent Variable: **[PC_TOT]**
Normality Test (Shapiro-Wilk): Passed (P = 0,697)
Equal Variance Test (Brown-Forsythe): Passed (P = 1,000)

Source of Variation	DF	SS	MS	F	P
Individual	4	1747,810	436,952	14,128	<0,001
Month	2	345,670	172,835	5,588	0,008
Year	2	20,226	10,113	0,327	0,723
Residual	36	1113,383	30,927		
Total	44	3227,089	73,343		

Comparisons for factor: **Individual**

Comparison	Diff of Means	t	P	P<0,050
1,000 vs. 2,000	18,586	7,089	<0,001	Yes
4,000 vs. 2,000	14,918	5,690	<0,001	Yes
5,000 vs. 2,000	10,811	4,124	0,002	Yes
3,000 vs. 2,000	10,466	3,992	0,002	Yes
1,000 vs. 3,000	8,119	3,097	0,022	Yes
1,000 vs. 5,000	7,775	2,966	0,026	Yes
4,000 vs. 3,000	4,452	1,698	0,338	No
4,000 vs. 5,000	4,107	1,567	0,338	No
1,000 vs. 4,000	3,668	1,399	0,338	No
5,000 vs. 3,000	0,345	0,131	0,896	No

Comparisons for factor: **Month**

Comparison	Diff of Means	t	P	P<0,050
August vs. July	6,775	3,336	0,006	Yes
June vs. July	3,763	1,853	0,139	No
August vs. June	3,012	1,483	0,147	No

Comparisons for factor: **Year**

Comparison	Diff of Means	t	P	P<0,050
2017,000 vs. 2016,000	1,449	0,714	0,859	No
2017,000 vs. 2018,000	1,394	0,686	0,859	No
2018,000 vs. 2016,000	0,0552	0,0272	0,978	No

Dependent Variable: **[PD_TOT]**
Normality Test (Shapiro-Wilk): Failed (P < 0,050)
Equal Variance Test (Brown-Forsythe): Passed (P = 1,000)

Source of Variation	DF	SS	MS	F	P
Individual	4	111,634	27,908	8,383	<0,001
Month	2	18,597	9,299	2,793	0,075
Year	2	4,556	2,278	0,684	0,511
Residual	36	119,856	3,329		
Total	44	254,643	5,787		

Comparisons for factor: **Individual**

Comparison	Diff of Means	t	P	P<0,050
2,000 vs. 1,000	4,436	5,157	<0,001	Yes
2,000 vs. 3,000	4,162	4,839	<0,001	Yes
2,000 vs. 5,000	3,036	3,530	0,009	Yes
2,000 vs. 4,000	2,738	3,183	0,021	Yes
4,000 vs. 1,000	1,698	1,974	0,293	No
4,000 vs. 3,000	1,424	1,656	0,430	No
5,000 vs. 1,000	1,399	1,627	0,430	No
5,000 vs. 3,000	1,126	1,309	0,486	No
4,000 vs. 5,000	0,298	0,347	0,928	No
3,000 vs. 1,000	0,273	0,318	0,928	No

Comparisons for factor: **Month**

Comparison	Diff of Means	t	P	P<0,050
June vs. July	1,471	2,207	0,098	No
August vs. July	1,223	1,835	0,144	No
June vs. August	0,248	0,372	0,712	No

Comparisons for factor: **Year**

Comparison	Diff of Means	t	P	P<0,050
2017,000 vs. 2018,000	0,688	1,032	0,670	No
2017,000 vs. 2016,000	0,661	0,993	0,670	No
2016,000 vs. 2018,000	0,0265	0,0397	0,969	No

Dependent Variable: **[QUIN_TOT]**
Normality Test (Shapiro-Wilk): Failed (P < 0,050)
Equal Variance Test (Brown-Forsythe): Passed (P = 1,000)

Source of Variation	DF	SS	MS	F	P
Individual	4	1,883	0,471	22,728	<0,001
Month	2	1,588	0,794	38,338	<0,001
Year	2	0,258	0,129	6,238	0,005
Residual	36	0,746	0,0207		
Total	44	4,475	0,102		

Comparisons for factor: **Individual**

Comparison	Diff of Means	t	P	P<0,050
1,000 vs. 4,000	0,559	8,234	<0,001	Yes
1,000 vs. 3,000	0,531	7,834	<0,001	Yes
1,000 vs. 5,000	0,471	6,947	<0,001	Yes
1,000 vs. 2,000	0,441	6,502	<0,001	Yes
2,000 vs. 4,000	0,117	1,732	0,439	No
2,000 vs. 3,000	0,0904	1,332	0,654	No
5,000 vs. 4,000	0,0873	1,287	0,654	No
5,000 vs. 3,000	0,0602	0,887	0,763	No
2,000 vs. 5,000	0,0302	0,445	0,884	No
3,000 vs. 4,000	0,0271	0,399	0,884	No

Comparisons for factor: **Month**

Comparison	Diff of Means	t	P	P<0,050
June vs. August	0,427	8,117	<0,001	Yes
June vs. July	0,363	6,904	<0,001	Yes
July vs. August	0,0638	1,213	0,233	No

Comparisons for factor: **Year**

Comparison	Diff of Means	t	P	P<0,050
2016,000 vs. 2018,000	0,161	3,061	0,012	Yes
2016,000 vs. 2017,000	0,161	3,057	0,012	Yes
2017,000 vs. 2018,000	0,000186	0,00353	0,997	No

Dependent Variable: **[KAEM_TOT]**
Normality Test (Shapiro-Wilk): Passed (P = 0,249)
Equal Variance Test (Brown-Forsythe): Passed (P = 1,000)

Source of Variation	DF	SS	MS	F	P
Individual	4	2,866	0,716	0,927	0,459
Month	2	57,898	28,949	37,441	<0,001
Year	2	15,683	7,842	10,142	<0,001
Residual	36	27,835	0,773		
Total	44	104,282	2,370		

Comparisons for factor: **Individual**

Comparison	Diff of Means	t	P	P<0,050
2,000 vs. 3,000	0,766	1,848	0,531	No
2,000 vs. 5,000	0,568	1,370	0,831	No
2,000 vs. 4,000	0,500	1,207	0,883	No
2,000 vs. 1,000	0,458	1,105	0,896	No
1,000 vs. 3,000	0,308	0,743	0,976	No
4,000 vs. 3,000	0,265	0,640	0,976	No
5,000 vs. 3,000	0,198	0,478	0,982	No
1,000 vs. 5,000	0,110	0,265	0,991	No
4,000 vs. 5,000	0,0672	0,162	0,991	No
1,000 vs. 4,000	0,0425	0,102	0,991	No

Comparisons for factor: **Month**

Comparison	Diff of Means	t	P	P<0,050
June vs. July	2,468	7,687	<0,001	Yes
June vs. August	2,339	7,285	<0,001	Yes
August vs. July	0,129	0,402	0,690	No

Comparisons for factor: **Year**

Comparison	Diff of Means	t	P	P<0,050
2016,000 vs. 2018,000	1,434	4,467	<0,001	Yes
2017,000 vs. 2018,000	0,877	2,732	0,019	Yes
2016,000 vs. 2017,000	0,557	1,735	0,091	No

Dependent Variable**: [QUER_TOT]**
Normality Test (Shapiro-Wilk): Passed (P = 0,471)
Equal Variance Test (Brown-Forsythe): Passed (P = 1,000)

Source of Variation	DF	SS	MS	F	P
Individual	4	0,997	0,249	4,294	0,006
Month	2	5,644	2,822	48,637	<0,001
Year	2	0,885	0,442	7,626	0,002
Residual	36	2,089	0,0580		
Total	44	9,614	0,218		

Comparisons for factor: **Individual**

Comparison	Diff of Means	t	P	P<0,050
1,000 vs. 3,000	0,427	3,761	0,006	Yes
1,000 vs. 2,000	0,303	2,665	0,098	No
5,000 vs. 3,000	0,301	2,651	0,098	No
1,000 vs. 4,000	0,284	2,503	0,113	No
5,000 vs. 2,000	0,177	1,555	0,563	No
5,000 vs. 4,000	0,158	1,392	0,612	No
4,000 vs. 3,000	0,143	1,258	0,623	No
1,000 vs. 5,000	0,126	1,111	0,623	No
2,000 vs. 3,000	0,124	1,096	0,623	No
4,000 vs. 2,000	0,0184	0,162	0,872	No

Comparisons for factor: **Month**

Comparison	Diff of Means	t	P	P<0,050
June vs. August	0,756	8,594	<0,001	Yes
June vs. July	0,746	8,487	<0,001	Yes
July vs. August	0,00942	0,107	0,915	No

Comparisons for factor: **Year**

Comparison	Diff of Means	t	P	P<0,050
2016,000 vs. 2018,000	0,343	3,905	0,001	Yes
2017,000 vs. 2018,000	0,174	1,976	0,109	No
2016,000 vs. 2017,000	0,170	1,929	0,109	No

Dependent Variable: **[MYRI_TOT]**
Normality Test (Shapiro-Wilk): Passed (P = 0,063)
Equal Variance Test (Brown-Forsythe): Passed (P = 1,000)

Source of Variation	DF	SS	MS	F	P
Individual	4	0,148	0,0369	4,337	0,006
Month	2	0,748	0,374	43,937	<0,001
Year	2	0,163	0,0815	9,578	<0,001
Residual	36	0,306	0,00851		
Total	44	1,365	0,0310		

Comparisons for factor: **Individual**

Comparison	Diff of Means	t	P	P<0,050
4,000 vs. 2,000	0,168	3,871	0,004	Yes
5,000 vs. 2,000	0,125	2,873	0,059	No
1,000 vs. 2,000	0,114	2,611	0,100	No
4,000 vs. 3,000	0,101	2,319	0,169	No
3,000 vs. 2,000	0,0675	1,552	0,565	No
5,000 vs. 3,000	0,0574	1,321	0,662	No
4,000 vs. 1,000	0,0548	1,260	0,662	No
1,000 vs. 3,000	0,0461	1,059	0,662	No
4,000 vs. 5,000	0,0434	0,998	0,662	No
5,000 vs. 1,000	0,0114	0,262	0,795	No

Comparisons for factor: **Month**

Comparison	Diff of Means	t	P	P<0,050
June vs. August	0,292	8,676	<0,001	Yes
June vs. July	0,250	7,412	<0,001	Yes
July vs. August	0,0426	1,264	0,214	No

Comparisons for factor: **Year**

Comparison	Diff of Means	t	P	P<0,050
2016,000 vs. 2018,000	0,147	4,377	<0,001	Yes
2017,000 vs. 2018,000	0,0741	2,200	0,067	No
2016,000 vs. 2017,000	0,0733	2,176	0,067	No
